# Origin of the temperature dependence of ^13^C pNMR shifts for copper paddlewheel MOFs†

**DOI:** 10.1039/d1sc07138f

**Published:** 2022-02-03

**Authors:** Zhipeng Ke, Daniel M. Dawson, Sharon E. Ashbrook, Michael Bühl

**Affiliations:** School of Chemistry, EaStCHEM and Centre of Magnetic Resonance, University of St Andrews St Andrews KY16 9ST UK sema@st-andrews.ac.uk mb105@st-andrews.ac.uk

## Abstract

An efficient protocol for the calculation of ^13^C pNMR shifts in metal–organic frameworks based on Cu(ii) paddlewheel dimers is proposed, which involves simplified structural models, optimised using GFN2-xTB for the high-spin state, and CAM-B3LYP-computed NMR and EPR parameters. Models for hydrated and activated HKUST-1 and hydrated STAM MOFs with one, two and three Cu dimers have been used. The electronic ground states are low-spin and diamagnetic, with pNMR shifts arising from thermal population of intermediate- and high-spin excited states. Treating individual spin configurations in a broken symmetry (BS) approach, and selecting two or more of these to describe individual excited states, the magnetic shieldings of these paramagnetic states are evaluated using the approach by Hrobárik and Kaupp. The total shielding is then evaluated from a Boltzmann distribution between the energy levels of the chosen configurations. The computed pNMR shifts are very sensitive to temperature and, therefore, to the relative energies of the BS spin states. In order to reproduce the temperature dependence of the pNMR shifts seen in experiment, some scaling of the calculated energy gaps is required. A single scaling factor was applied to all levels in any one system, by fitting to experimental results at several temperatures simultaneously. The resulting scaling factor decreases with an increasing number of dimer units in the model (*e.g.*, from ∼1.7 for mono-dimer models to 1.2 for tri-dimer models). The approach of this scaling factor towards unity indicates that models with three dimers are approaching a size where they can be considered as reasonable models for the ^13^C shifts of infinite MOFs. The observed unusual temperature dependencies in the latter are indicated to arise both from the “normal” temperature dependence of the pNMR shifts of the paramagnetic states and the populations of these states in the thermal equilibrium.

## Abstract 2

Thermal averaging over DFT-computed paramagnetic spin states can reproduce observed temperature-dependent shifts in ^13^C pNMR spectra of MOFs with Cu(ii) paddlewheel dimers.

## Graphical abstract

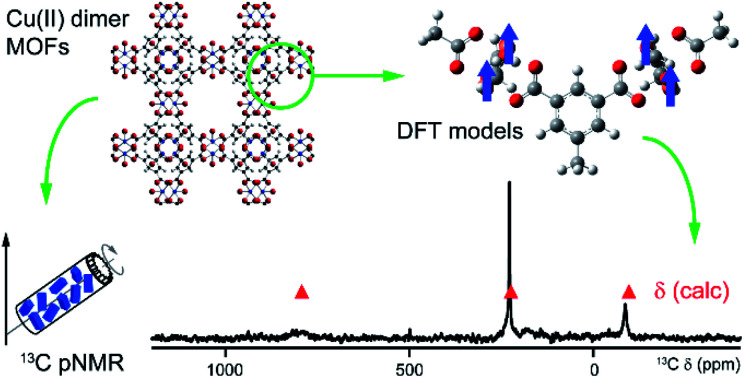

## Introduction

Solid-state Nuclear Magnetic Resonance (NMR) spectroscopy provides a very sensitive probe of the local, atomic-scale environment of the nuclear spins present in a sample without any requirement for long-range order.^1–3^ The sensitivity of the NMR parameters to small changes in geometry has resulted in this method being used to probe weak bonding interactions, host–guest interactions and both static and dynamic disorder. The NMR parameters can also depend on temperature; for diamagnetic materials, the chemical shifts are only weakly temperature dependent (in the absence of significant dynamics in the system), whereas for paramagnetic materials the changes can be much greater, and the control and monitoring of temperature during an NMR experiment is crucial in obtaining consistent spectra and in interpreting these correctly.^4,5^ Measuring the temperature dependence of paramagnetic NMR (or “pNMR”) shifts can provide additional information about the spin physics and electronic structure, ultimately aiding the assignment of resonances for materials where the shifts are often unusual or difficult to predict in advance.

**Fig. 1:**
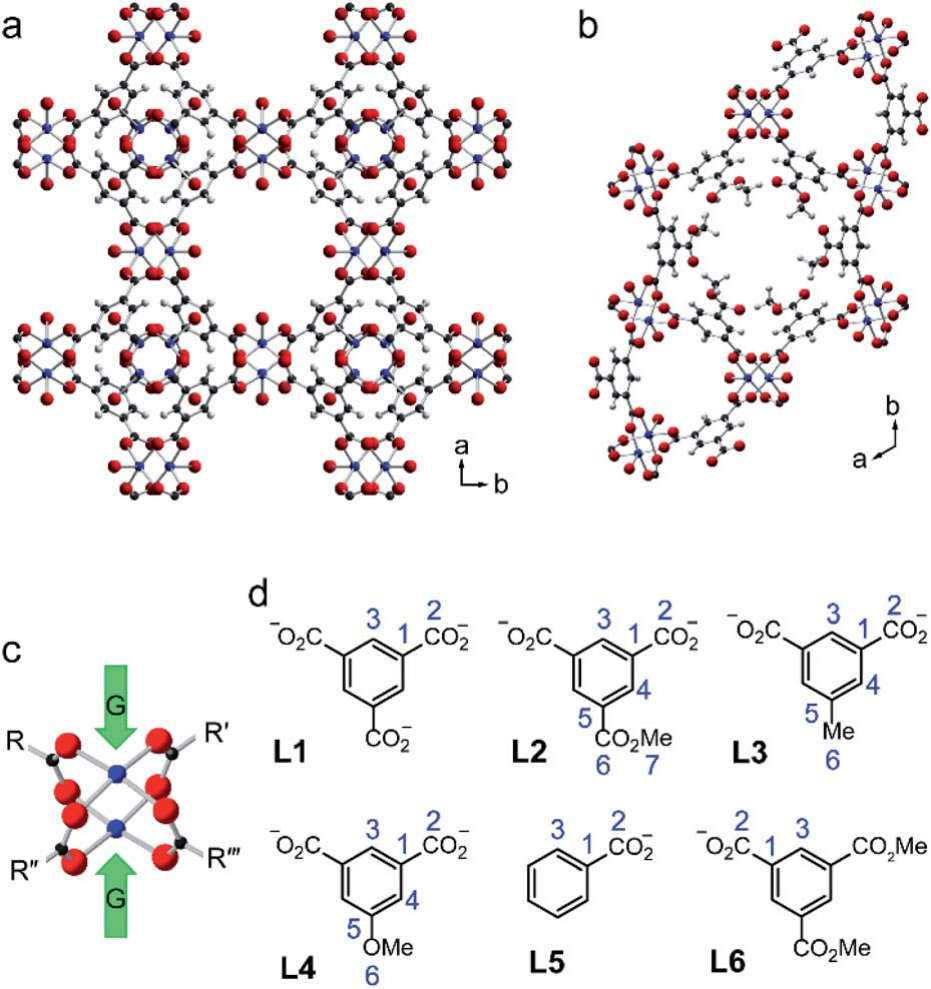
Crystal structures of (a) HKUST-1 ^13^ and (b) STAM-1.^14^ (c) Structure of a copper paddlewheel dimer: green arrows indicate the axial guest binding sites (G). (d) The linkers and numbering schemes used in this work for HKUST-1 (L1), STAM-1 (L2), STAM-17-Me (L3) and STAM-17-OMe (L4). Additionally, numbering schemes are shown for the benzoate (L5) and dimethyl trimesate (L6) ligands, with only C species analogous to those in the MOFs numbered. The colouring scheme in parts (a)–(c) is Cu = blue, O = red, C = black, H = pale grey.

Metal–organic frameworks (MOFs) are well-known microporous materials, containing pores with diameters typically between 5 and 20 Å. These frameworks are charge neutral and composed of “nodes” of one or more metal ions coordinated by polytypic organic linkers, often based on benzene rings, such as benzene-1,3,5-tricarboxylate (BTC) and benzene-1,4-dicarboxylate (BDC). The versatile chemistry of MOFs, with many possible combinations of nodes and linkers, leads to a range of applications in fields as diverse as gas storage, catalysis and drug delivery.^6–8^ Solid-state NMR spectroscopy is frequently used to study MOFs, enabling the binding of guest molecules, the dynamics of guest species within the pores, and any structural changes that can result from these to be explored.^9–12^ MOFs that contain paramagnetic metal centres pose a particular challenge for NMR spectroscopy, but such materials often have interesting and useful physical and chemical properties that can be exploited. HKUST-1 is a MOF containing Cu(ii) paddlewheel dimers linked by BTC (Fig. 1A), with guest molecules able to bind to the free coordination sites (Fig. 1C).^13^ Modification of the linker (Fig. 1D) leads to STAM-1 (linker L2), which contains both hydrophobic and hydrophilic pores (Fig. 1B)^14^ and the isoreticular STAM-17 series, (with L3, L4 or a range of linkers with larger alkyloxy groups).^15^ The STAM series of MOFs demonstrate an unusually high hydrolytic stability compared to the chemically similar but topologically different HKUST-1. This was shown to be related to a significant bulk structural rearrangement upon the interaction with water, likened to the dissipation of energy seen in the crumple zone of a car (and leading to the term “crumple-zone MOFs” for these materials).^15,16^ Dawson *et al.* demonstrated that fast (40–60 kHz) MAS and variable offset experiments were necessary to acquire undistorted, high-resolution ^13^C NMR spectra of these MOFs, which display signals over a shift range of −100 to +1000 ppm.^17^ However, isotopic labelling of specific C sites on the linker was required to unambiguously assign the resonances in HKUST-1 and STAM-1, which demonstrated that the most shifted signal did not result from the C closest to the Cu atoms, as might have been expected.^17^ For a mononuclear Cu(ii) complex, the ^13^C isotropic shift (*δ*_iso_) is expected to be linearly related to 1/*T*, with the extrapolation to *T* = 0 providing an estimate of the diamagnetic orbital shift (*δ*_iso(orb)_), as shown in our previous work on Cu(ii) oximes.^18,19^ However, for a Cu(ii) benzoate dimer, the dependence of the experimental ^13^C *δ*_iso_ on 1/*T* was clearly non linear, leading to incorrect *δ*_iso(orb)_ shift predictions.^16,20^ In this work, we provide insight into the origin of the unusual temperature dependencies using a combination of NMR measurements and computational modelling.

For many materials, Density Functional Theory (DFT) provides a complementary approach to experimental NMR spectroscopy, helping to facilitate the interpretation, assignment and prediction of spectra.^21,22^ While now widely applied for diamagnetic materials (in an approach that is often referred to as NMR crystallography), such calculations are considerably more challenging for paramagnetic solids, although arguably of greater benefit owing to the significant and unusual shifts observed. In the solid state, periodic approaches for computing the NMR parameters (*e.g.*, the Gauge-Including Projector Augmented Wave (GIPAW) method)^23^ have proven extremely useful. However, their extension to paramagnetic materials is still in its infancy.^4,24^ For isolated molecules with paramagnetic centres, a number of theoretical approaches for calculating NMR isotropic shifts have been developed,^25–28^ which contain temperature-dependent expressions related to the magnetic susceptibility of decoupled magnetic moments. However, in extended solids the magnetic moments of the individual paramagnetic sites may communicate and couple differently, complicating the modelling using a periodic approach.^29^ Moreover, the paramagnetic MOFs of interest in this work, HKUST-1 and the STAM family, have the additional complication of interacting spins on the paramagnetic centres in the paddlewheel dimer (and the possibility of a thermal equilibrium of differently (ferro- or antiferromagnetically) coupled states), in addition to potential couplings between dimers. In recent work we have shown that the experimental ^13^C *δ*_iso_ of a solid containing molecular Cu(ii) paddlewheel dimers can be reproduced computationally assuming a thermal equilibrium between a diamagnetic ground state (with antiferromagnetic spin coupling on the Cu dimer) and an excited triplet state (with ferromagnetically coupled spins for a single molecule).^20^ In this work we will explore how pNMR parameters calculated for a series of increasingly complex isolated molecules can be used to provide insight into those measured experimentally for extended Cu(ii) paddlewheel MOFs. We consider a set of models containing increasing numbers (and different arrangements) of paddlewheel dimer units and demonstrate that explicit consideration of the population of different spin configurations can explain both the experimentally observed shifts and the unusual temperature dependence of these in this interesting set of MOFs. The insights from this study will expand the applicability of NMR spectroscopy as a noninvasive tool to obtain information on the electronic and geometric structures of paramagnetic MOFs and related materials, for instance in the context of probing guest binding at the paramagnetic centres.

## Methods

### Synthesis

HKUST-1 and STAM-1 were prepared as described in ref. 17. STAM-17-Me and STAM-17-OMe were prepared as described in ref. 16.

### Solid-state NMR spectroscopy

^13^C magic angle spinning (MAS) NMR spectra were recorded using Bruker Avance III spectrometers with 14.1 or 20.0 T wide-bore superconducting magnets (^13^C Larmor frequencies of 150.9 and 213.8 MHz, respectively). Experimental details for the variable-temperature (VT) ^13^C NMR spectra of as-made HKUST-1 and STAM-1 (recorded at 20.0 T) can be found in ref. 30, and those for STAM-17-Me and STAM-17-OMe (recorded at 14.1 T) are given in ref. 16. The VT ^13^C NMR spectra of dehydrated (activated) HKUST-1 were recorded at 20.0 T. Activated HKUST-1 was packed into a 1.3 mm ZrO_2_ rotor and rotated at a MAS rate of 60 kHz. The data were obtained as two sub-spectra with different frequency offsets to provide higher sensitivity for the C1 and C2 signals (see Fig. 1D for numbering scheme). Spectra were acquired using a rotor-synchronised spin-echo pulse sequence, with *τ* = 16.7 μs and a recycle interval of 20 ms. Signal averaging was carried out for 40 960 transients (C2 and C3 subspectrum) or 131 072 transients (C1 subspectrum). The temperature was monitored by an external thermocouple within the probe, and varied between 250 and 323 K. The temperature was allowed to equilibrate until a stability of ±0.1 K was achieved for a period of five minutes. The temperature was calibrated externally using KBr to account for the ∼20 K frictional heating arising from the rapid MAS. Additional calibration to account for the absolute sample temperature was not carried out and, therefore, there may be a small error in the temperature reported.‡‡When comparing the spectra recorded at 20.0 T with in-house data, recorded using a more carefully calibrated temperature (using the phase transition temperature of DABCO at 351.1 K as a reference for the absolute temperature), we observed a maximum shift difference of ∼1 ppm for the C2 resonances in spectra of the same MOFs recorded at nominally the same temperature but at different fields. Shifts are reported in ppm relative to TMS using l-alanine (*δ*(CH_3_) = 20.5 ppm) as a secondary solid reference.

### Calculations

**Fig. 2:**
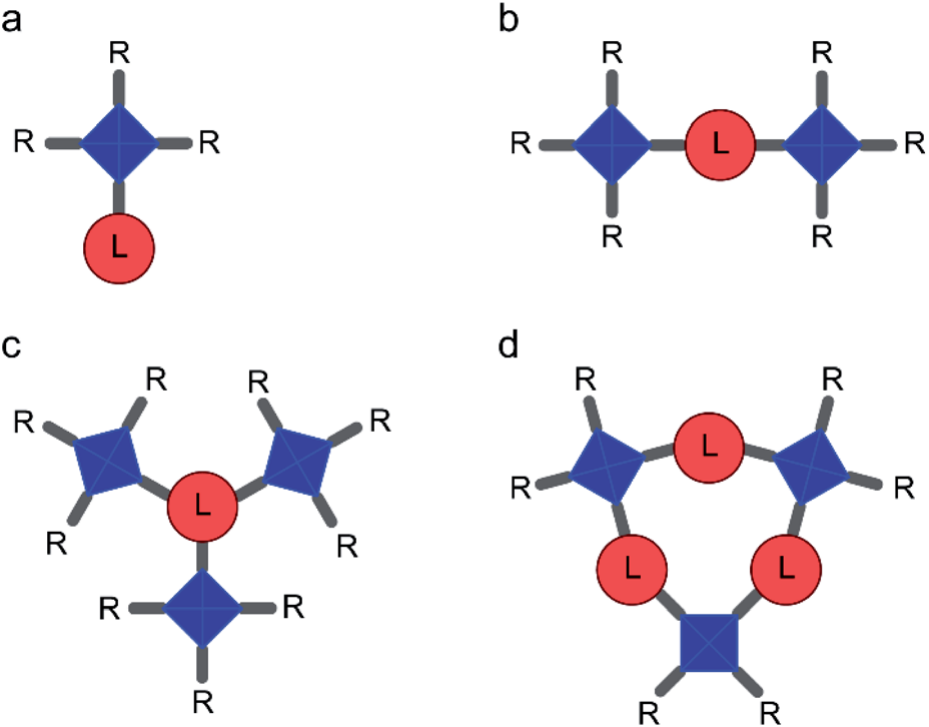
Schematic representation of models containing (a) one copper paddlewheel dimer (“mono-dimer” models, M) (b) two dimers (“di-dimer” models, D), (c) three dimers arranged radially around one linker (“radial tri-dimer” models, Ra) and (d) three dimers arranged in a ring (“ring tri-dimer” models, Ri). Copper dimers (Fig. 1C) are shown in blue and linkers (Fig. 1D) in red. Pendant R groups are either acetate or additional linkers, as detailed in Table 1.

The structures of all models considered were optimised using GAUSSIAN 09 ^31^ at the PBE0-D3 level or using GFN2-xTB.^32–37^ In conjunction with PBE0-D3, an augmented Wachters basis set^38,39^ was used for Cu (8s7p4d) with the full contraction scheme 62111111/3311111/3111, while the 6-31G* basis set was used for the ligands (this combination of basis sets is labelled here as AE1). For model D1 (see Fig. 2 and Table 1), structural optimisation was carried out separately for each possible spin state (see below and ESI†) using unrestricted Kohn–Sham wavefunctions with a broken-symmetry (KS-BS) solution for the open-shell spin state (*e.g.*, expectation values of the *Ŝ*^2^ operator of 1.985, 2.997 and 6.010 for the singlet, triplet and quintet states, respectively in model D1). As shown in the ESI (Table S11)† for di-dimer models, the optimised geometries for the different spin states are very similar, and so the structure of the high-spin state was used in pNMR calculations for all spin states. For GFN2-xTB calculations, the basis set was an augmented minimal basis. At this level, structures were optimised for the high-spin state only. The character of each stationary point was verified by the computation of the harmonic vibrational frequencies. The frequencies were also used to obtain thermodynamic corrections to relative enthalpies and free energies.

**Table 1:** Summary of model structures. For the notation used for the linkers, L, refer to Fig. 1D and for the geometries denoted M, D, Ra and Ri, see Fig. 2

Model	L	R groups	Axial guest	Formula
M1	L5	3 × L5	No	Cu_2_(L5)_4_
M2	L6	3 × L6	No	Cu_2_(L6)_4_
M3	L5	L5 + 2 × OAc	No	Cu_2_(L5)_2_(OAc)_2_
M4	L5	3 × OAc	No	Cu_2_(L5)(OAc)_3_
D1	L3	6 × OAc	No	Cu_4_(L3)(OAc)_6_
D2	L4	6 × OAc	No	Cu_4_(L4)(OAc)_6_
D3	L2	6 × OAc	No	Cu_4_(L2)(OAc)_6_
D4	L3	6 × OAc	4 × H_2_O	Cu_4_(L3)(OAc)_6_·4(H_2_O)
D5	L4	6 × OAc	4 × H_2_O	Cu_4_(L4)(OAc)_6_·4(H_2_O)
D6	L2	6 × OAc	4 × H_2_O	Cu_4_(L2)(OAc)_6_·4(H_2_O)
Ra1	L1	9 × OAc	No	Cu_6_(L1)(OAc)_9_
Ra2	L1	9 × OAc	6 × H_2_O	Cu_6_(L1)(OAc)_9_·6(H_2_O)
Ri1	L3	6 × OAc	No	Cu_6_(L3)(OAc)_6_
Ri2	L4	6 × OAc	No	Cu_6_(L4)(OAc)_6_
Ri3	L2	6 × OAc	No	Cu_6_(L2)(OAc)_6_
Ri4	L3	6 × OAc	6 × H_2_O	Cu_6_(L3)(OAc)_6_·6(H_2_O)
Ri5	L4	6 × OAc	6 × H_2_O	Cu_6_(L4)(OAc)_6_·6(H_2_O)
Ri6	L2	6 × OAc	6 × H_2_O	Cu_6_(L2)(OAc)_6_·6(H_2_O)

NMR and EPR parameters were computed for the optimised structures at the CAM-B3LYP^40^ level, employing a 9s7p4d (621111111/3311111/3111) basis set on Cu, which was constructed specifically for calculating accurate hyperfine coupling constants,^41^ and IGLO-basis II^42^ on the ligands (this combination of basis sets is labelled II). Orbital shieldings, *σ*_iso(orb)_, were computed using the GIAO (gauge-including atomic orbitals) implementation in Gaussian 09 for all spin states in a structure optimised for the high-spin state. The hyperfine coupling and ***g*** tensors were computed for all spin states (with the exception of the singlet) at the CAM-B3LYP/II level using ORCA.^43^ Energy differences, Δ*E*, with respect to the lowest energy configurations were evaluated at the CAM-B3LYP/II level using the broken-symmetry (BS) approach of Noodleman.^44–46^ The zero-field splitting (ZFS) parameters were calculated using the coupled-perturbed method of Neese^47^ at the BLYP/II level (for technical reasons only a non-hybrid functional could be used here). The structure and ^13^C *σ*_iso(orb)_ of the reference compound, TMS, was computed at the same level of theory.

Paramagnetic shieldings were calculated using the approach of Hrobárik and Kaupp^48^ with

1 *σ*_iso_ = *σ*_iso(orb)_ − [*S*(*S* + 1)*β*_e_/3*k*(*T* − *Θ*)*g*_N_*β*_N_][*g*_e_*A*_FC_ + *g*_e_*A*_PC_ + Δ*g*_iso_*A*_FC_ + (1/3)Tr(Δ***g***_aniso_·***A***_dip_)],

where *A*_FC_ and ***A***_dip_ are the isotropic Fermi contact and anisotropic traceless spin-dipolar contributions to the ***A*** tensor, respectively, *A*_PC_ is the isotropic pseudocontact (PC) term arising from spin–orbit corrections to the *A* tensor, Δ*g*_iso_ = 1/3 Tr(***g***) − *g*_e_ and Δ***g***_aniso_ is the anisotropy of the ***g*** tensor. *Θ* is the Weiss constant (which is set to zero unless otherwise stated). Magnetic shieldings were Boltzmann averaged over all spin states, i, with energy gaps all scaled by a single scaling factor, *s*, for the relative energies as follows

2 *σ*_iso_ = ∑*f*_i_*σ*_iso,i_,

3 *f*_i_ = *g*_i_ exp(−*s*Δ*E*_i_/*RT*)/Σ*g*_i_ exp(−*s*Δ*E*_i_/*RT*),

where *g*_i_ is the degeneracy of spin state i and Δ*E*_i_ is its energy relative to the ground state. For a single dimer, this energy corresponds to Δ*E*_ST_, *i.e.*, the energy difference between singlet and triplet states, taken as the exchange coupling constant *J*_12_ in the spin-coupling Hamiltonian *Ĥ*_S_ for two spin state operators (*Ŝ*_1_ and *Ŝ*_2_).^49^ (Note that, as shown below and in the ESI,† the energy gap between singlet and triplet states of a given dimer is effectively independent of the configuration of any other dimers within the model, such that we use the notation Δ*E*_ST_ to indicate the intradimer coupling for all models, regardless of the overall spin of the model). This corresponds to the expression used in our previous work for a urea-loaded copper benzoate dimer (with *s* = 1), where the combination of functionals and basis sets has been previously validated.^20^ Full expressions for models with two and three dimers (with different choices of combinations of electronic states) are given in the relevant sections of the ESI.†

## Results and discussion

### Models with a single copper paddlewheel dimer

The simplest models for HKUST-1 are based on an isolated paddlewheel dimer unit, as shown in Fig. 2 and Table 1. In the basic model (M1), four benzoate ligands are attached to the dimer, leaving two vacant coordination sites that could, in principle, be occupied by guest molecules. For HKUST-1, it is possible to consider an “activated MOF”, *i.e.*, where these coordination sites remain vacant, which can be achieved experimentally by heating under vacuum to remove guest molecules.^50^ It should be noted that this is not possible for molecular benzoates (such as that studied in ref. 20), which would decompose or coordinate with other donors, or for the STAM-based crumple zone MOFs which undergo a structural phase transition^15,16^ (not considered in detail here). Model M1 can be adapted, as shown in Fig. 2 and Table 1, to more closely resemble HKUST-1 (as in M2 where ester functional groups are added in *meta* positions on each ring) or with the aim of increasing computational efficiency (as in M3 and M4 where two or three L5 are replaced by acetate, respectively). The structures of these four models, optimised using GFN2-xTB are shown in Fig. S2.†

**Fig. 3:**
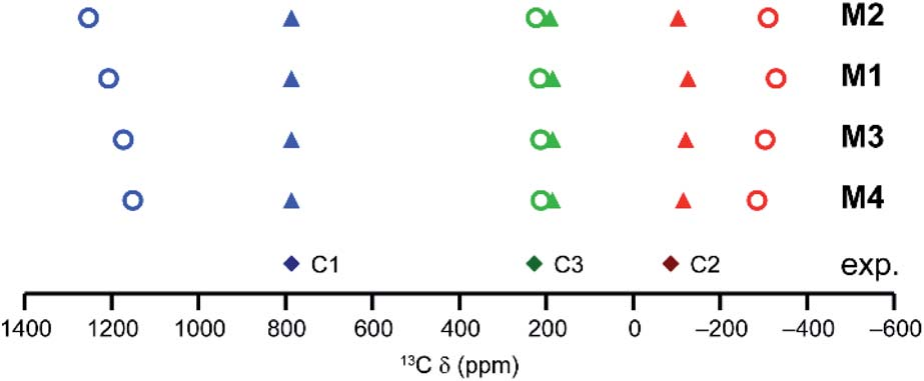
As-calculated (circles) and scaled (triangles) ^13^C isotropic shifts for models M1, M2, M3 and M4, at 298.2 K (CAM-B3LYP/II//GFN-xTB). The energy scaling factors are *s* = 1.86 (M1), 1.94 (M2), 1.78 (M3) and 1.73 (M4). The experimental data for activated HKUST-1 (298 K) are shown as diamonds.

For a single paddlewheel dimer, three spin configurations are possible; two singlets and a triplet state (shown in Section S2 of the ESI† for model M4). The triplet state is well represented by a single configuration, but the singlet state is a linear combination of two configurations. The expression used to determine the pNMR shifts for the single dimer models is given in Section S2 of the ESI.†Fig. 3 shows calculated ^13^C shifts for models M1–M4, along with the experimental shifts of activated HKUST-1 (labelled using the scheme in Fig. 1D). In each case, the paramagnetic shifts of C2 and C1 are overestimated (too negative and too positive, respectively) when compared with experiment (open circles in Fig. 3). The addition of the ester groups (M2) more than doubles the CPU time (see Table S1†), with little, if any, improvement in the agreement between experimental and calculated shifts. Simplifying the dimer (M4) significantly speeds up the calculations (by ∼1.5 and ∼3 times for geometry optimisation and pNMR calculations respectively, Table S1†). The calculated shifts of these models appear to be slightly closer to experiment, but the disagreement is still significant. The changes seen for C1 and C2 mainly stem from the changes in triplet shielding values, which involve a large contribution from Fermi-contact shifts and become less pronounced (smaller in absolute value) as Ph groups are replaced with Me. The modifications of the initial dimer model result in a slight elongation of the distance between the two Cu atoms (of up to 0.02 Å), and a small change (between −3 and +11 cm^−1^) in Δ*E*_ST_ (see Table S2†).

Although there is reasonable qualitative agreement between the calculated and experimental shifts, none of the models produce shifts in good quantitative agreement. For example, for C1 calculated isotropic shifts are between 1100 and 1250 ppm, compared to the experimental shift at ∼800 ppm. C1 signals above 1000 ppm were seen in previous work for a urea-loaded copper benzoate dimer, and in this case the computational and experimental shifts were in much better agreement.^20^ This perhaps suggests that models containing a single dimer work well for molecular solids, where the Cu paddlewheel units are so spatially remote that no interaction between them is expected. However, for MOFs, the dimer units are much closer and can interact *via* the aromatic organic linkers that joint them, suggesting more sophisticated models and a proper theoretical description of the magnetic coupling between dimers (see later) will be needed. This is supported by comparing the difference in the experimental ^13^C shifts for the aromatic CH species in hydrated HKUST-1 and hydrated STAM-1; signals are seen at *δ*_iso_ ≈ 227 ppm for the CH groups between two dimers in both MOFs, but the C4 signal in STAM-1 (an aromatic CH that is adjacent to one dimer only) has *δ*_iso_ = 178 ppm.

One possible additional reason for the poorer agreement between theory and experiment is the need to include macroscopic effects on the magnetic susceptibility (which enters the pNMR expression *via* a temperature-dependent pre-factor containing the Weiss constant *Θ* as shown in eqn (1)).^24,51,52^ A large negative *Θ* would decrease the pNMR contributions at a given temperature. While it would be possible to improve the agreement between calculated and observed shifts in Fig. 3 through empirical fitting of the Weiss constant (see Table S3† for resulting values for model M4 with *Θ* = −170 K), this was not employed routinely here for two reasons; firstly, the Weiss constants determined experimentally for MOFs of this type appear to be reasonably small (*e.g.*, 4.7 K for HKUST-1 loaded with pyridine^53^) and secondly, the temperature dependence of the pNMR shifts is more complicated than the expression given in eqn (1), as it also depends on the thermal population of the paramagnetic states (see eqn (2)). A different Weiss constant would have to be fitted for each temperature separately, which is both unphysical and impractical. The dependence of the pNMR shifts on the population of the paramagnetic states (*i.e.*, the triplet state for models with a single dimer), also leads to a possible source of error in the calculations, with an over- or underestimation resulting from an inaccurate determination of the energy gaps (*i.e.*, Δ*E*_ST_). While this energy was used as calculated in our previous work on the urea-loaded benzoate dimer (*i.e.*, *s* = 1),^20^ this may not be suitable when attempting to model extended MOF systems, where communication between dimer units may raise the energy of the paramagnetic states, thereby reducing their contribution to the overall pNMR shifts. The Δ*E*_ST_ energy gap can be simply scaled, with the scaling factor *s* (eqn (3)) determined by minimising the mean absolute deviation (MAD) between experimental and theoretical shifts for the three ^13^C resonances in HKUST-1. This results in values of *s* between 1.7 and 2.0 for the four single dimer models and much improved agreement between experiment and theory, as shown in Fig. 3 (triangles). This indicates that the high spin states are populated to a lesser extent in the MOF than in the copper benzoates studied previously. Table S2† gives values of Δ*E*_ST_ for the models M1 to M4 with different scaling factors, and Table S4† gives values calculated for each model with their respective values of *s* (ranging between 1.73 and 1.94).

In principle, it would be possible to use experimental information such as exchange couplings from EPR measurements (see footnote *a* in Table S2†) instead of calculated and scaled Δ*E*_ST_ values, but this is undesirable for a number of reasons. These include loss of predictive power if information from experiment is vital for the calculations (assuming our empirical scaling factors are transferable between systems), and practicability, as separate such measurements would then be required for each and every system under scrutiny, *e.g.*, upon loading with different guests.

### Models with two copper paddlewheel dimers

The use of models with more than one copper dimer offers a potentially more accurate description of the long-range MOF structure, and the option to explore chemical differences between the Cu-based MOFs more easily. Models D1, D2 and D3, shown in Fig. 2B and Table 1 are based on L2, L3 or L4 (to model STAM-1, STAM-17-Me or STAM-17-OMe, respectively^14–16^), with two simplified paddlewheel dimers (R = acetate) attached. All three can also serve as potential models for HKUST-1, but more sophisticated models, where the linker is trimesate (L1) and the third substituent is another paddlewheel dimer (model Ra1, see later) can also be used. As noted in the methodology section above, optimised geometries of model D1 are very similar for all spin states at the PBE0-D3/AE1 level (see Table S11†). For the results described in this work, structures were optimised for the high spin state only using the GFN2-xTB semi-empirical method. From the mono-dimer models, it is clear that the pNMR shifts depend strongly on the energy gaps between the spin states, but that any inability of DFT to predict these accurately might be alleviated through the use of a scaling parameter. For the di-dimer models, where a greater number of spin states are possible, a single scaling factor, *s*, was applied to all energy differences (see Section S3 of the ESI† for more detail). When attempting to fit *s* using the pNMR parameters obtained at a single temperature, a spread of values was obtained depending on the temperature chosen. This approach is both unphysical and not particularly helpful for understanding or, perhaps more importantly, predicting experimental results. Therefore, *s* was determined for each model by obtaining the closest match between experimental and computed shifts for all temperatures simultaneously. In the following discussion, results are shown after this scaling has been applied. The spin states used (*i.e.*, i in eqn (2) and (3)) can consist of more than one BS spin configuration. Information on how these were selected and the equations used to calculate the shieldings is given in Section S3 of the ESI† (note the combination in Fig. S5† and the resulting eqn S8† was adopted for the results shown below).

#### Models for activated MOFs

**Fig. 4:**
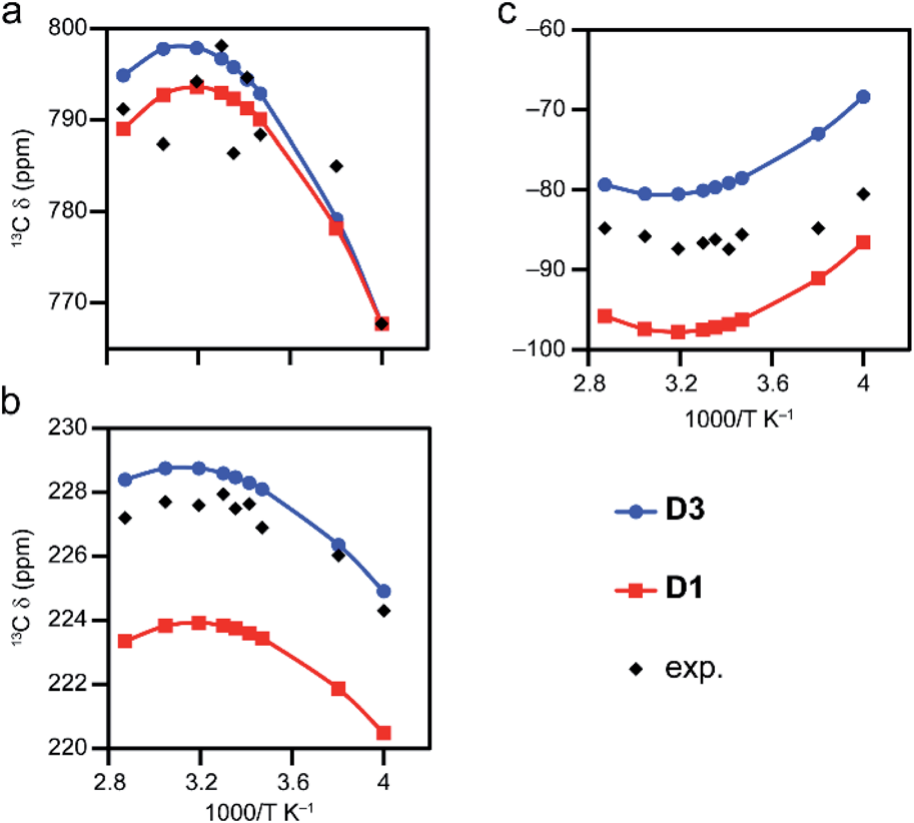
Temperature dependence of experimental and calculated ^13^C shifts for (a) C1, (b) C3 and (c) C2 in activated HKUST-1, using models D1 and D3, with Δ*E*_ST_ of ∼219 cm^−1^, scaled by a factor of *s* = 1.305 and 1.330 (CAM-B3LYP/II//GFN2-xTB), respectively, employing eqn S8.†

As discussed above, activation of STAM-17 and STAM-1 results in a phase transition that introduces additional structural and spectral complexity, which is not considered further here.^15,16^ For HKUST-1, the framework geometry is similar after activation.^54^ Shifts predicted for model D1 (*s* = 1.305) in the temperature range 250 to 300 K are shown in Fig. 4 (plotted against 1/*T*) for C sites that match the three sites in HKUST-1. Note that the computed shifts for chemically related C sites (*i.e.*, those that are equivalent in the parent linkers in Fig. 1D) in the model have been averaged. Experimental shifts for activated HKUST-1 are also shown. The non-linear dependence of the shifts with 1/*T* seen experimentally is well reproduced by computation (with most shifts reproduced within 10 ppm). While model D1 works well for HKUST-1, model D3 might be a better representation of the MOF structure as it has three carboxylate-based substituents. This substitution does not affect Δ*E*_ST_ (∼219 cm^−1^) but does lead to a slightly altered scaling factor (1.330 *cf.* 1.305), and differences in the scaled energies of 5 to 10 cm^−1^ (see Tables S5 and S8†). The agreement with experiment improves significantly for C2 and C3 on changing from Me to CO_2_Me, and the computation reproduces well the temperature dependence of the shifts, which is now rationalised in terms of the temperature dependences of both the pNMR shifts of the paramagnetic states (eqn (1)) and the Boltzmann equilibria (eqn (3)).

#### Models for hydrated MOFs

Models for hydrated MOFs can be generated by the addition of four water molecules to the open metal sites in models D1–D3, giving models D4–D6 (see Fig. 1, 2 and Table 1). Optimised geometries are shown in Fig. S9.† Although the orientation of the guests can break the symmetry of the model for the simplest case (model D4), a simplified form of eqn S8, given in eqn S15,† was employed to save computational time when predicting the isotropic shifts of STAM-17-Me by assuming the equivalence of BS configurations that are mirror images in pseudo-*C*_s_ symmetry. Using experimental ^13^C shifts (note only a single measurement was made for C1, at 298 K(ref. 16)) a scaling factor of 1.374 was obtained. Good agreement can be seen between experiment and computation, as shown in Tables S13 and S14.† The effect of ZFS on the ^13^C shift is smaller than 2 ppm and can be neglected (see Tables S15 and S16†). Although the model for STAM-17-OMe (D5) is less symmetric than for STAM-17-Me, eqn S15† also works well (with *s* = 1.306), giving both shifts and their temperature dependence in good agreement with experiment (with a MAD below 6 ppm at each temperature, as shown in Tables S17 and S18†).

**Fig. 5:**
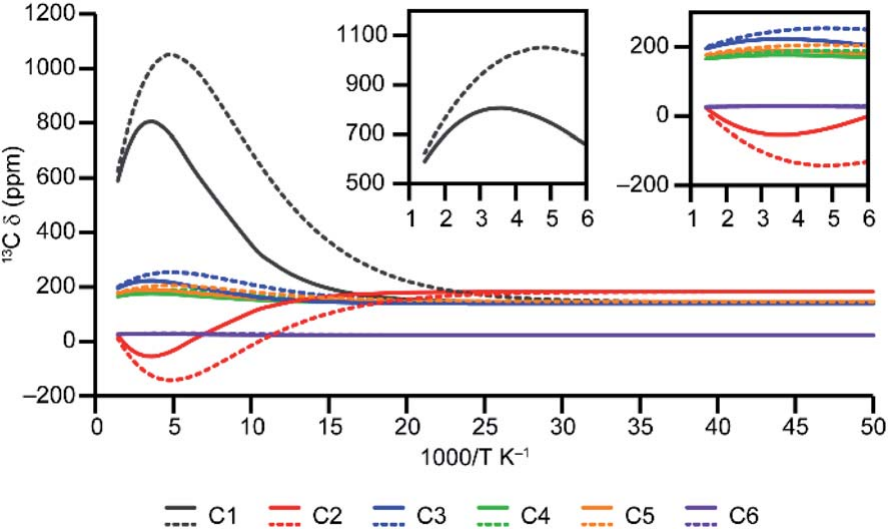
Plots of ^13^C *δ*_iso_ for model D4 as a function of inverse temperature, calculated using *s* = 1.374 (from fitting of the experimental data of STAM-17-Me),^16^ shown by solid lines and as-calculated (*i.e.*, with *s* = 1) shown by dotted lines. The insets show the data in the range of 1000/*T* below 6 K^−1^ where 4–2.9 K^−1^ is the temperature range of 250–348 K (CAM-B3LYP/II//GFN2-xTB).

**Fig. 6:**
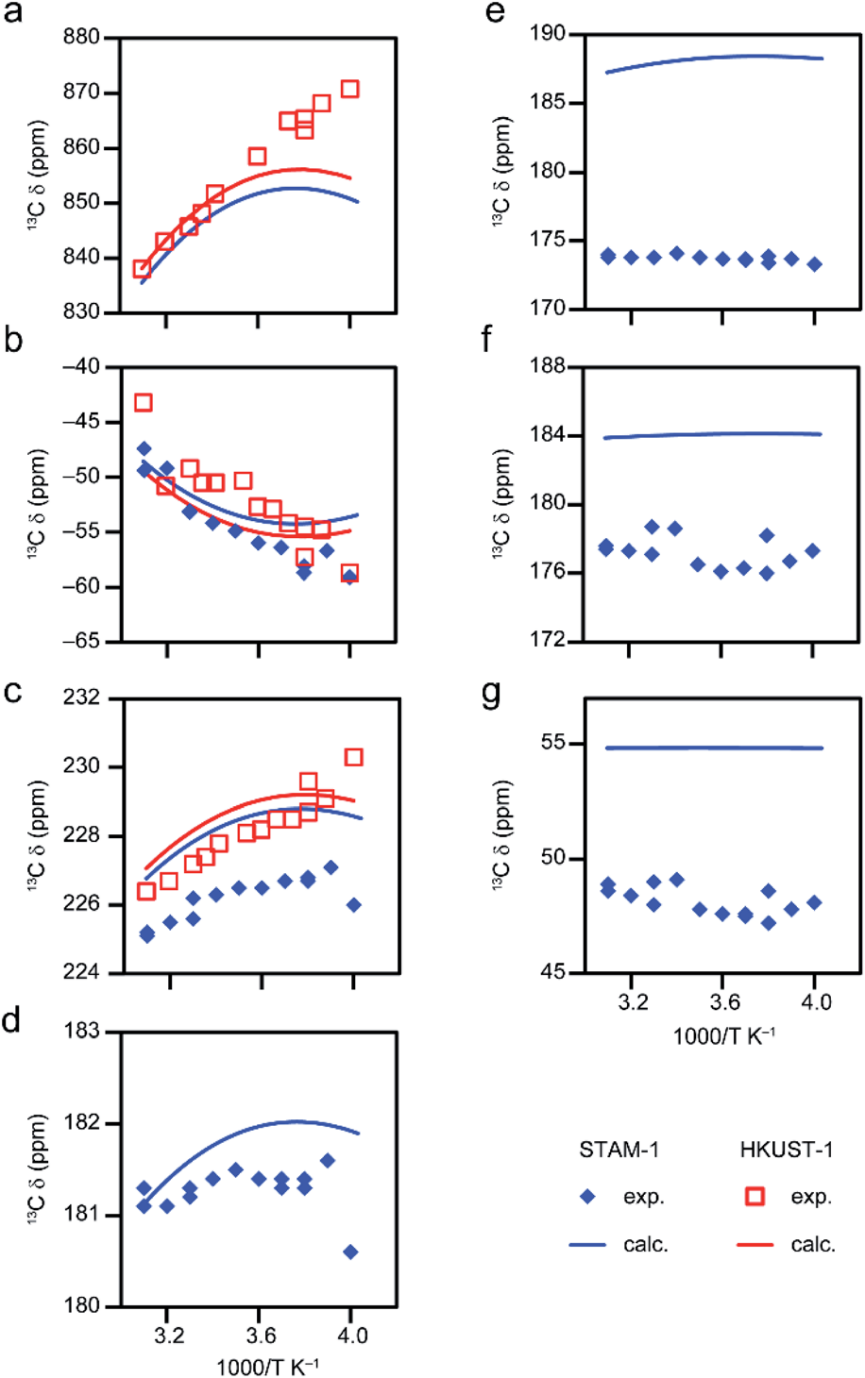
Temperature dependence of ^13^C shifts of hydrated STAM-1 and HKUST-1 from experiment^14,30^ and computation for model D6 with *s* = 1.295 and 1.288, respectively for STAM-1 and HKUST-1 for Δ*E*_ST_ ≈ 187 cm^−1^ (CAM-B3LYP/II//GFN2-xTB), using eqn S15.† Results are presented for (a) C1 in both MOFs (no experimental data for STAM-1 owing to the very broad resonance leading to unfeasibly long experimental times), (b) C2 in both MOFs, (c) C3 in both MOFs, (d) C4 in STAM-1, (e) C5 in STAM-1, (f) C6 in STAM-1 and (g) C7 in STAM-1 (See Fig. 1D for numbering schemes).

The non linear relationship between the ^13^C *δ*_iso_ and 1/*T* for the Cu(ii)-based MOFs is in contrast to the behaviour seen for systems with a single spin centre (such as the complexes in ref. 18 and 19), and is a consequence of the temperature-dependent population of the paramagnetic states in thermal equilibrium. Scaling of the DFT-calculated Δ*E*_ST_ is required to ensure that this population is accurately reproduced. The effect of scaling on the computed shifts is illustrated in Fig. 5 for model D4 over the temperature range 20 to 698 K. The shifts initially show maxima or minima at 1000/*T* ≈ 5 K^−1^ (*i.e.*, *T* = 200 K). Upon scaling Δ*E*_ST_, the maximum and minimum shifts become less extreme and occur at *T* ≈ 250 K. It will depend on the system under study, therefore, whether the maxima/minima fall within a temperature range that is experimentally accessible under MAS (250–350 K). For activated HKUST-1, these are observed in experimental measurements (Fig. 4), but fall outside of the accessible temperature range for hydrated MOFs, such as hydrated HKUST-1 (as shown in Fig. 6).

Fig. 6 shows ^13^C isotropic shifts for model D6, computed using eqn S15† (see Tables S19 and S20†) with *s* = 1.295 (determined using the experimental shifts for STAM-1).^30^ The calculated shifts match experiment reasonably well for all carbon sites (see Fig. 1D for the numbering scheme). A version of this model without coordinated water molecules (D3) was used to compute shifts for activated HKUST-1 in the previous section, and so the computed shifts for this hydrated model can also be compared with the experimental values for hydrated HKUST-1 (also shown in Fig. 6). If the scaling factor for the computed shifts is determined not by fitting to experiments for STAM-1 but only to those for HKUST-1 (which has just three C sites), a small decrease (to 1.288) is observed, leading to the slightly different calculated shifts shown in Fig. 6.

Above 273 K (below 3.66 K^−1^) where chemically similar C environments exist for the two MOFs (*i.e.*, C1, C2 and C3), the experimental shifts are very similar,^17,30^ validating the use of the same structural model for the two. However, below 273 K (above 3.66 K^−1^), these resonances seem to shift in different directions in Fig. 6B and C. For example, C2 (Fig. 6B) becomes more shielded for HKUST-1 while the shift of the same site in STAM-1 appears to vary little with temperature in this range. This flattening of the curve is captured in the computed shifts (see Table S20† for numerical data). It is unclear whether the observed deviations between the two samples at low temperature (up to ∼5 ppm in Fig. 6C) arise from (i) experimental uncertainties (suggested by the scatter of the experimental data), (ii) the structural differences between STAM-1 and HKUST-1 (which have two and three dimers, respectively, attached to each linker – see later discussion of models with three paddlewheel dimers) or (iii) the different behaviour of the extraframework water in the pores. Fully hydrated HKUST-1 is expected to contain free water molecules inside the pores, in addition to those bound to the metal centres, and these may behave differently below 273 K, *i.e.*, on going from liquid-/gas-like to more restricted, crystal-like structures. For hydrated STAM-1, the network of water molecules is more confined, with a larger proportion expected to be bound to the metal sites, and the material has a more complex long-range structure with both hydrophobic and hydrophilic pores and a greater degree of framework flexibility.^14^ Models with a varying number of water molecules were explored computationally to study the effect of any extraframework hydration (see Section S4 of the ESI for more detail†). This shows that the experimental data for STAM-1 would be broadly compatible with models loaded with 4 and 5 water molecules. However, it would be difficult to draw any conclusions from these results regarding the hydration in the experimental sample, where significant dynamics of the water molecules is also likely to occur at room temperature.

#### Substituent effects

**Fig. 7:**
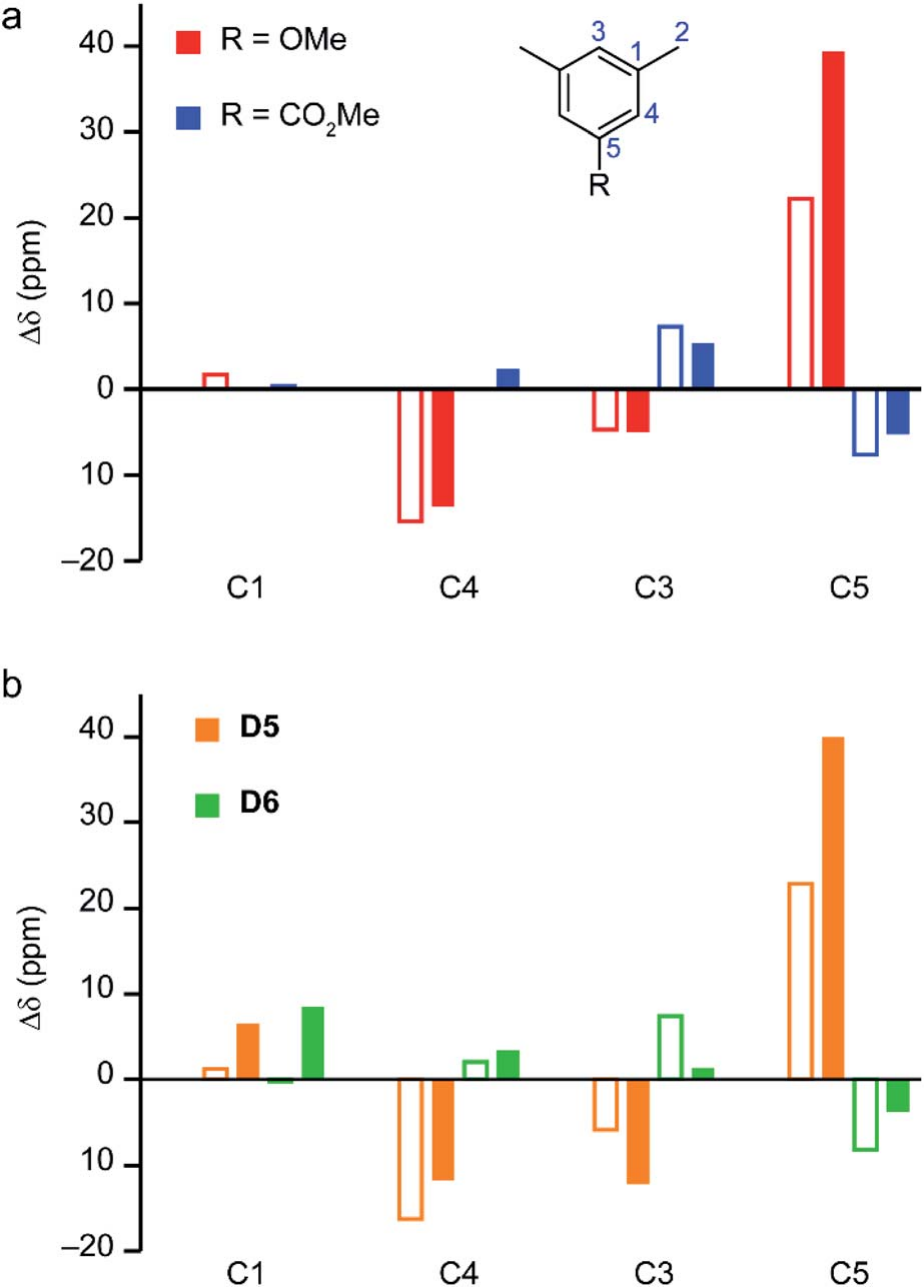
(a) Plot showing the substituent effect on the experimental ^13^C shifts of the MOFs^14,16^ at 298 K (filled bars) and their diamagnetic linker analogues (empty bars, see numbering scheme shown in the inset).^55^ For the diamagnetic molecules with R = OMe (red bars) and CO_2_Me (blue bars), the shifts are plotted relative to mesitylene (R = Me). For the paramagnetic MOFs, shifts are reported relative to STAM-17-Me. (b) The computed changes of the total (filled bars) and orbital (empty bars) shielding for ^13^C for models D5 (orange bars) and D6 (green bars) relative to D4, at 298 K (CAM-B3LYP/II//GFN2-xTB, *s* = 1.340, using eqn S15†).

Fig. S10† shows the temperature dependence of the computed shifts for models D4–D6, where for ease of comparison a single scaling factor (*s* = 1.340) is used. Overall, there is reasonably good agreement between the calculated and experimental temperature dependence of the shifts.^15–17^ Changing the third substituent on the ring results in noticeable changes to the ^13^C shifts for the aromatic resonances, *e.g.*, up to 40 ppm for C5 for the OMe substituent. To probe to what extent these changes arise from paramagnetic contributions, the experimental ^13^C shifts for STAM-17-Me, STAM-17-OMe and STAM-1 can be compared to those seen in solution^55^ for the analogous diamagnetic 1-substituted 3,5-dimethylbenzenes: 1,3,5-trimethylbenzene (R = Me), 1-methoxy-3,5-dimethylbenzene (R = OMe) and methyl 3,5-dimethylbenzoate (R = CO_2_Me). Fig. 7A plots the difference in shifts from the corresponding R = Me compound for those with R = OMe and CO_2_Me. The two sets of compounds (*i.e.*, the MOFs and the molecular analogues) show similar trends upon changing the substituent, with the exception perhaps of C5 in STAM-17-OMe, where the change is larger for the paramagnetic MOF (∼40 ppm *cf.* ∼22 ppm). This suggests that there is relatively little impact of the paramagnetic interaction on the magnitude of the substituent effects. However, the temperature dependence of the ^13^C shifts in diamagnetic organic compounds is very small. For example, for the carbonyl carbon in acetone d*δ*/d*T* = 0.019 ppm K^−1^,^56^ which would lead to changes of ∼1 ppm over the 60 K range considered in Fig. S10.† (Note that this type of temperature effect, arising from changes in the thermally averaged structures, would not be reproduced by calculation). The much larger temperature dependences observed in Fig. S10† are, therefore, a clear indication of a pNMR contribution. The temperature dependences are very similar for all resonances, however, so do not appear when shift differences are considered (as in Fig. 7A).

The calculated pNMR shifts are obtained as a sum of both paramagnetic and orbital shifts. The changes in calculated orbital shifts for hydrated di-dimer models with different substituents (models D5 and D6), shown in Fig. 7B (empty orange and green bars, respectively), are in good agreement with the variation in the experimental shifts for the molecular analogues in Fig. 7A. The effects are consistent with expectations based on the electron donating or withdrawing nature of the substituents (*i.e.*, OMe and CO_2_Me, respectively). The paramagnetic contributions modulate these effects, substantially so for C5, giving variation in the total shifts that agrees better with those seen experimentally for the MOFs.

### Models with three copper paddlewheel dimers

#### Radial models

**Fig. 8:**
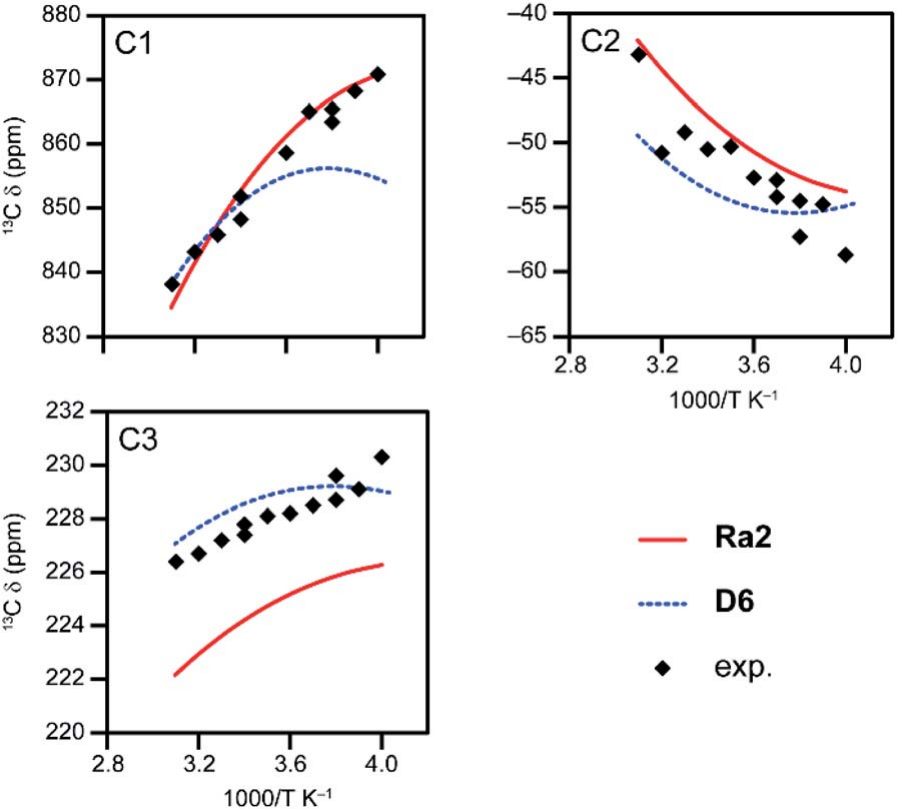
Temperature dependence of calculated ^13^C shifts of model Ra2 using eqn S26† with *s* = 1.141 on an intradimer coupling of ∼186 cm^−1^ (CAM-B3LYP/II//GFN2-xTB). The computed values are compared with those from model D6 (also shown in Fig. 6) and experimental values for hydrated HKUST-1.

As discussed above, for HKUST-1 best agreement with experiment has so far been obtained for a model with two copper paddlewheel dimers and a methyl ester substituent (model D6). Given the general improvements seen on moving from models with one dimer to those with two, it may be expected that better agreement will therefore be obtained for HKUST-1 for models that contain three copper paddlewheel dimers (models Ra1 and Ra2, see Fig. 2C and Table 1). The expression used to determine the total shielding has to be further adapted to take into account the increased number of spin configurations (as discussed in detail in Section S5 of the ESI†). For a model loaded with six water molecules (model Ra2) the guests can be oriented to maintain approximate three-fold symmetry, allowing a simplification of the expression use to calculate the total shielding (see eqn S26†). Shifts calculated using this expression are given in Table S27,† and result in a slightly different *s* (of 1.141). For singlet configurations, models with and without hydration (models Ra1 and Ra2) result in very similar shieldings for the three C sites (within ∼2 ppm), while for high-spin configurations much larger differences can occur (up to 530 ppm for C1, as shown in Tables S25 and S26†). The as-calculated Δ*E*_ST_ for the hydrated model is ∼186 cm^−1^, which is ∼33 cm^−1^ smaller than for the dehydrated model (most likely due to an electrostatic interaction with the guest), and is very similar to that seen for models D4–D6, as discussed above. Fig. 8 shows that ^13^C shifts computed for the hydrated tri-dimer model (Ra2) for HKUST-1 (*s* = 1.141) reproduce reasonably well the experimental results (see also Table S27†), with a MAD lower than 5 ppm at each temperature, and shows an improvement over the results for the corresponding di-dimer model (D6) in the range 250–273 K. Note that the experimental results are collected from two series of experiments with slightly different conditions that may lead to small internal inconsistencies (see ref. 30 for details).

#### Ring models

**Fig. 9:**
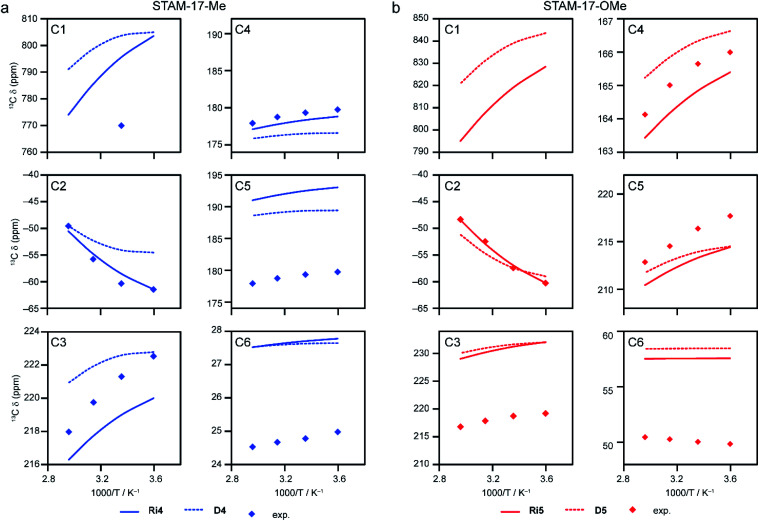
(a) Temperature dependence of calculated ^13^C shifts for model Ri4 (Table S30†), using eqn S26,† with energy gaps scaled by 1.204 on an intradimer coupling of ∼184 cm^−1^ (CAM-B3LYP/II//GFN2-xTB). The results are compared with calculated ^13^C shifts for model D4 (Table S15†) and experimental values for hydrated STAM-17-Me.^16^ Note that, for C4 and C5, only a single resonance is observed experimentally and the blue points are the same in both plots. (b) Temperature dependence of calculated ^13^C shifts for model Ri5 (Table S32†), using eqn S26,† with energy gaps scaled by 1.183 on an intradimer coupling of ∼183 cm^−1^ (CAM-B3LYP/II//GFN2-xTB). The results are compared with calculated ^13^C shifts for model D5 (Table S18†) and experimental values for hydrated STAM-17-OMe.^16^ Note that the signal for C1 was not observed experimentally in ref. 16.

An alternative model for Cu-dimer based MOFs involves three paddlewheel dimers joined by three linkers, forming a closed ring as shown in Fig. 2D (models Ri4–Ri6). See Section S5 of the ESI† for further discussion of the spin configurations and the scaling factors. Fig. 9A compares ^13^C shifts calculated (using eqn S26†) for model Ri4, with six water molecules attached, to the experimental shifts for STAM-17-Me, and shows better agreement than model D4. This can also be seen by comparing the lower MADs in Tables S15 and S30.† Note that values for Δ*E*_ST_ are not affected by the number of dimers in a model. However, these energies change significantly upon the coordination of guests (for water the as-calculated Δ*E*_ST_ changes from 214 cm^−1^ to 184 cm^−1^). Replacing the Me substituent with OMe (model Ri5, with OMe groups oriented such that approximate C_3_ symmetry is preserved) provides the corresponding model for STAM-17-OMe, and leads to only to a very small change (∼1 cm^−1^) in Δ*E*_ST_. Comparison of calculated and experimental shifts leads to a scaling factor of 1.183, close the 1.204 determined for Ri4. Fig. 9B shows that when compared to model D5, the computed shifts show a similar improvement in the agreement with experiment to that seen for D4/Ri4 in Fig. 9A, with lower MADs at each temperature (see Tables S18 and S32†).

The advantage of the tri-dimers over the di-dimer models is that they show better agreement with the experimental shifts observed at low temperatures. Adding the third dimer to the model increases the number of spin configurations and states. As the tri-dimer models have more intermediate and high spin states that can be populated, at lower temperature the tri-dimer models have relatively higher contributions from the paramagnetic shifts of these excited states than those of the di-dimer model, which apparently improves the agreement with experiment. These ring tri-dimer models would, thus, appear to be suitable for computing ^13^C shifts for the STAM-17 series.

Replacement of the Me/OMe groups with CO_2_Me gives a model for STAM-1 (model Ri6 in Fig. 2D and Table 1). As shown in Fig. S19,† the ^13^C shifts (calculated using eqn S26†) are in qualitative agreement with experiment (see Table S33† for calculated shieldings). For C2 there is a noticeable improvement when compared to the corresponding di-dimer model (D6) while, in contrast, for C3 and C4 the simpler model better reproduces the experimental temperature dependence of the shifts (see Fig. S19†), although these changes are smaller (2 to 5 ppm) within the temperature range considered.

In general, the ^13^C shifts of hydrated STAM MOFs are reproduced well using models containing two paddlewheel dimers or the Ri tri-dimer models. The assumption that shifts can be computed by Boltzmann averaging of selected spin states that are constructed from all possible spin configurations can lead to a remarkably good agreement with experiment. Although these tri-dimer models tend to perform better than di-dimer models, the latter can still lead to fairly good agreement with experimental VT NMR results. As these simpler models have far fewer spin configurations, their relatively lower cost makes them potentially useful and attractive models for further study. In contrast, however, a ring tri-dimer model appears to be less successful than the simpler di-dimer model for computing ^13^C shifts in HKUST-1 (see Fig. S18†). Although this framework does contain subunits of rings with three dimers, rings containing four dimers joined by four linkers are also present, and it may be that the ring model is not a sufficiently accurate representation of the complete structure. It could be possible to extend the investigation to consider ring models with four dimers (although at considerable cost given the larger number of spin states). However, reasonably good agreement with experiment is seen for the radial tri-dimer model Ra1, with much improved shifts for C1 (as shown in Fig. S16†). This model may provide a better picture of the local environment experienced by any one linker in HKUST-1, with any improvement in the description of the longer-range structure provided by a Ri tri-dimer model offset by only having two thirds of the required dimers bound to each linker. Thus, building larger molecular models for MOFs would not only require adding more dimers with an increasing number of spin configurations but the degree of success would also depend on the connectivity of the dimers in the material of interest.

#### Scaling of the intradimer coupling energy

**Fig. 10:**
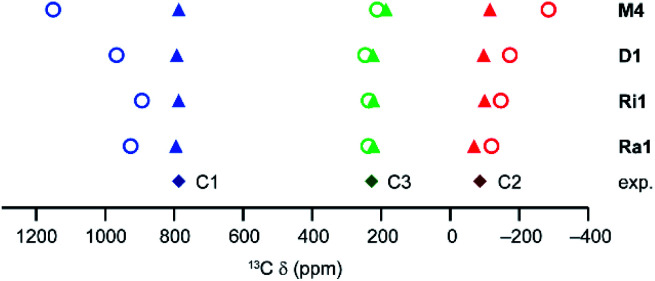
As-calculated (open circles) and scaled (filled triangles) computed ^13^C shifts for different molecular models of activated HKUST-1 at 298.2 K (CAM-B3LYP/II//GFN2-xTB). The energy scaling factors are 1.73, 1.30, 1.20 and 1.23 for the M4, D1, Ri1 and Ra1 models, respectively, determined as described above. Experimental points for activated HKUST-1 (298 K) are shown as filled diamonds.

In the discussion above, an empirical scaling factor of the relative energies of the spin states has been used to increase the energy gap between states, which provides better agreement between the computational and experimental ^13^C shifts (see *e.g.*, Fig. S7 and S14†). Such an approach was not needed for the molecular copper benzoates studied in previous work,^20^ where the spin centres are chemically isolated. The effect of this scaling is to vary the population of the higher spin states and thereby tune the contribution of these to the observed shifts. A molecular model built from a finite number of dimers has only a limited number of spin states, compared to the countless number for the target extended MOFs. A proper description of such spin states in infinite periodic systems and the effects on macroscopic observables such as pNMR shifts is still an area of debate and current development.^24,51^ As shown in Fig. 10 for activated HKUST-1, as the number of dimers in a model increases the as-calculated ^13^C shifts become more similar to those seen in experiment (see Table S34†), and the scaling factor required to improve agreement between calculation and experiment tends towards unity. However, Δ*E*_ST_ is almost unchanged at ∼200 cm^−1^. This would suggest that better agreement between calculated and observed shifts would require a better representation of the equilibrium spin states in the MOF model used.

## Conclusions

In this work, an efficient protocol for the calculation of pNMR shifts in materials containing multiple Cu(ii) paddlewheel dimers is proposed. This neglects any effect of ZFS (which is shown to be very small), uses a cheap but reliable method for geometry optimisation (GFN2-xTB for the high-spin state), a simplified structural model and a promising DFT functional (CAM-B3LYP) for computing the NMR and EPR parameters.

The use of molecular models containing two or three dimers has been validated by comparing calculated and experimental ^13^C pNMR shifts for hydrated and activated HKUST-1 and hydrated STAM MOFs. The success of this approach requires a consideration of the relevant spin configurations in a broken symmetry (BS) approach. High-spin configurations where all centres have the same spin can be well described using a single determinant; however there remains ambiguity over which BS determinants would contribute to the multi-determinantal wavefunctions of the low- or intermediate-spin states. The number of possible configurations grows rapidly with the number of dimer units in a model. In the absence of sophisticated configuration interaction calculations (which would provide information on the contribution of individual configurations to specific states, but at prohibitive computational cost), this work has explored a number of possible approaches by assuming plausible linear combinations of configurations for which magnetic shieldings can then be averaged. The total shielding is then evaluated from a Boltzmann distribution between the energy levels of the chosen configurations. The choice of the configurations that are combined affects the weightings in the expression for the final shielding and, thus, the overall calculated pNMR shift. Owing to thermal averaging the computed pNMR shifts are very sensitive to temperature and, therefore, to the relative energies of the BS spin states. However, in order to reproduce the temperature dependence of the pNMR shifts seen in experiment, some scaling of the as-calculated energy gaps is required. A single scaling factor was applied to all levels in any one system, by fitting to experimental results at several temperatures simultaneously. Even with such an approach, the predicted temperature dependence of the shifts do still depend to some extent on the choices of spin configurations that are combined, with best agreement found when only pairs of configurations are used. This leads to the use of eqn S8 and S19† to calculate ^13^C isotropic shifts for models with two and three dimers, respectively. Where possible, further simplifications can be introduced by exploiting the symmetry (exact or approximate) of a model to reduce the number of spin configurations considered (*e.g.*, *C*_s_ and *C*_3_ symmetry for models with two and three dimers). This leads to the simplified expressions in eqn S15 and S26,† which provide results that are very similar to the more general expressions in eqn S8 and S19,† respectively.

This methodology provides good agreement between experimental shifts for hydrated HKUST-1 and MOFs in the STAM series, and for activated HKUST-1, with those predicted using models containing two Cu(ii) dimers (note the phase change seen for crumple zone MOFs upon activation prevents any simple comparison for these materials). Although more computationally expensive, radial models with three dimer units on the same linker perform well for HKUST-1. For STAM MOFs, a ring model, with three Cu(ii) dimers joined by three linkers (a structural motif found in the MOFs), showed improved agreement with experiment relative to a model with two dimer units (although at a higher computational cost). In contrast, however, this model performed less well for HKUST-1 (where the radial model was much better). Although this motif is present in HKUST-1, all linkers in the MOF have three dimer substituents, and these results suggest this contribution to the shifts is greater than that of a more accurate longer-range structure. It should be noted that the scaling factor required to improve agreement between calculation and experiment decreases with an increasing number of dimer units in the model (*e.g.*, from ∼1.7 for mono-dimer models to 1.2 for tri-dimer models). The approach of this scaling factor towards unity indicates that models with three dimers are approaching a size where they can be considered as reasonable models for infinite MOFs, at least as far as the ^13^C shifts are concerned. The methodology we have introduced for accurate prediction of NMR parameters in systems with weakly coupled pairs of interacting spins has great potential for the study of more complex and challenging chemistry, including host–guest interactions, hydration, activation and phase transitions in MOFs. The insight we gain from the approaches introduced and validated here should also inform the future method development required for materials with more complex spin systems.

## Data availability

The research data supporting this publication can be accessed at https://doi.org/10.17630/233aeedf-1dc4-49e4-b5b8-2c136b2e2365 [Ref. 57].

## Author contributions

S. E. A. and D. M. D. designed and carried out the experimental work, Z. K. and M. B. designed and carried out the computational work. All authors made significant contributions to the writing process.

## Conflicts of interest

There are no conflicts to declare.

## Supplementary Material

SC-013-D1SC07138F-s001
