# Supplementary material for: Origin of the temperature dependence of 13C pNMR shifts for copper paddlewheel MOFs
Source: Chem Sci. 2022 Feb 3;13(9):2674–85. doi: 10.1039/d1sc07138f (PMC8890090; doi:10.1039/d1sc07138f)
Supplement: SC-013-D1SC07138F-s001 [file SC-013-D1SC07138F-s001.pdf]

Electronic Supplementary Material (ESI) for Chemical Science.
This journal is © The Royal Society of Chemistry 2022

Origin of the Temperature Dependence of ^{13}C pNMR Shifts for Copper Paddlewheel MOFs

Zhipeng Ke,[1] Daniel M. Dawson,[1] Sharon E. Ashbrook,[1*] and Michael Bühl[1*]

[1]*School of Chemistry, EaStCHEM and Centre of Magnetic Resonance, University of St Andrews,*
St Andrews KY16 9ST, United Kingdom

Supporting Information

S1. Complete reference 31

S2. Models with a single copper paddlewheel dimer

S3. Models with two copper paddlewheel dimers

S4. Effects of hydration on aromatic resonances

S5. Models with three copper paddlewheel dimers

S6. References

S1. Complete Reference 31

M. J. Frisch, G. W. Trucks, H. B. Schlegel, G. E. Scuseria, M. A. Robb, J. R. Cheeseman, G. Scalmani, V. Barone, B. Mennucci, G. A. Petersson, H. Nakatsuji, M. Caricato, X. Li, H. P. Hratchian, A. F. Izmaylov, J. Bloino, G. Zheng, J. L. Sonnenberg, M. Hada, M. Ehara, K. Toyota, R. Fukuda, J. Hasegawa, M. Ishida, T. Nakajima, Y. Honda, O. Kitao, H. Nakai, T. Vreven, J. A. Montgomery, J. E. Peralta, F. Ogliaro, M. Bearpark, J. J. Heyd, E. Brothers, K. N. Kudin, V. N. Staroverov, T. Keith, R. Kobayashi, J. Normand, K. Raghavachari, A. Rendell, J. C. Burant, S. S. Iyengar, J. Tomasi, M. Cossi, N. Rega, J. M. Millam, M. Klene, J. E. Knox, J. B. Cross, V. Bakken, C. Adamo, J. Jaramillo, R. Gomperts, R. E. Stratmann, O. Yazyev, A. J. Austin, R. Cammi, C. Pomelli, J. W. Ochterski, R. L. Martin, K. Morokuma, V. G. Zakrzewski, G. A. Voth, P. Salvador, J. J. Dannenberg, S. Dapprich, A. D. Daniels, O. Farkas, J. B. Foresman, J. V. Ortiz, J. Cioslowski, D. J. Fox, *Gaussian Inc.*, 2013.

S2. Models with a single copper paddlewheel dimer

A single paddlewheel dimer is the simplest model for calculating ^{13}C chemical shifts of aromatic carbon sites for HKUST-1, using a factor s to scale the energy separation to the paramagnetic state. In this case, there are two spin states: a singlet and a triplet. The triplet state is well represented by a single configuration, *e.g.*, Triplet(1) in **Fig. S1**. The singlet state is a linear combination of the two configurations Singlet(1) and Singlet(2). The shielding constants of the singlet state can be approximated through those of the BS-KS determinants of the constituent configurations, *i.e.*,

$$\sigma_{singlet} = \tfrac{1}{2}(\sigma_{singlet(1)} + \sigma_{singlet(2)}) \,, \qquad \textbf{Eq. S1}$$

which, because both are devoid of pNMR contributions and contain only a σ_{orb} term, are identical, so that only one needs to be evaluated and $\sigma_{singlet}$ becomes

$$\sigma_{singlet} = \sigma_{singlet(1)} \,. \qquad \textbf{Eq. S2}$$

The ^{13}C shifts are calculated using this model as a combination of the shifts of Boltzmann populated spin configurations (with the energy scaled as in **Eq. 3**).

$$\sigma_{total} = f_{singlet}\,\sigma_{singlet} + f_{triplet}\,\sigma_{triplet} \,, \qquad \textbf{Eq. S3}$$

Table S1. Structural differences in mono-dimer models and CPU time of calculations.[a]

Model	rings	carboxylates	CPU time of geometry optimisation / min	CPU time of magnetic properties calculation / h[b]
**M1**	4	4	3	45.5
**M2**	4	12	17	122.9
**M3**	2	4	2	20.9
**M4**	1	4	2	15.0

[a] On a 12-core Xeon cluster node. [b] Includes orbital shielding calculated with GAUSSIAN09, and EPR tensor and singlet-triplet energy gap calculations with ORCA.

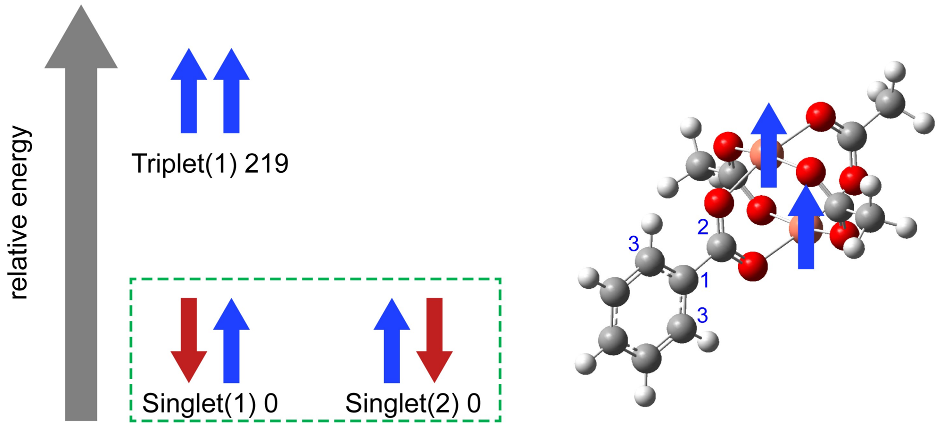

Fig. S1. Spin configurations of model **M4** (see **Fig. 2** and **Table 1**). Blue and red arrows represent the spin states of the unpaired electrons. The dashed box indicates a combination of configurations into a single spin state. The number in parentheses labels the configuration. The following number is the energy (in cm^{-1}) of that configuration relative to the ground state, evaluated at the CAM-B3LYP/II level using the GFN2-xTB optimised geometry. Atoms are coloured brown = Cu, red = O, grey = C and white = H.

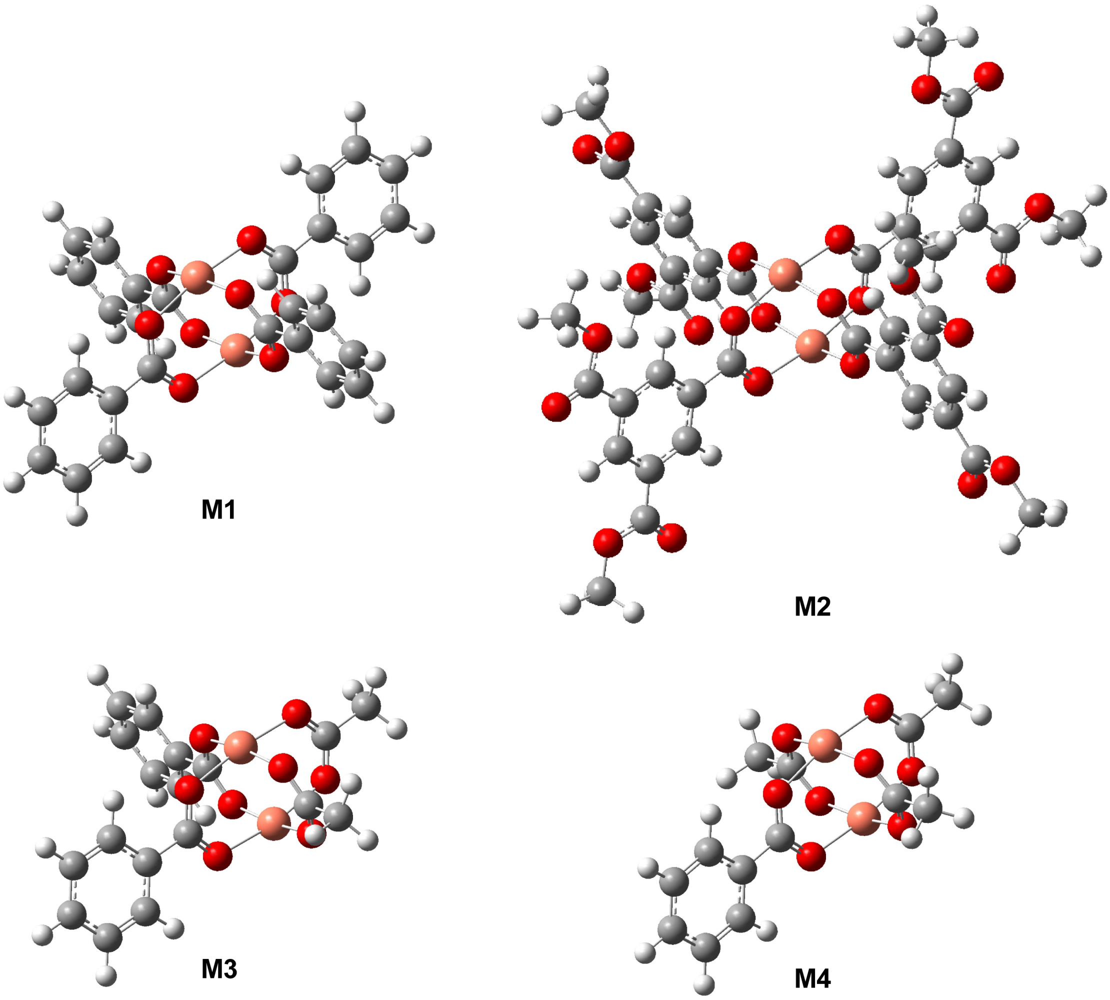

Fig. S2. GFN2-xTB optimised structures of models **M1-M4** (see **Fig. 2** and **Table 1**). Atoms are coloured brown = Cu, red = O, grey = C and white = H.

Table S2. Cu-Cu distance and ΔE_{ST} for the mono-dimer models.

Model	Distance / Å	ΔE_{ST} / cm^{-1}	sΔE_{ST} / cm^{-1} [a]
**M2**	2.611	205	398
**M1**	2.591	208	387
**M3**	2.600	215	383
**M4**	2.605	219	379

[a] Note that the scaled singlet-triplet energy gaps, sΔE_{ST}, are much closer to experimental exchange couplings in MOFs (for example –334 cm^{-1} in STAM-1)[S1] than the unscaled ΔE_{ST}.

Table S3. Fitted computed ^{13}C shifts (in ppm) for model **M4** at 298.2 K, obtained by introducing Weiss constant (Θ) or scaling the as-calculated ΔE_{ST}. The experimental shifts of activated HKUST-1 are also quoted for comparison.

Site	Θ = –170 K	$\Delta E_{ST} = 1.73\ \Delta E_{ST(as\text{-}calc.)}$	Exp.
C1	785.1	786.4	786.4
C2	–114.6	–115.2	–86.2
C3	170.4	170.4	227.5

Table S4. Computed ^{13}C shifts (in ppm) of models **M1** to **M4** at 298.2 K with respective scaling factors, s, determined by minimising the MAD of all resonances for three carbon sites of the model.

	exp.	**M1**	**M2**	**M3**	**M4**
s	/	1.86	1.94	1.78	1.73
C1	786.4	786.4	786.4	786.4	786.4
C2	–86.2	–126.0	–103.2	–120.4	–115.2
C3	227.5	185.5	191.2	185.5	186.1

S3. Models with two copper paddlewheel dimers

Expressions for calculating ^{13}C shifts for di-dimer models

Increasing the number of dimers in a model introduces more spin configurations. The important question is, how many of these configurations combine into how many distinct spin states. For instance, Singlet(1) - Singlet(6) in **Fig. S3** all have the same multiplicity, but it is reasonable to assume that configurations Singlet(5) and Singlet(6) describe a different electronic state than Singlet(1) - Singlet(4) because, in the former the spins within a single dimer are uncoupled, whereas in the latter they are paired. The state with the uncoupled spins, Singlet(6), with the spin distribution [↑↑][↓↓] (where the square brackets group spins from the same dimer), would be expected to constitute an excited singlet state, together with the corresponding configuration where all spins are flipped, Singlet(5). Although the shielding constants of these BS-KS configurations are identical, the spin configurations are two different spin states (see below for analogous Singlet(5) and Singlet(6) of the tri-dimer model in **Fig. S13**).

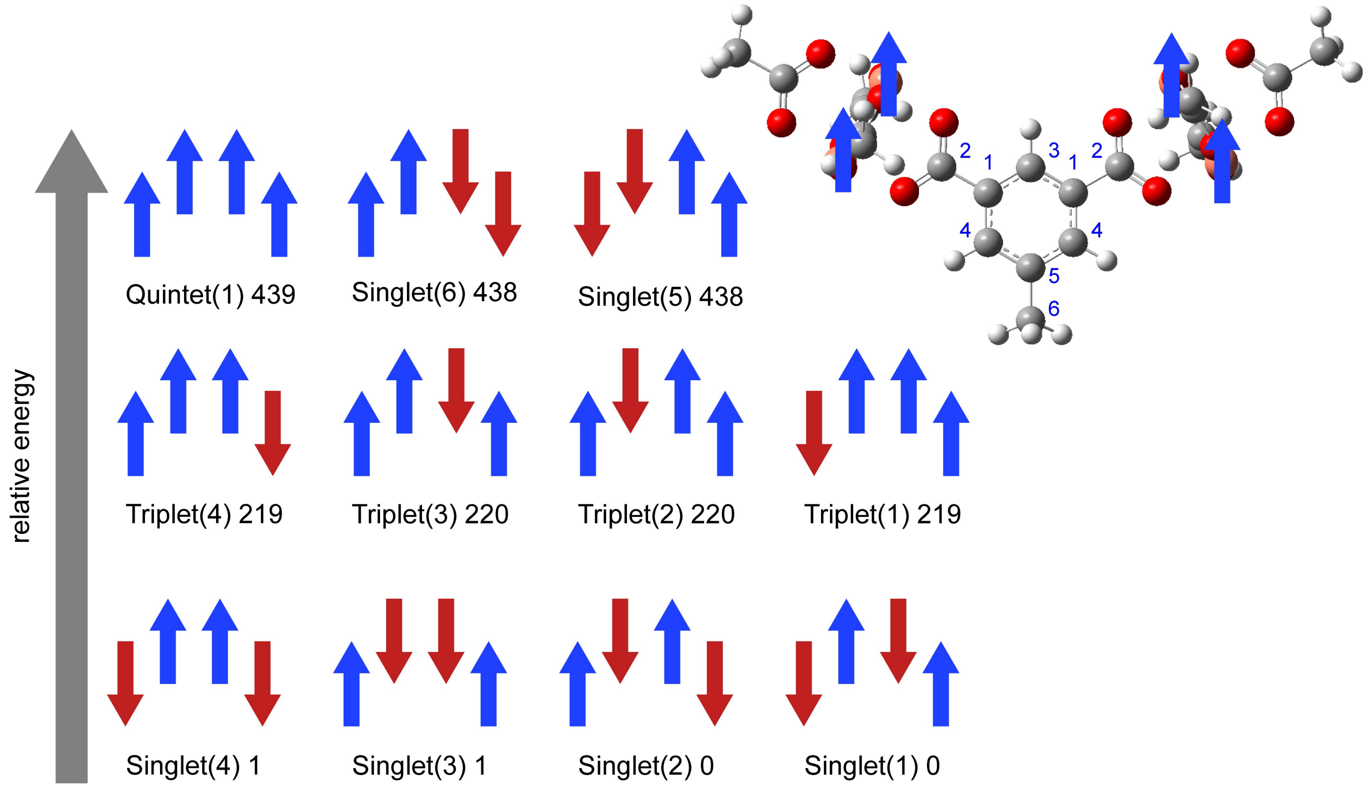

Fig. S3. Spin configurations of model **D1** (see **Fig. 2** and **Table 1**) All nomenclature and colouring schemes are as in **Fig. S1**.

The question now is whether configurations such as, *e.g.*, Singlet(1) and Singlet(2) belong to two distinct states [↓↑][↓↑], [↑↓][↑↓], or, more likely, to a single state (each in combination with the configurations where all spins are flipped, *i.e.*, [↓↑][↓↑]+[↑↓][↑↓] for Singlet(1) and Singlet(2), and [↓↑][↑↓]+[↑↓][↓↑] for Singlet(3) and Singlet(4), as shown schematically in **Fig. S4**), or whether they combine into a more complicated state (*i.e.*, [↓↑][↓↑]+[↑↓][↑↓]+[↓↑][↑↓]+[↑↓][↓↑]). In the first case, the shielding constants of these singlet states would be obtained as

$$\sigma_1 = \tfrac{1}{2}(\sigma_{singlet(1)} + \sigma_{singlet(2)}) \text{ and } \sigma_2 = \tfrac{1}{2}(\sigma_{singlet(3)} + \sigma_{singlet(4)}) \ , \qquad \textbf{Eq. S4}$$

whereas, in the latter case, the shielding constant would be

$$\sigma = \tfrac{1}{4}(\sigma_{singlet(1)} + \sigma_{singlet(2)} + \sigma_{singlet(3)} + \sigma_{singlet(4)}) \ . \qquad \textbf{Eq. S5}$$

In practice, $\sigma_{singlet(1)}$ will be very similar, essentially indistinguishable, from the shielding constants of the singlet configurations Singlet(2)-Singlet(4), so that all conceivable singlet states based on these configurations will have essentially the same σ values. However, their weights in the final pNMR expression will depend on whether they are treated as a single state (the ground state), or as two distinct, near-degenerate states. The same considerations apply to the triplet configurations Triplet(1)-Triplet(4) in **Fig. S5**.

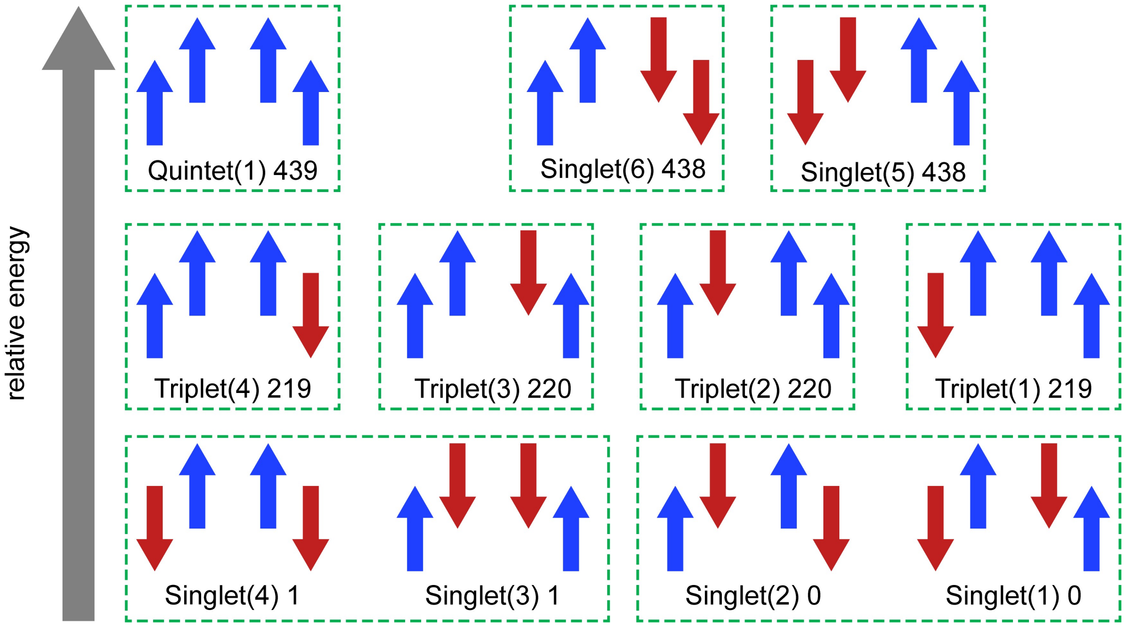

Fig. S4. Spin configurations of model **D1**, as shown in **Fig. S3**. Dashed boxes indicate configurations that are combined into a spin state in **Eq. S7**.

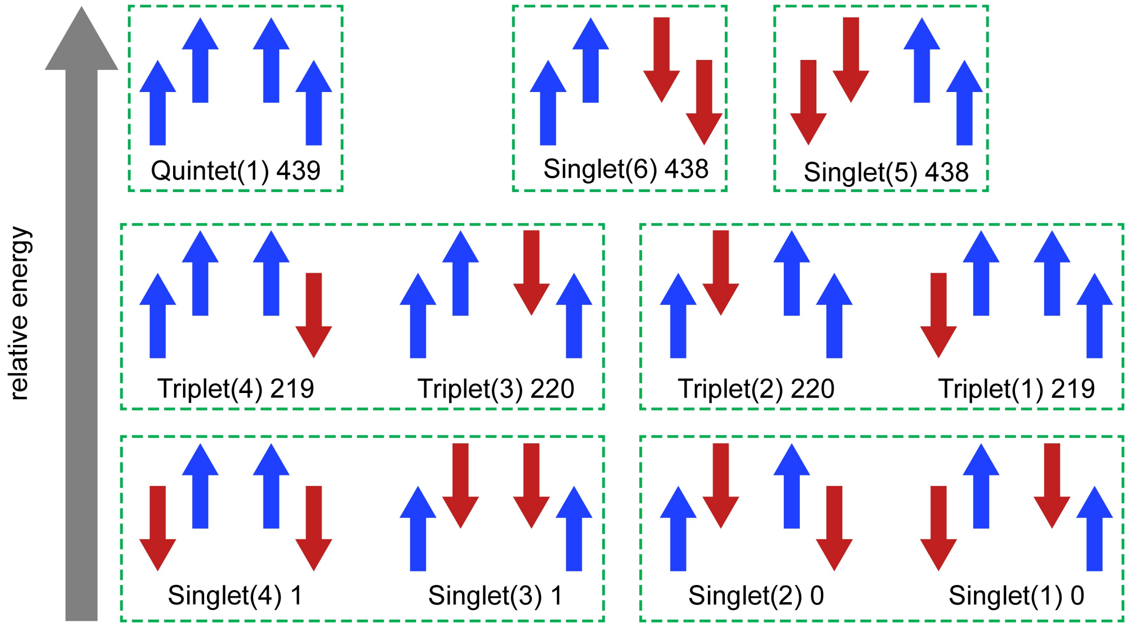

Fig. S5. Spin configurations of model **D1**. Dashed boxes indicate configurations that are combined into a spin state in **Eq. S8**.

In principle, this question could be addressed with suitable multi-reference configuration interaction calculations, but in practice, the systems are much too big for any sensible calculations of this kind. We will therefore explore a variety of scenarios assuming a predefined set of electronic states that are composed of selections from the possible electron configurations. We will try to validate each of these models through comparison of the resulting chemical shifts with experimental data for water-loaded STAM-1, the STAM-17 series and HKUST-1, as well as for dehydrated (activated) HKUST-1.

Di-dimer models **D1**-**D3** (See **Fig. 2** and **Table 1**) were designed for the STAM MOF series and these can also be investigated as models for HKUST-1. The total number of spin configurations is eleven (eight excluding the configurations of the singlets where all spins are flipped) for the di-dimer models and the configurations are shown in **Fig. S3**. First assuming each corresponds to a distinct electronic state (**Fig. S3**), the following expression for σ_{total} is obtained:

$$\sigma_{total} = [f_{S(1)}\,\sigma_{S(1)} + f_{S(2)}\,\sigma_{S(2)} + f_{S(3)}\,\sigma_{S(3)} + f_{S(4)}\,\sigma_{S(4)} + f_{S(5)}\,\sigma_{S(5)} + f_{S(6)}\,\sigma_{S(6)} + f_{T(1)}\,\sigma_{T(1)} + f_{T(2)}\,\sigma_{T(2)} + f_{T(3)}\,\sigma_{T(3)} + f_{T(4)}\,\sigma_{T(4)} + f_{Q(1)}\,\sigma_{Q(1)}] / [f_{S(1)} + f_{S(2)} + f_{S(3)} + f_{S(4)} + f_{S(5)} + f_{S(6)} + f_{T(1)} + f_{T(2)} + f_{T(3)} + f_{T(4)} + f_{Q(1)}] , \qquad \textbf{Eq. S6}$$

where the subscript capital letter denotes the spin state (S for singlet, T for triplet and Q for quintet) and the number in bracket denotes the spin configuration.

This expression assumes the presence of six distinct singlet states and four distinct triplets. The next step is to explore the consequences of combining some of these into fewer electronic states that are made up of more individual configurations. Specifically, as discussed above, in analogy to the open-shell singlet in a single dimer, which is modelled through the combination of ↓↑+↑↓, these singlet configurations can be combined where spins are coupled within a dimer (**Fig. S4**). This leads to the following expression for σ_{total}:

$$\sigma_{total} = [\tfrac{1}{2}(f_{S(1)} + f_{S(2)})\ \tfrac{1}{2}(\sigma_{S(1)} + \sigma_{S(2)}) + \tfrac{1}{2}(f_{S(3)} + f_{S(4)})\ \tfrac{1}{2}(\sigma_{S(3)} + \sigma_{S(4)}) + f_{S(5)}\ \sigma_{S(5)} + f_{S(6)}\ \sigma_{S(6)} + f_{T(1)}\ \sigma_{T(1)} + f_{T(2)}\ \sigma_{T(2)} + f_{T(3)}\ \sigma_{T(3)} + f_{T(4)}\ \sigma_{T(4)} + f_{Q(1)}\ \sigma_{Q(1)}] / [\tfrac{1}{2}(f_{S(1)} + f_{S(2)}) + \tfrac{1}{2}(f_{S(3)} + f_{S(4)}) + f_{S(5)} + f_{S(6)} + f_{T(1)} + f_{T(2)} + f_{T(3)} + f_{T(4)} + f_{Q(1)}]\ . \quad \textbf{Eq. S7}$$

With the same reasoning, the four triplet configurations can be combined into two triplet states consisting of two configurations each (see **Fig. S5**), namely the linear combination of Triplet(3)+Triplet(4) and that of Triplet(1)+Triplet(2), which would give the expression:

$$\sigma_{total} = [\tfrac{1}{2}(f_{S(1)} + f_{S(2)})\ \tfrac{1}{2}(\sigma_{S(1)} + \sigma_{S(2)}) + \tfrac{1}{2}(f_{S(3)} + f_{S(4)})\ \tfrac{1}{2}(\sigma_{S(3)} + \sigma_{S(4)}) + f_{S(5)}\ \sigma_{S(5)} + f_{S(6)}\ \sigma_{S(6)} + \tfrac{1}{2}(f_{T(1)} + f_{T(2)})\ \tfrac{1}{2}(\sigma_{T(1)} + \sigma_{T(2)}) + \tfrac{1}{2}(f_{T(3)} + f_{T(4)})\ \tfrac{1}{2}(\sigma_{T(3)} + \sigma_{T(4)}) + f_{Q(1)}\ \sigma_{Q(1)}] / [\tfrac{1}{2}(f_{S(1)} + f_{S(2)}) + \tfrac{1}{2}(f_{S(3)} + f_{S(4)}) + f_{S(5)} + f_{S(6)} + \tfrac{1}{2}(f_{T(1)} + f_{T(2)}) + \tfrac{1}{2}(f_{T(3)} + f_{T(4)}) + f_{Q(1)}]\ . \quad \textbf{Eq. S8}$$

Assuming Singlet(1) - Singlet(4) to be part of the same state, labelled Singlet(a), Singlet(5) - Singlet(6) to be part of another singlet state, labelled Singlet(b), Triplet(1) - Triplet(4) to be part of a single triplet state, labelled Triplet, and Quintet(1) being the same as before (**Fig. S6**), the corresponding shielding constants evaluated as follows,

$$\sigma_{S(a)} = \tfrac{1}{4}\ [\sigma_{S(1)} + \sigma_{S(2)} + \sigma_{S(3)} + \sigma_{S(4)}]\ , \quad \textbf{Eq. S9}$$

$$\sigma_{S(b)} = \tfrac{1}{2}\ [\sigma_{S(5)} + \sigma_{S(6)}]\ , \quad \textbf{Eq. S10}$$

$$\sigma_{T} = \tfrac{1}{4}\ [\sigma_{T(1)} + \sigma_{T(2)} + \sigma_{T(3)} + \sigma_{T(4)}]\ , \quad \textbf{Eq. S11}$$

the following expression for the total shielding is obtained:

$$\sigma_{total} = [\tfrac{1}{4}(f_{S(1)} + f_{S(2)} + f_{S(3)} + f_{S(4)})\ \sigma_{S(a)} + \tfrac{1}{2}(f_{S(5)} + f_{S(6)})\ \sigma_{S(b)} + \tfrac{1}{4}(f_{T(1)} + f_{T(2)} + f_{T(3)} + f_{T(4)})\ \sigma_{T} + f_{Q(1)}\ \sigma_{Q(1)}] / [\tfrac{1}{4}(f_{S(1)} + f_{S(2)} + f_{S(3)} + f_{S(4)}) + \tfrac{1}{2}(f_{S(5)} + f_{S(6)}) + \tfrac{1}{4}(f_{T(1)} + f_{T(2)} + f_{T(3)} + f_{T(4)}) + f_{Q(1)}]\ . \quad \textbf{Eq. S12}$$

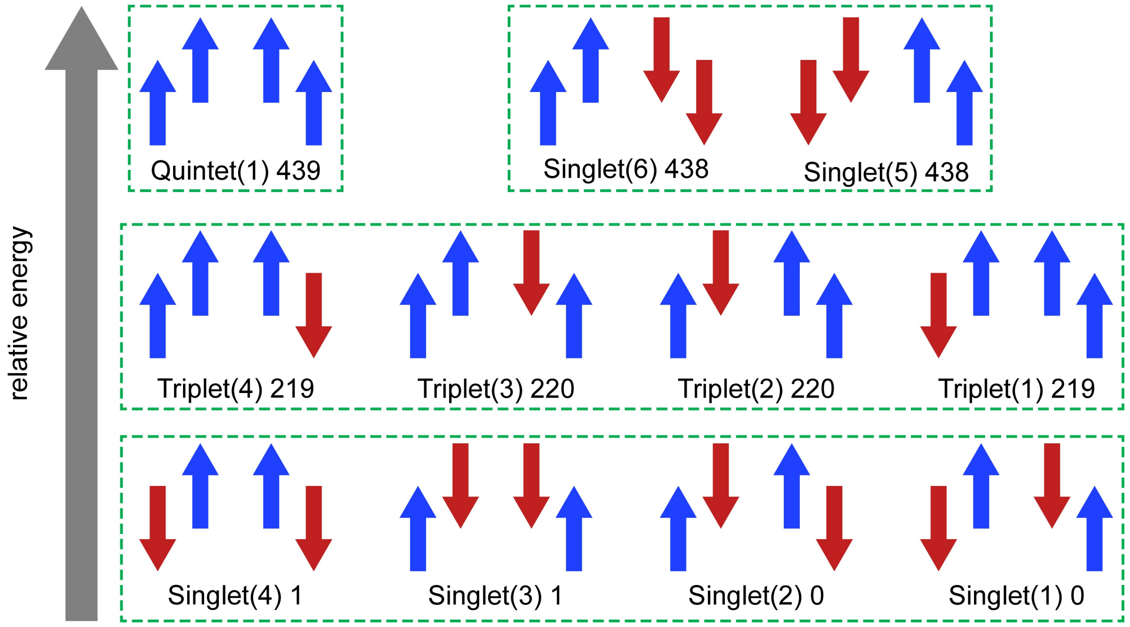

Fig. S6. Spin configurations of model **D1**. Dashed boxes indicate configurations that are combined into a spin state in **Eq. S12**.

Comparing model D1 to activated HKUST-1

These scenarios are now applied to selected models, first for "activated" (*i.e.*, guest-free) MOFs. **Fig. S7** shows computed ^{13}C shifts for model **D1** against 1000/T (in the range 250 K ≤ T ≤ 348 K), without and with scaling, together with the experimental shifts for activated HKUST-1. The unscaled shifts are evaluated according to **Eqs. S6, S7, S8** or **S12** with s = 1. **Table S5** shows the computed ^{13}C shieldings for model **D1** in each spin configuration at 298.2 K, along with the energy of that configuration relative to Singlet(1). These shieldings are used to calculate the shifts (**Table S6**) plotted in **Fig. S7** and following figures of this kind. The scaling is introduced by determining the value of s in **Eq. 3** that leads to the minimum MAD at all temperatures. The values of s obtained are 1.109, 1.789, 1.305 and 1.570 for **Eqs. S6, S7, S8** and **S12**, respectively (see **Table S7** for an example of the scaled energies with s = 1.305). Although the absolute values of the fitted shifts can deviate markedly from the experimental data (particularly for C2, **Fig. S7E**), the experimental trends of the temperature dependence are well reproduced by **Eqs. S7** and **S8**. The calculated shifts from **Eqs. S7** and **S8** are almost equal at low temperatures.

In **Fig. S7D-F**, **Eqs. S7** and **S8** afford similar agreement between computed and experimental shifts, making it hard to decide which expression should be preferred. The MADs for the calculated shifts at each temperature and each carbon site are shown in **Fig. S8**. Treating each spin configuration for the di-dimer model as a distinct electronic

state (**Eq. S6**) does not reproduce the experimental shifts for activated HKUST-1 satisfactorily, and leads to high MADs at lower and higher temperatures. Combining spin configurations into a minimum number of electronic states (**Eq. S12**) worsens the agreement between calculated and experimental ^{13}C shifts and greatly exaggerates their temperature dependencies, leading to an even higher average MAD over the temperature range considered here. The assumption of fewer spin states is, therefore, not supported by these results. The MADs resulting from the use of **Eqs. S7** and **S8** are fairly consistent (at *ca.* 5 ppm) across the whole temperature range. The averaged MAD from **Eq. S8** is slightly lower than that from **Eq. S7**. Moreover, based on the physical meaning of the electronic spin states *i.e.*, considering that singlet spin states should consist of multiple configurations, **Eq. S8**, should perhaps be favoured.

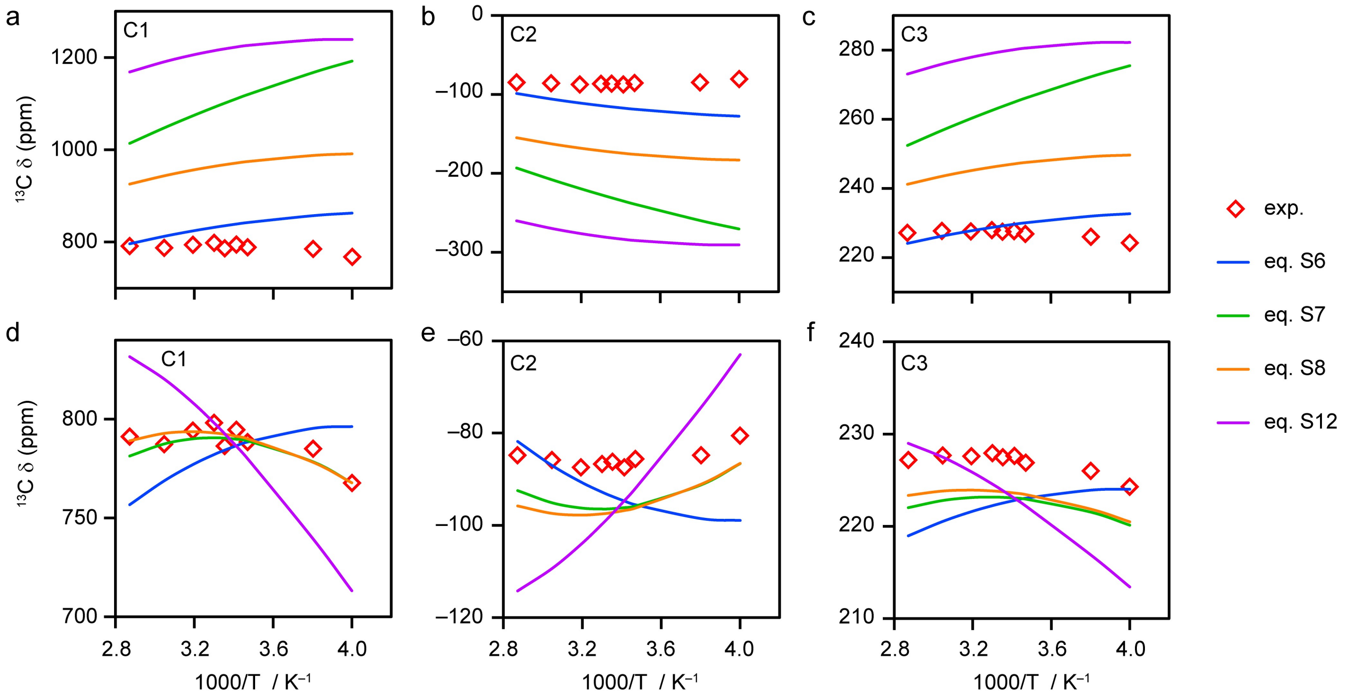

Fig. S7. Plots of temperature variation of the ^{13}C shifts for model **D1**, using **Eqs. S6, S7, S8** or **S12** at the CAM-B3LYP/II//GFN2-xTB level. Shifts were calculated using (a-c) as-calculated ΔE_{ST} (~219 cm^{-1}) and (d-f) scaled (using **Eq. 3**) ΔE_{ST} with s = 1.109, 1.789, 1.305 and 1.570 for **Eqs. S6, S7, S8** and **S12**, respectively. See **Fig. 1D** for the site numbering scheme. Experimental points for activated HKUST-1 are also shown.

Table S5. Shieldings (in ppm) of carbon sites in each spin configuration at 298.2 K and the scaled ΔE_{ST} with respect to the lowest configuration, Singlet(1) (s = 1.305) for model **D1** (CAM-B3LYP/II//GFN2-xTB).

Site	$\sigma_{Q(1)}$	$\sigma_{S(1)\text{-}S(6)}$	$\sigma_{T(1)}$	$\sigma_{T(2)}$	$\sigma_{T(3)}$	$\sigma_{T(4)}$
C1[a]	–3086.2	44.3	–969.4	–1030.3	–1028.4	–968.5
C3	–361.8	48.3	64.8	–240.2	–238.0	64.9
C5	–188.1	41.1	–108.4	34.7	36.9	–105.7
C4[a]	–148.3	43.4	–87.6	48.8	48.9	–86.8
C6	141.2	164.9	148.4	166.7	166.6	147.7
C2[a]	1358.7	3.8	463.8	445.4	449.3	462.9
ΔE / cm^{-1}	572.34	[b]	286.00	286.65	286.61	285.98

[a] Average of two sites in the model. [b] Singlet(1) - Singlet(6): 0.00, 0.00, 0.72, 0.58, 571.90, 571.88.

Table S6. Calculated ^{13}C shifts (in ppm) at different temperatures using model **D1** with s = 1.305 for the as-calculated intradimer coupling of ~219 cm^{-1} (CAM-B3LYP/II//GFN2-xTB), using **Eq. S8**.

T / K	250.0	263.0	288.2	293.0	298.2	303.0	313.2	328.2	348.2
1000/T / K^{-1}	4.0	3.8	3.5	3.4	3.4	3.3	3.2	3.0	2.9
Calc.									
C1	767.7	778.1	790.1	791.3	792.3	793.0	793.6	792.8	789.0
C3	220.5	221.9	223.4	223.6	223.7	223.8	223.9	223.8	223.3
C2	–86.6	–91.1	–96.3	–96.8	–97.2	–97.5	–97.8	–97.4	–95.8
C4[a]	181.8	182.5	183.2	183.3	183.3	183.4	183.4	183.4	183.2
C5[a]	192.3	193.0	193.9	194.0	194.1	194.1	194.2	194.1	193.8
C6[a]	27.2	27.3	27.4	27.4	27.4	27.4	27.4	27.4	27.4
Exp. activated HKUST-1									
(C1)	767.7	785.0	788.4	794.7	786.4	798.1	794.2	787.4	791.2
(C3)	224.3	226.0	226.9	227.6	227.5	227.9	227.6	227.7	227.2
(C2)	–80.5	–84.8	–85.6	–87.4	–86.2	–86.7	–87.4	–85.8	–84.8
MAD[b]	3.3	5.8	5.3	5.6	6.9	6.7	4.9	7.0	5.7

[a] Not present in HKUST-1. [b] For C1, C2 and C3 carbon sites of HKUST-1.

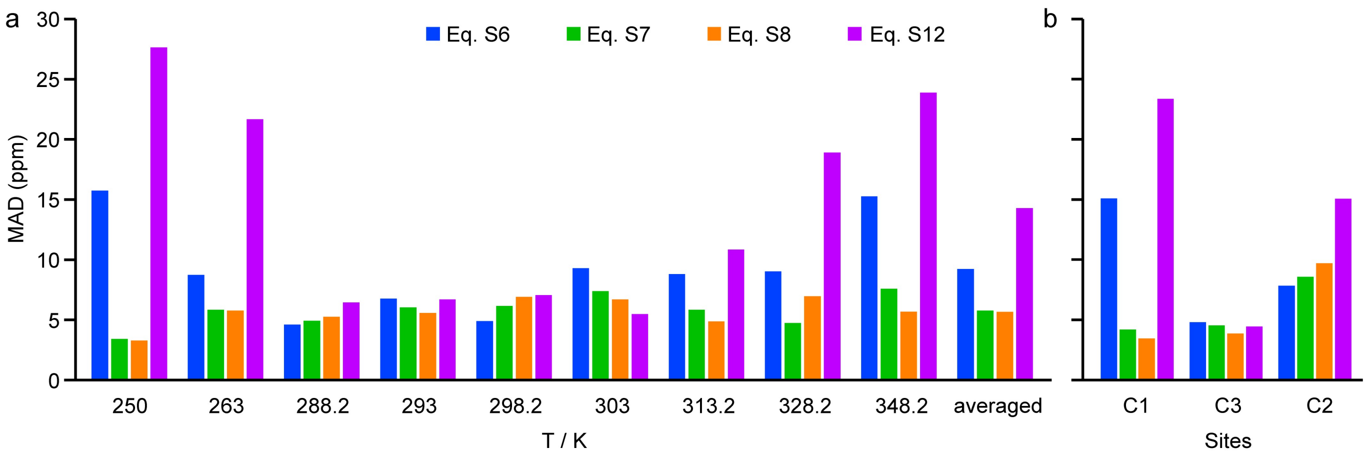

Fig. S8. MADs (a) at each temperature and (b) for sites 1, 2 and 3 in activated HKUST-1, employing **Eqs. S6, S7, S8** or **S12** to calculate ^{13}C shifts for model **D1** with s = 1.109, 1.789, 1.305 and 1.570, respectively (CAM-B3LYP/II//GFN2-xTB).

As model **D1** has approximate C_s symmetry, the two triplet spin states (Triplet(1)+Triplet(2) and Triplet(3) +Triplet(4)) should be equivalent. This is similar for the equivalency of Singlet(5) and Singlet(6). The grouping of Singlet(1)+Singlet(2) and Singlet(3)+Singlet(4) is maintained. Note that the shifts for equivalent nuclei are calculated as average values for the sites in question (see labelling in **Fig. S3**). **Eq. S8** can be simplified to

$$\sigma_{total} = [\tfrac{1}{2}(f_{S(1)} + f_{S(2)})\ \tfrac{1}{2}(\sigma_{S(1)} + \sigma_{S(2)}) + \tfrac{1}{2}(f_{S(3)} + f_{S(4)})\ \tfrac{1}{2}(\sigma_{S(3)} + \sigma_{S(4)}) + 2 \times f_{S(5)}\ \sigma_{S(5)} + 2 \times \tfrac{1}{2}(f_{T(1)} + f_{T(2)})\ \tfrac{1}{2}(\sigma_{T(1)} + \sigma_{T(2)}) + f_{Q(1)}\ \sigma_{Q(1)}] \ / \ [\tfrac{1}{2}(f_{S(1)} + f_{S(2)}) + \tfrac{1}{2}(f_{S(3)} + f_{S(4)}) + 2 \times f_{S(5)} + 2 \times \tfrac{1}{2}(f_{T(1)} + f_{T(2)}) + f_{Q(1)}] \ , \qquad \textbf{Eq. S13}$$

or

$$\sigma_{total} = [\tfrac{1}{2}(f_{S(1)} + f_{S(2)})\ \tfrac{1}{2}(\sigma_{S(1)} + \sigma_{S(2)}) + \tfrac{1}{2}(f_{S(3)} + f_{S(4)})\ \tfrac{1}{2}(\sigma_{S(3)} + \sigma_{S(4)}) + 2 \times f_{S(6)}\ \sigma_{S(6)} + 2 \times \tfrac{1}{2}(f_{T(3)} + f_{T(4)})\ \tfrac{1}{2}(\sigma_{T(3)} + \sigma_{T(4)}) + f_{Q(1)}\ \sigma_{Q(1)}] \ / \ [\tfrac{1}{2}(f_{S(1)} + f_{S(2)}) + \tfrac{1}{2}(f_{S(3)} + f_{S(4)}) + 2 \times f_{S(6)} + 2 \times \tfrac{1}{2}(f_{T(3)} + f_{T(4)}) + f_{Q(1)}] \ . \qquad \textbf{Eq. S14}$$

Both **Eqs. S13** and **S14** have terms for only one pair of triplet configurations, three singlets and one quintet state. The expressions differ in the choice of states involving ferromagnetic coupling on a dimer that are used to model the formally symmetry-equivalent states (*e.g.*, Triplet(1) + Triplet(2) vs. Triplet(3) + Triplet(4)). The factor of 2 in front of terms ($f_{T(i)}\ \sigma_{T(i)}$, i = 1 - 4 and $f_{S(j)}\ \sigma_{S(j)}$, j = 5 - 6) accounts for the other, symmetry-equivalent state. As mentioned above, if the symmetry were exactly C_s, then $\sigma_{S(i)}$ (i = 1 -

6) would be equal, as would $\sigma_{T(1)}$ and $\sigma_{T(4)}$, as well as $\sigma_{T(2)}$ and $\sigma_{T(3)}$ (and **Eqs. S13** and **S14** would be identical). In practice, small deviations from this symmetry lift these degeneracies. For instance, $\sigma_{T(1)}$ and $\sigma_{T(4)}$ are similar but not identical (*e.g.*, see **Table S5** for model **D1**). As shown in **Table S7**, the use of either set of spin states gives a similar quality of results. This simplification reduces the number of triplet configurations that must be calculated, which will be of benefit for the larger tri-dimer models below.

Exploiting the approximate C_s symmetry of model **D4**, **Table S8** shows similar results for this hydrated model when employing the simplified expressions *i.e.*, **Eqs. S13** and **S14**. The shifts of C1/5 and C6 show a noticeable difference of ~10 ppm, depending on which of the triplet pair is included. This is because, the guest water molecules apparently introduce a larger deviation from C_s symmetry.

Table S7. Calculated ^{13}C shifts (in ppm) at different temperatures with fewer spin configurations for model **D1** (CAM-B3LYP/II//GFN2-xTB).

T / K	250.0	263.0	288.2	293.0	298.2	303.0	313.2	328.2	348.2
1000/T / K^{-1}	4.0	3.8	3.5	3.4	3.4	3.3	3.2	3.0	2.9
	T3 and T4 removed[a]								
C1[b]	767.7	778.2	790.1	791.4	792.4	793.1	793.7	792.8	789.1
C3	220.7	222.1	223.6	223.8	223.9	224.0	224.1	224.0	223.5
C2[b]	–86.2	–90.7	–95.9	–96.4	–96.9	–97.1	–97.4	–97.1	–95.5
C4	181.9	182.5	183.3	183.4	183.4	183.5	183.5	183.5	183.3
C5	192.7	193.5	194.4	194.4	194.5	194.6	194.6	194.5	194.2
C6	27.1	27.2	27.3	27.3	27.3	27.3	27.3	27.3	27.3
MAD[c]	3.1	5.6	5.1	5.4	6.7	6.5	4.7	6.8	5.5
	T1 and T2 removed[d]								
C1[b]	767.7	778.1	790.0	791.3	792.3	793.0	793.6	792.7	789.0
C3	220.3	221.7	223.2	223.4	223.6	223.6	223.7	223.6	223.2
C2[b]	–87.0	–91.5	–96.7	–97.2	–97.6	–97.9	–98.2	–97.8	–96.1
C4	181.7	182.4	183.1	183.2	183.3	183.3	183.4	183.3	183.1
C5	191.8	192.6	193.5	193.5	193.6	193.7	193.7	193.7	193.4
C6	27.3	27.4	27.4	27.5	27.5	27.5	27.5	27.5	27.4
MAD[c]	3.5	6.0	5.4	5.8	7.1	6.9	5.1	7.1	5.9

[a] Employing **Eq. S13** with s = 1.305 to minimise MADs. [b] Average of two sites in the model. [c] For C1, C2 and C3 of HKUST-1. [d] Employing **Eq. S14** with s = 1.304 to minimise MADs.

The simplifications from **Eqs. S13** and **S14** cannot be applied for model **D3**, since the CO_2Me substituent breaks the symmetry more than the methyl group. However, a new approximation using an energy difference for a pair of singlet spin configurations, *e.g.*, in model **D3**, using 0.00 cm^{-1} for both Singlet(1) and Singlet(2) instead of using an averaged value of 0.07 and 0.00 cm^{-1}, can be made because the difference of computed results of ^{13}C shifts between these values (within 0.1 cm^{-1}) is symmetry independent and negligible (*cf.* **Tables S9** and **S10**). Note, all possible singlet states will have essentially the same σ values *i.e.*, $\sigma_{S(1)\text{-}S(6)} = \sigma_{S(1)}$. **Eq. S8** can then be simplified to

$$\sigma_{total} = [f_{S(1)}\ \tfrac{1}{2}(\sigma_{S(1)} + \sigma_{S(1)}) + f_{S(3)}\ \tfrac{1}{2}(\sigma_{S(1)} + \sigma_{S(1)}) + f_{S(5)}\ \sigma_{S(1)} + f_{S(5)}\ \sigma_{S(1)} + \tfrac{1}{2}(f_{T(1)} + f_{T(2)})\ \tfrac{1}{2}(\sigma_{T(1)} + \sigma_{T(2)}) + \tfrac{1}{2}(f_{T(3)} + f_{T(4)})\ \tfrac{1}{2}(\sigma_{T(3)} + \sigma_{T(4)}) + f_{Q(1)}\ \sigma_{Q(1)}] / [f_{S(1)} + f_{S(3)} + f_{S(5)} + f_{S(5)} + \tfrac{1}{2}(f_{T(1)} + f_{T(2)}) + \tfrac{1}{2}(f_{T(3)} + f_{T(4)}) + f_{Q(1)}]\ . \qquad \textbf{Eq. S15}$$

Table S8. Calculated ^{13}C shifts (in ppm) at different temperatures with fewer spin configurations of model **D4** for hydrated STAM-17-Me (CAM-B3LYP/II//GFN2-xTB).

T / K	278	298	318	338
T3 and T4 removed[a]				
C1[b]	816.8	814.2	808.2	799.6
C3	229.2	228.7	227.7	226.4
C5	190.0	189.8	189.5	189.0
C4[b]	175.9	175.8	175.5	175.1
C6	27.3	27.3	27.2	27.2
C2[b]	–55.3	–54.5	–52.5	–49.5
MAD	7.8	7.8 (6.5)[c]	7.2	6.5
T1 and T2 removed[d]				
C1[b]	805.9	804.6	799.7	792.1
C3	218.0	218.0	217.5	216.7
C5	189.7	189.7	189.3	188.9
C4[b]	178.0	177.8	177.5	177.1
C6	28.1	28.1	28.0	27.9
C2[b]	–58.3	–57.7	–55.8	–52.9
MAD	4.5	4.2 (9.3)[c]	3.5	4.0

[a] Employing **Eq. S13** with s = 1.445 to minimise the MADs. [b] Average for two sites in the model. [c] The value in brackets includes C1 at 298 K. [d] Employing **Eq. S14** with s = 1.370 to minimise the MADs.

Table S9. Calculated ^{13}C shifts (in ppm) at different temperatures using model **D3** with s = 1.330 for ΔE_{ST} of ~219 cm^{-1} (CAM-B3LYP/II//GFN2-xTB), using **Eq. S8**. Experimental shifts of activated HKUST-1 are also given.

T / K	250.0	263.0	288.2	293.0	298.2	303.0	313.2	328.2	348.2
1000/T / K^{-1}	4.0	3.8	3.5	3.4	3.4	3.3	3.2	3.0	2.9
Calc.									
C1[a]	767.7	779.2	792.9	794.4	795.8	796.7	797.9	797.8	794.9
C3	224.9	226.4	228.1	228.3	228.5	228.6	228.8	228.8	228.4
C2[a]	–68.4	–73.0	–78.5	–79.2	–79.7	–80.1	–80.6	–80.5	–79.4
C4[a]	182.6	183.3	184.1	184.2	184.3	184.4	184.5	184.5	184.3
C5	184.8	185.6	186.6	186.7	186.8	186.9	187.0	187.0	186.8
C6	182.2	182.3	182.5	182.6	182.6	182.6	182.6	182.6	182.6
C7	54.8	54.8	54.9	54.9	54.9	54.9	54.9	54.9	54.9
Exp. activated HKUST-1									
C1	767.7	785.0	788.4	794.7	786.4	798.1	794.2	787.4	791.2
C3	224.3	226.0	226.9	227.6	227.5	227.9	227.6	227.7	227.2
C2	–80.5	–84.8	–85.6	–87.4	–86.2	–86.7	–87.4	–85.8	–84.8
MAD[b]	4.2	6.0	4.3	3.0	5.6	2.9	3.9	5.6	3.4

[a] Average of two sites in the model. [b] Calculated for C1, C2 and C3 of activated HKUST-1.

Table S10. Calculated ^{13}C shifts (in ppm) at different temperatures using model **D3** with s = 1.330 for ΔE_{ST} of ~219 cm^{-1} (CAM-B3LYP/II//GFN2-xTB), using **Eq. S15**.

T / K	250.0	263.0	288.2	293.0	298.2	303.0	313.2	328.2	348.2
1000/T / K^{-1}	4.0	3.8	3.5	3.4	3.4	3.3	3.2	3.0	2.9
Calc.									
C1[a]	767.7	779.2	792.9	794.4	795.8	796.7	797.9	797.8	794.9
C3	224.9	226.4	228.1	228.3	228.5	228.6	228.8	228.7	228.4
C2[a]	–68.4	–73.0	–78.5	–79.2	–79.7	–80.1	–80.6	–80.5	–79.3
C4[a]	182.6	183.3	184.1	184.2	184.3	184.4	184.5	184.5	184.3
C5	184.8	185.6	186.6	186.7	186.8	186.9	187.0	187.0	186.8
C6	182.2	182.3	182.5	182.6	182.6	182.6	182.6	182.6	182.6
C7	54.8	54.8	54.9	54.9	54.9	54.9	54.9	54.9	54.9
MAD[b]	4.2	6.0	4.3	3.0	5.6	2.9	3.9	5.6	3.4

[a] Average of two sites in the model. [b] Calculated for C1, C2 and C3 of HKUST-1.

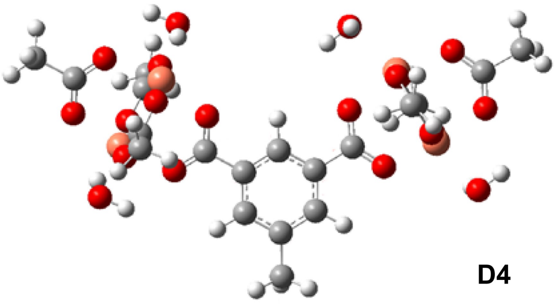

D4

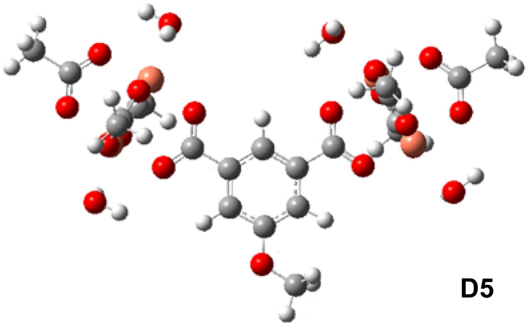

D5

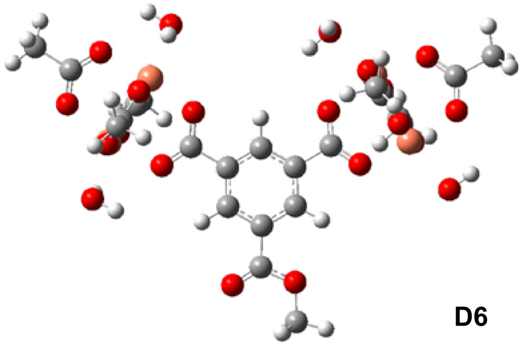

D6

Fig. S9. GFN2-xTB optimised structures of models **D4**-**D6** (see **Fig. 2** and **Table 1**). Atoms are coloured brown = Cu, red = O, grey = C and white = H.

Table S11. Bond distances (in Å) of optimised geometries for model **D1** in the Q(1) state, and changes in bond lengths for different spin configurations (see **Fig. S3**) at PBE0-D3/AE1 level.

	Q(1)	S(1)	S(5)	T(1)	T(3)	Q(1)[a]
Cu-Cu (Averaged)	2.4619	0.0051	–0.0009	0.0016	0.0017	0.1462
Cu-O (Averaged)	1.9520	–0.0002	0.0001	0.0000	0.0000	0.0416

[a] Optimised at GFN2-xTB level.

Table S12. Shieldings (in ppm) of carbon sites in each spin configuration of model **D1** at 298.2 K, and the scaled energy differences ΔE with respect to the lowest configuration, Singlet(1) (s = 1.305) (CAM-B3LYP/II//GFN2-xTB).

Site	$\sigma_{Q(1)}$	$\sigma_{S(1)-S(6)}$	$\sigma_{T(1)}$	$\sigma_{T(2)}$	$\sigma_{T(3)}$	$\sigma_{T(4)}$
C1[a]	–3086.2	44.3	–969.4	–1030.3	–1028.4	–968.5
C3	–361.8	48.3	64.8	–240.2	–238.0	64.9
C5	–188.1	41.1	–108.4	34.7	36.9	–105.7
C4[a]	–148.3	43.4	–87.6	48.8	48.9	–86.8
C6	141.2	164.9	148.4	166.7	166.6	147.7
C2[a]	1358.7	3.8	463.8	445.4	449.3	462.9
ΔE / cm^{-1}	572.34	[b]	286.00	286.65	286.61	285.98

[a] Average of two sites in the model. [b] Singlet(1) - Singlet(6): 0.00, 0.00, 0.72, 0.58, 571.90, 571.88.

Table S13. Shieldings (in ppm) of carbon sites in each spin configuration of model **D3** at 298.2 K, and the scaled energy differences ΔE with respect to the lowest configuration, Singlet(2) (s = 1.330) (CAM-B3LYP/II//GFN2-xTB).

Site	$\sigma_{Q(1)}$	$\sigma_{S(1)-S(6)}$	$\sigma_{T(1)}$	$\sigma_{T(2)}$	$\sigma_{T(3)}$	$\sigma_{T(4)}$
C1	–3179.4	44.2	–40.1	–31.0	–2097.4	–1952.8
C1'	–3148.2	44.6	–1931.6	–2077.2	–30.3	–39.3
C3	–364.0	40.9	57.9	–240.0	–245.5	56.5
C5	–187.0	48.9	–102.9	45.0	39.0	–102.9
C4	–150.2	42.0	–95.5	–94.1	199.3	–91.9
C4'	–148.0	40.7	–103.8	210.2	–94.9	–94.1
C2	1288.4	5.0	10.3	–27.7	857.8	890.3
C2'	1300.2	4.8	898.8	866.0	1.7	–20.7
C6	–29.7	13.5	–17.6	18.8	15.0	–17.8
C7	131.7	132.8	132.2	133.9	132.0	132.2
ΔE / cm^{-1}	582.41	[a]	291.22	291.80	291.51	290.97

[a] Singlet(1) - Singlet(6): 0.07, 0.00, 0.62, 0.57, 582.08, 582.05.

Table S14. Shieldings (in ppm) of carbon sites in each spin configuration of model **D4** at 298 K, and the scaled ΔE with respect to Singlet(1) (s = 1.374) (CAM-B3LYP/II//GFN2-xTB).

Site	$\sigma_{Q(1)}$	$\sigma_{S(1)\text{-}S(6)}$	$\sigma_{T(1)}$	$\sigma_{T(2)}$	$\sigma_{T(3)}$	$\sigma_{T(4)}$
C1	–2847.5	43.1	–105.2	34.0	–1872.9	–1727.2
C1'	–2781.9	43.3	–1708.3	–1835.4	–170.3	130.8
C3	–311.9	48.8	54.1	–222.3	–219.1	96.3
C5	–149.1	41.2	–83.8	45.2	33.4	–75.0
C4	–101	45.9	–76.4	189.6	–64.9	–65
C4'	–98.8	43.9	–57.5	–60.4	152.3	–53.9
C2	1057.4	4.0	–105.9	85.4	703.6	733.9
C2'	1004.9	5.5	696.7	670.3	125.4	–142.2
C6	144.0	164.8	150.2	167.1	164.6	148.2
ΔE / cm^{-1}	514.57	[a]	256.50	257.17	258.21	257.67

[a] Singlet(1) - Singlet(6): 0.00, –, 0.51, –, 514.18, – (where – denotes omitted value).

Table S15. Calculated ^{13}C shifts (in ppm) at different temperatures using model **D4** with s = 1.374 for ΔE_{ST} of ~187 cm^{-1} (CAM-B3LYP/II//GFN2-xTB), using **Eq. S15**. Experimental shifts of STAM-17-Me are given for comparison.

T / K	278	298	318	338
1000/T / K^{-1}	3.60	3.36	3.14	2.96
Calc.				
C1[a]	803.0	801.9	797.1	789.7
C3	222.5	222.4	221.7	220.8
C5	189.3	189.3	189.0	188.5
C4[a]	176.5	176.4	176.2	175.8
C6	27.6	27.6	27.6	27.5
C2[a]	–53.8	–53.4	–51.7	–49.0
Exp.[S2] STAM-17-Me				
C1	/	770.0	/	/
C3	222.5	221.3	219.8	218.0
C4, C5[b]	179.7	179.3	178.7	177.9
C6	25.0	24.8	24.7	24.5
C2	–61.4	–60.3	–55.8	–49.6
MAD	4.6	4.7 (9.3)[c]	4.4	3.8

[a] Average of two sites in the model. [b] These signals were overlapped at all temperatures in the experimental dataset. [c] the MAD in brackets includes C1 at 298 K.

**Table S16.** ZFS corrected ^{13}C shifts (in ppm) for model **D4** with s = 1.374 for ΔE_{ST} of ~187 cm^{-1} (CAM-B3LYP(BLYP)/II//GFN2-xTB), using **Eq. S15**.				
T / K	278	298	318	338
1000/T / K^{-1}	3.60	3.36	3.14	2.96
C1[a]	801.9	800.9	796.2	788.9
C3	222.3	222.2	221.5	220.6
C5	189.0	189.0	188.7	188.3
C4[a]	175.8	175.8	175.6	175.2
C6	27.4	27.4	27.4	27.3
C2[a]	–54.8	–54.3	–52.5	–49.8

[a] Average of two sites in the model.

Table S17. Shieldings (in ppm) of carbon sites in each spin configuration at 298 K and the scaled energy differences ΔE with respect to the lowest configuration, Singlet(1) (s = 1.306) for model **D5** (CAM-B3LYP/II//GFN2-xTB).

Site	$\sigma_{Q(1)}$	$\sigma_{S(1)-S(6)}$	$\sigma_{T(1)}$	$\sigma_{T(2)}$	$\sigma_{T(3)}$	$\sigma_{T(4)}$
C1	–2867.1	41.6	–200.2	117.4	–1881.9	–1733.5
C1'	–2807.9	42.3	–1732.2	–1852.5	–136.6	104.6
C3	–277.8	54.7	37.6	–182.4	–196.2	107.8
C5	–245.8	18.4	–154.2	39.9	–2.7	–149.9
C4	–108.7	56.6	–99.2	241.4	–75.3	–69.6
C4'	–93.2	65.7	–49.6	–55.9	189.5	–41.5
C2	1017.2	4.6	11.1	–33.9	678.0	708.3
C2'	984.5	5.7	685.2	657.0	104.5	–124.7
C6	124.9	130.3	126.9	134.1	126.9	126.4
ΔE / cm^{-1}	489.08	[a]	243.16	243.79	243.16	245.04

[a] Singlet(1) – Singlet(6):0.00, –, 0.46, –, 488.71, – (where – denotes omitted value).

Table S18. Calculated ^{13}C shifts (in ppm) at different temperatures using model **D5** with s = 1.306 for ΔE_{ST} of ~186 cm^{-1} (CAM-B3LYP/II//GFN2-xTB), using **Eq. S15**. Experimental shifts of STAM-17-OMe are also given.[S2]

T / K	278	298	318	338
1000/T / K^{-1}	3.60	3.36	3.14	2.96
Calc.				
C1[a]	843.6	839.1	831.2	820.9
C5	232.0	231.6	231.0	230.1
C3	214.5	213.9	213.0	211.8
C4[a]	166.6	166.3	165.9	165.2
C6	58.5	58.4	58.4	58.4
C2[a]	–59.0	–57.4	–54.7	–51.2
Exp. STAM-17-OMe				
C1	/	/	/	/
C5	219.2	218.7	217.9	216.8
C3	217.7	216.4	214.5	212.9
C4	166.0	165.7	165.0	164.1
C6	49.9	50.0	50.3	50.5
C2	–60.3	–57.4	–52.5	–48.3
MAD	5.3	4.9	5.2	5.3

[a] Average of two sites in the model.

Table S19. Shieldings (in ppm) of the carbon sites in each spin configuration at 298 K and the scaled ΔE with respect to Singlet(1) (s = 1.288) for model **D6** (CAM-B3LYP/II//GFN2-xTB).

Site	$\sigma_{Q(1)}$	$\sigma_{S(1)\text{-}S(6)}$	$\sigma_{T(1)}$	$\sigma_{T(2)}$	$\sigma_{T(3)}$	$\sigma_{T(4)}$
C1	–2869.7	43.7	–198.5	133.3	–1889.8	–1747.9
C1'	–2820.2	43.2	–1749.5	–1859.4	–194.0	164.7
C3	–294.6	41.4	18.6	–191.5	–214.8	104.0
C5	–157.4	49.4	–81.3	61.2	26.4	–80.5
C4	–130.9	43.0	–112.1	219.8	–82.7	–85.4
C4'	–87.4	42.7	–53.4	–52.4	159.0	–56.3
C2	963.5	6.2	40.1	–54.7	642.3	670.3
C2'	970.0	6.1	671.5	645.4	128.9	–139.3
C6	–25.1	12.8	–14.6	19.9	10.7	–16.3
C7	131.7	132.9	132.2	134.0	132.0	132.2
ΔE / cm^{-1}	481.4	[a]	239.7	240.4	241.9	241.1

[a] Singlet(1) – Singlet(6): 0.00, –, 0.4, –, 481.1, – (where – denotes omitted value).

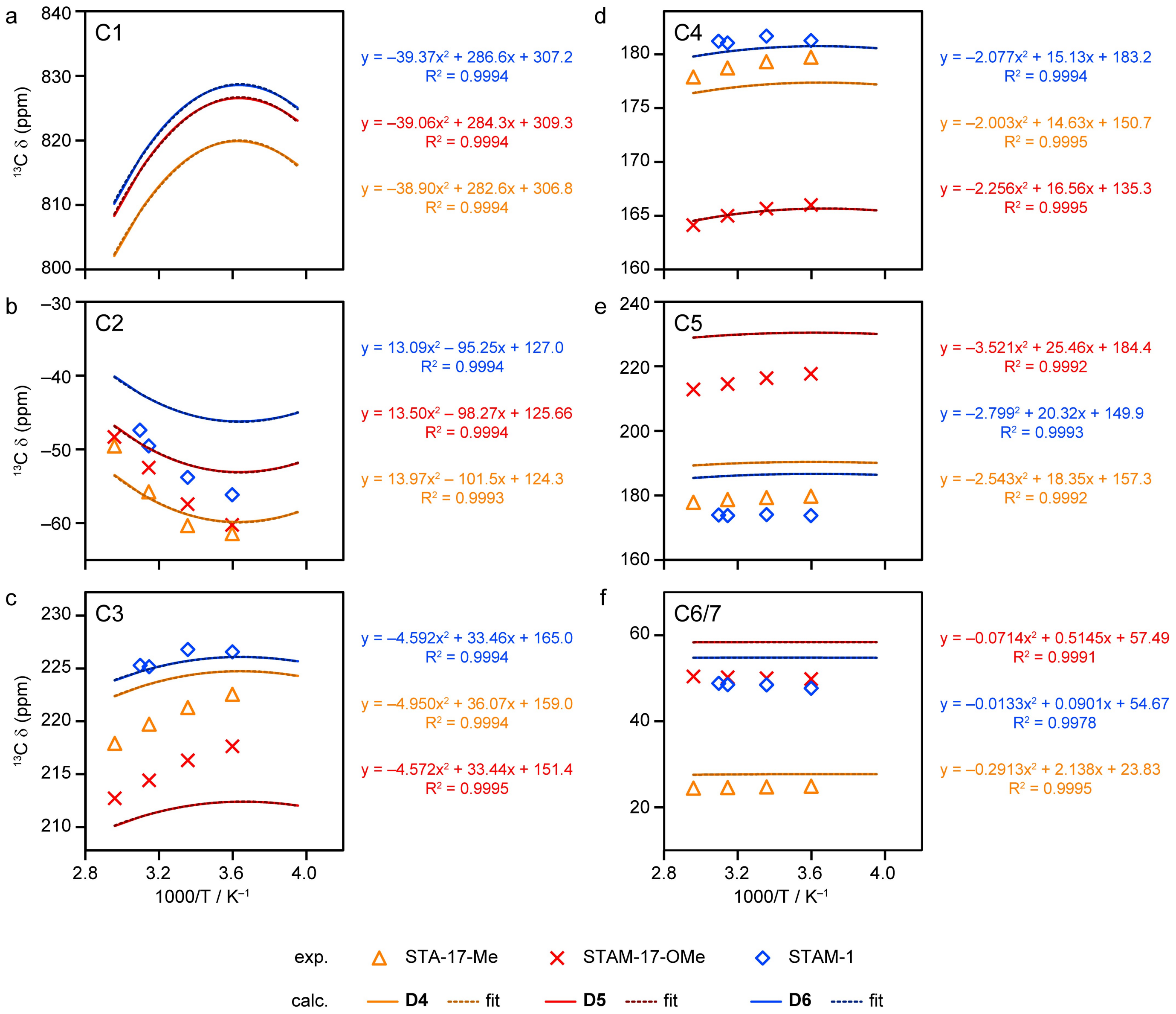

Fig. S10. Calculated VT plots (solid lines) for substitution effects on ^{13}C shifts of models **D4** - **D6**, with s = 1.340 (averaged value from the best-fit values for **D4** and **D5**, CAM-B3LYP/**II**//GFN2-xTB), using **Eq. S15**. Experimental shifts (individual data points) are taken from reference S2 and S3. (a) C1 (no experimental data), (b) C2, (c) C3, (d) C4, (e), C5 and (f) C6 in STAM-17-Me and STAM-17-OMe, C7 in STAM-1 (the C6 carboxylate site in STAM-1 has no analogue in STAM-17-Me/OMe, so is not included in this comparison). Note that C4 and C5 in STAM-17-Me were not resolved experimentally and the gold points in (d) and (e) are the same. Second-order polynomial equations (dashed lines) have been fitted to the calculated values to show that the trends in the shifts with temperature are similar for these models. Note that the polynomial fit is only applied over the temperature range 253 to 323 K (3.10 to 3.95 K^{-1}).

Table S20. Calculated ^{13}C shifts (in ppm) at different temperatures for hydrated STAM-1 and HKUST-1, using model **D6** with s = 1.295 and 1.288, respectively, for ΔE_{ST} of ~187 cm^{-1}, respectively (CAM-B3LYP/II//GFN2-xTB), using **Eq. S15**. Experimental values for both MOFs are also shown for comparison.[S3]

Exp. hydrated HKUST-1[a]

T / K^{-1}	250†	258	263†	263	268	273	278	283	293†	298	303†	313	323
1000/T 1/K^{-1}	4.0	3.9	3.8	3.8	3.7	3.7	3.6	3.5	3.4	3.4	3.3	3.2	3.1
C1	870.8	868.2	863.4	865.4	865.0	/	858.6	/	851.8	848.2	845.8	843.1	838.1
C3	230.3	229.1	229.6	228.7	228.5	228.5	228.2	228.1	227.8	227.4	227.2	226.7	226.4
C2	–58.7	–54.8	–57.3	–54.5	–54.2	–52.9	–52.7	–50.3	–50.5	–50.5	–49.2	–50.8	–43.2

Calc. hydrated HKUST-1

T / K^{-1}	250	258	263	268	273	278	283	288	293	298	303	308	313	318	323
1000/T 1/K^{-1}	4.0	3.9	3.8	3.7	3.7	3.6	3.5	3.5	3.4	3.4	3.3	3.2	3.2	3.1	3.1
C1[b]	854.6	855.9	856.2	856.1	855.7	855.0	854.0	852.7	851.2	849.5	847.5	845.4	843.1	840.7	838.1
C3	229.0	229.2	229.2	229.2	229.1	229.1	228.9	228.8	228.6	228.4	228.2	227.9	227.7	227.4	227.1
C2[b]	–54.9	–55.3	–55.4	–55.4	–55.3	–55.0	–54.7	–54.3	–53.8	–53.2	–52.5	–51.8	–51.1	–50.3	–49.4
C4[b]	182.1	182.2	182.2	182.2	182.2	182.1	182.1	182.0	181.9	181.8	181.7	181.6	181.5	181.4	181.2
C5	188.5	188.6	188.7	188.7	188.6	188.6	188.5	188.4	188.3	188.2	188.1	187.9	187.8	187.6	187.4
C6	184.2	184.2	184.2	184.2	184.2	184.2	184.1	184.1	184.1	184.1	184.1	184.0	184.0	184.0	183.9
C7	54.8	54.8	54.8	54.8	54.8	54.8	54.8	54.8	54.8	54.8	54.8	54.8	54.8	54.8	54.8
MAD[c]	{7.1}	4.3	3.5 {3.2}	3.6	1.5	2.3	2.6	/	{1.6}	1.7	{2.0}	/	0.4	/	2.3

Exp. hydrated STAM-1[a]

T / K^{-1}	250†	258	263†	263	268	273	278	283	293†	298	303†	313	318	323
1000/T 1/K^{-1}	4.0	3.9	3.8	3.8	3.7	3.7	3.6	3.5	3.4	3.3	3.3	3.2	3.1	3.1
C2	–59.1	–56.7	–58.1	–58.7	–56.4	–56.4	–56.0	–54.9	–54.2	–53.2	–53.1	–49.2	–49.4	–47.4
C3	226.0	227.1	226.7	226.8	226.7	226.7	226.5	226.5	226.3	226.2	225.6	225.5	225.1	225.2
C4	180.6	181.6	181.4	181.3	181.3	181.4	181.4	181.5	181.4	181.3	181.2	181.1	181.1	181.3
C5	173.3	173.7	173.9	173.4	173.6	173.7	173.7	173.8	174.1	173.8	173.8	173.8	173.8	174.0
C6	177.3	176.7	178.2	176.0	176.3	176.3	176.1	176.5	178.6	177.1	178.7	177.3	177.4	177.6
C7	48.1	47.8	48.6	47.2	47.5	47.6	47.6	47.8	49.1	48.0	49.0	48.4	48.6	48.9

Calc. hydrated STAM-1 (**D6**)

T / K^{-1}	250	258	263	268	273	278	283	288	293	298	303	308	313	318	323
1000/T 1/K^{-1}	4.0	3.9	3.8	3.7	3.7	3.6	3.5	3.5	3.4	3.4	3.3	3.2	3.2	3.1	3.1
C1[b]	850.8	852.3	852.7	852.7	852.4	851.7	850.8	849.6	848.2	846.5	844.7	842.6	840.4	838.0	835.5
C3	228.6	228.8	228.8	228.8	228.8	228.7	228.6	228.4	228.3	228.1	227.8	227.6	227.3	227.1	226.8
C2[b]	–53.6	–54.1	–54.3	–54.3	–54.2	–53.9	–53.6	–53.2	–52.8	–52.2	–51.6	–50.9	–50.2	–49.4	–48.6
C4[b]	181.9	182.0	182.0	182.0	182.0	182.0	181.9	181.9	181.8	181.7	181.6	181.5	181.4	181.2	181.1
C5	188.3	188.4	188.4	188.4	188.4	188.4	188.3	188.2	188.1	188.0	187.9	187.7	187.6	187.4	187.2
C6	184.1	184.1	184.1	184.1	184.1	184.1	184.1	184.1	184.1	184.0	184.0	184.0	184.0	183.9	183.9
C7	54.8	54.8	54.8	54.8	54.8	54.8	54.8	54.8	54.8	54.8	54.8	54.8	54.8	54.8	54.8
MAD[c]	{6.3}	5.6	6.3 {5.5}	5.8	5.8	5.8	5.5	/	{4.9}	5.2	{4.9}	/	5.0	4.7	4.7

[a] Data taken from a mix of two series of experiments in both shim temperature controlled and no shim temperature control (see ref. S3 for details), † = shim temperature not controlled. [b] Average of two sites in the model. [c] Calculated for the carbon sites available in the MOFs (values in curly brackets are calculated for another experimental measurement with no shim temperature control).

S4. Effects of hydration on aromatic resonances

Analysis of the experimental NMR data for STAM MOFs is complicated by the fact that the degree of hydration is not always clear, *i.e.*, if indeed all Cu sites are saturated with water ligands, in particular the hydrated STAM-1. Models with varying numbers of coordinated water molecules were explored computationally to study the effect of the extent of hydration. Due to the asymmetry of the CO_2Me substituent, there are four non-equivalent water coordination sites. For simplicity, hydrated derivatives of model **D1** are used here to reduce the number of models that need to be constructed (where there are two pairs of non-equivalent sites). This simplification will not significantly change the variation of shifts with temperature according to the results shown in **Fig. S10**.

Structures considered for loading with 1 - 5 water molecules are displayed in **Fig. S11**. Structure 4 is the hydrated model considered so far, structures 1-X to 3-X were constructed from this by deleting the appropriate number of water molecules at the different possible locations. These are models for partially hydrated MOFs. Structure 5 was obtained by adding an additional water molecule interacting simultaneously with two coordinated water ligands (at the "top" in the orientation of **Fig. S11**). This model is designed to include specific interactions with excess water in the pores that are expected to be present in fully hydrated MOFs.

Models with the same number of coordinating water molecules have similar relative energies (difference within 1 kcal/mol), with the exception of structure 2-1, which is 1.66 kcal/mol higher in energy than the most stable isomer ($\Delta\Delta E$ values in **Table S21**). The dissociation of water molecules from the di-dimer model is an endothermic process (see the positive ΔH values in **Table S21** for the removal of water molecules from structure 4 to the structure specified). The as-calculated Gibbs free energies for water dissociation are negative at room temperature (ΔG values in **Table S21**), which implies that the ligands are unbound, this result is likely to be a consequence of the way the entropies are computed from standard statistical dynamics expressions (*i.e.*, the ideal gas approximation). While, in particular, the calculated translational entropies would be appropriate for the gas phase, they are likely to be overestimated compared to the situation in condensed phases, where the molar volume available to each particle is much smaller. This small molar volume will affect the entropic driving forces for

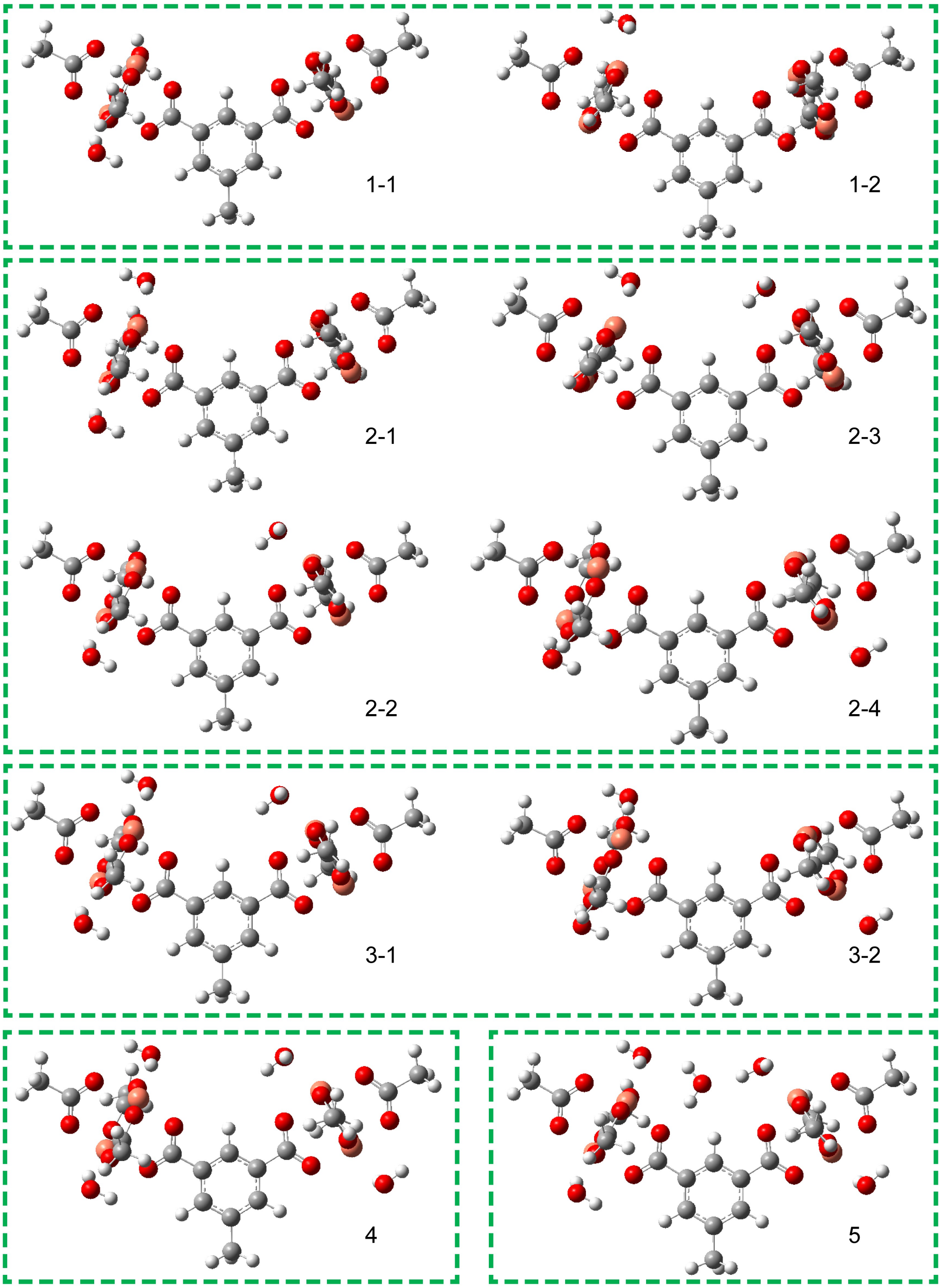

Fig. S11. Configurations (optimised with GFN2-xTB) of **D1**-derived models loaded with different numbers of water molecules. Configuration 4 is the same as model **D4**.

association/dissociation processes and effectively reduce the absolute value of ΔS. As a workaround Martin *et al.* have suggested[S4] to use an elevated pressure in the evaluation of the thermodynamic corrections (one where an ideal gas of the ligand, water, would have the same density as the liquid), in order to model binding energies of water ligands

in aqueous metal ion complexes. This would serve to reduce absolute TΔS contributions by *ca*. 4 kcal/mol per particle at 298.15 K (*i.e.*, increase the ΔG values for the dissociative processes in **Table S21** such that they would all become positive). Because such a "pressure correction" is somewhat ill-defined for modelling the situation in a pore inside the MOF, this was not applied here but note that the (small) negative ΔG values in **Table S21** are likely to be an artefact of the approximations involved and that all water ligands will actually be bound in the temperature range of interest.

For pNMR calculations, models with the same number of water molecules are grouped together, and their resonances averaged based on their relative energies, ΔΔE in **Table S21**, using a Boltzmann distribution at each temperature. The resulting VT trends in **Fig. S12** in these models do show some variations with the different water loading, especially for the most de/shielded sites (**Fig. S12A** and **B**). The experimental data set for the sample of STAM-1 with the unclear extent of hydration (diamonds in **Fig. S12**) seems to show trends with temperature that would be broadly compatible with those of the models loaded with 4 and 5 water molecules (*i.e.*, the coefficients for x^2 in these fitted polynomial equations are close). However, it would be difficult to draw any conclusions from these results regarding the extent of hydration in the experimental sample (note that here we concentrate on the temperature dependence, the chemical shifts themselves cannot be compared directly because of the different substituents, Me vs. CO_2Me, see discussion in the main paper).

Table S21. Water dissociation energies of di-dimer models loaded with different number of water molecules (**Fig. S11**) for the process of 4 → A + nH_2O and 5 → 4 + H_2O (at the CAM-B3LYP(D3)/II//GFN2-xTB level).[a]

Models	1-1	1-2	2-1	2-2	2-3	2-4	3-1	3-2	5
ΔE (ΔΔE [b]) / kcal/mol	31.67 (0.48)	31.34 (0)	21.56 (1.66)	20.29 (0.50)	19.88 (0)	20.82 (0.85)	9.97 (0)	10.78 (0.58)	13.30 [c]
ΔH / kcal/mol	26.61	26.27	18.18	16.91	16.50	17.46	8.27	9.11	11.72 [c]
ΔG (298.15 K) / kcal/mol	–1.24	–1.95	–0.62	–2.02	–2.20	–0.98	–1.33	–0.19	0.99 [c]

[a] Describing the water dissociation process from structure 4 in **Fig. S11** (Model **D4**) to one of the other models. Calculated values are corrected for zero-point energy (ZPE) and basis set superposition error (BSSE). [b] The raw ΔΔE relative to the most stable isomer for the same water dissociation process. [c] Describing the water dissociation process from structure 5 to 4.

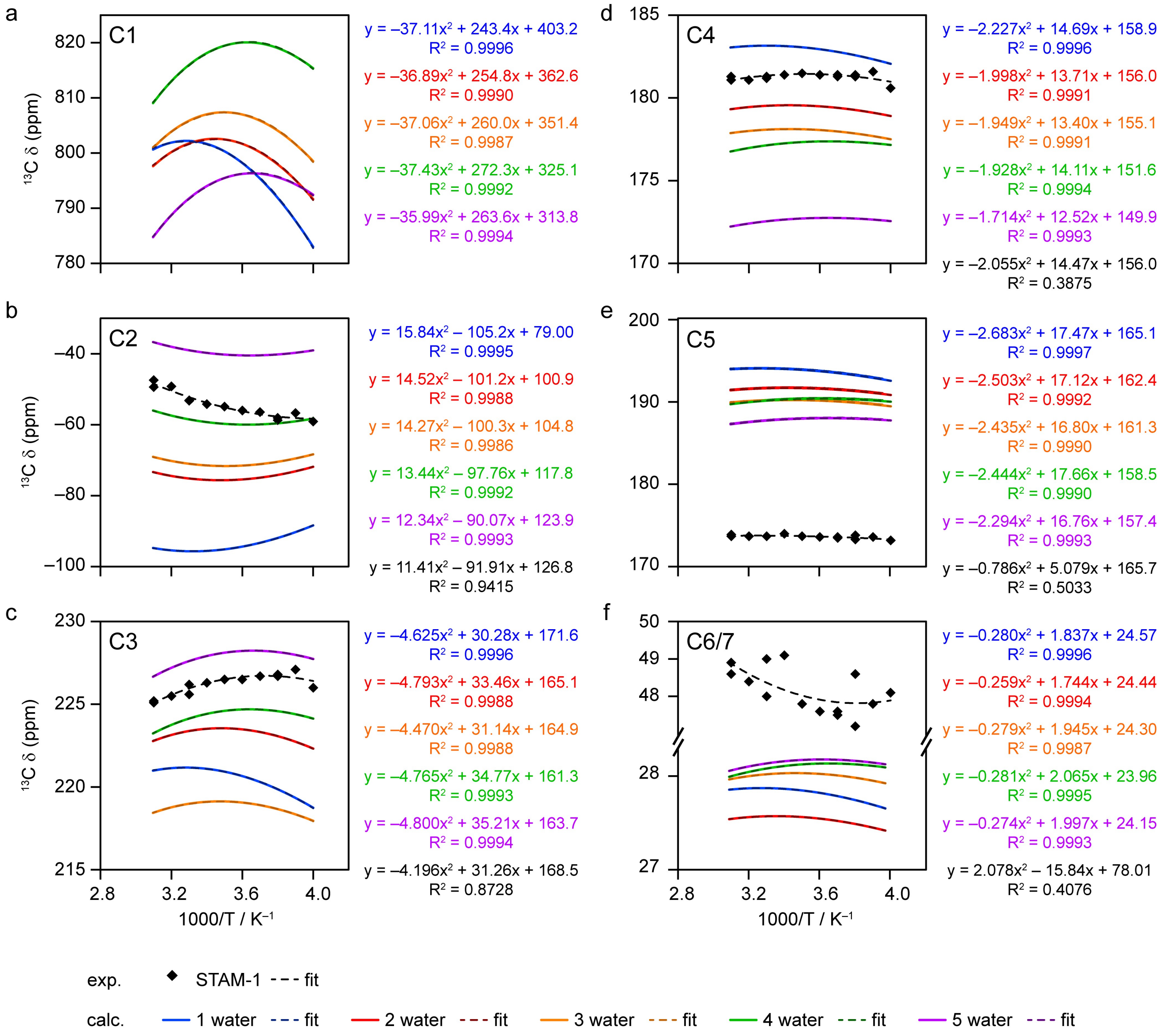

Fig. S12. Plots showing shift variation with temperature for **D1**-based models loaded with different numbers of water molecules, with s = 1.340 (from the best fit for hydrated STAM-17 series using models **D4** and **D5**), using **Eq. S15** (CAM-B3LYP/II//GFN2-xTB). (a) C1 (no experimental data available), (b) C2, (c) C3, (d) C4, (e) C5, (f) C6 in the model (containing **L3**) and C7 in STAM-1 (containing **L2**). Experimental VT NMR data are for the STAM-1 sample with an unknown extent of hydration.[S3] Shifts for the same site for the models with the same number of water molecules models are grouped, and then Boltzmann averaged based on their relative energies (ΔΔE in **Table S21**).

S5. Models with three copper paddlewheel dimers

Radial tri-dimer model for activated HKUST-1

Arguably, a tri-dimer model for HKUST-1, such as the radial tri-dimer model **Ra1** in **Fig. S13**, would be more realistic than the di-dimer models discussed above, as it would allow the spin systems of three dimer units to communicate *via* the linker. For six spin centres in a plane, 32 unique configurations (excluding mirror images with all six spins flipped simultaneously) can be constructed: one septet, six quintet, 15 triplet and 10 singlet spin configurations. Adding four singlet spin configurations where six spins are flipped simultaneously, having antiferromagnetic coupling in the three dimers, the total number of the configurations is 36. Owing to the C_3 symmetry (exact or approximate) of this model, many of these configurations are equivalent or near-equivalent, and only the 14 unique configurations are shown in **Fig. S13** which include one septet, two quintet, five triplet and six singlet spin configurations.

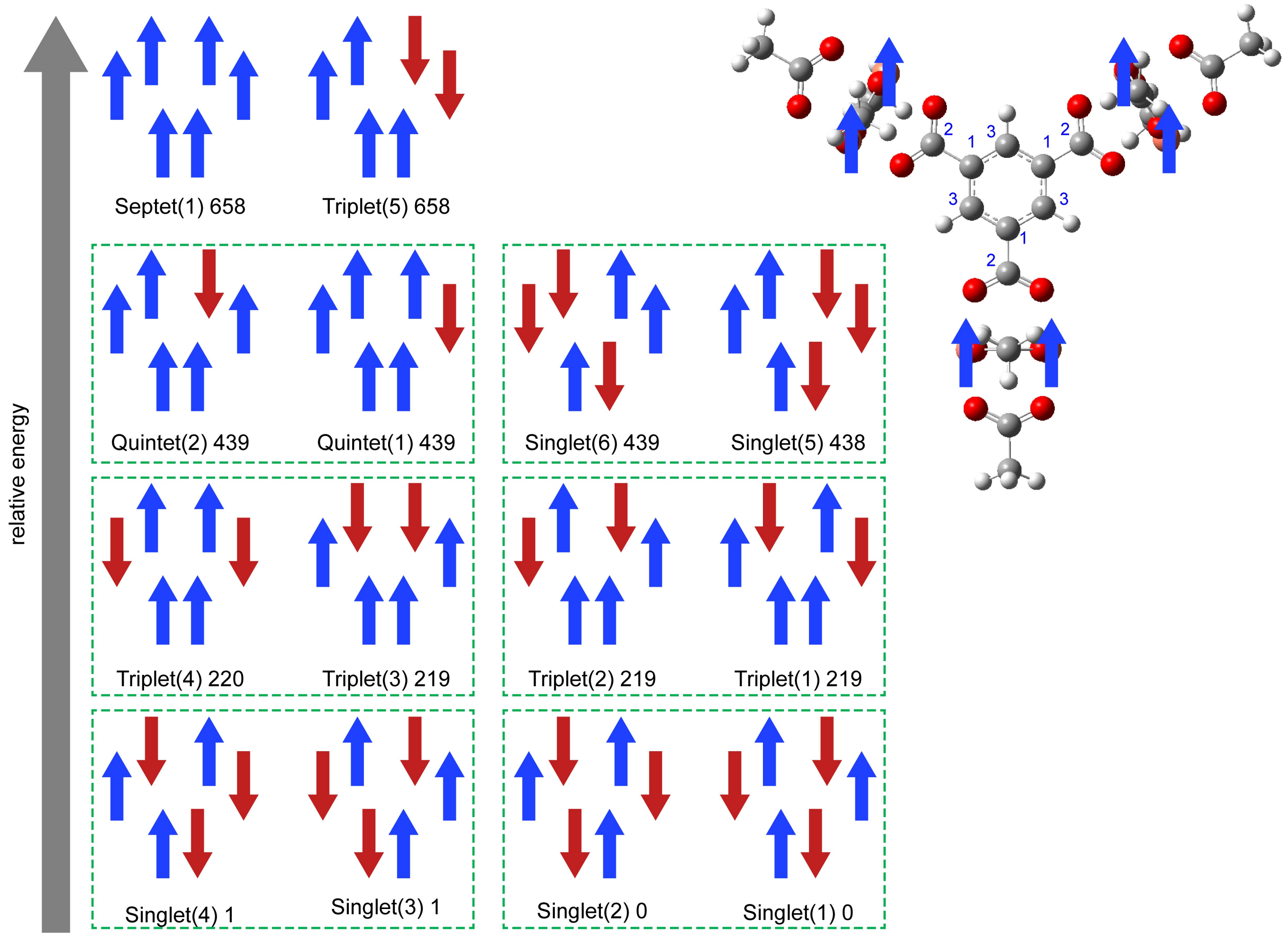

Fig. S13. Spin configurations of model **Ra1** (see **Fig. 2** and **Table 1**). All nomenclature and colouring schemes are as in **Fig. S1**.

Expressions for calculating ^{13}C shifts for tri-dimer models

The ways of calculating the total shielding for a carbon site can vary, depending on which spin configurations are assumed to combine into distinct states. Firstly, assuming all configurations are distinct states, the total shielding for a site is calculated as

$$\sigma_{\mathrm{total}} = [f_{S(1)}\,\sigma_{S(1)} + f_{S(2)}\,\sigma_{S(2)} + f_{S(3)}\,\sigma_{S(3)} + f_{S(3\text{-}2)}\,\sigma_{S(3\text{-}2)} + f_{S(3\text{-}3)}\,\sigma_{S(3\text{-}3)} + f_{S(4)}\,\sigma_{S(4)} + f_{S(4\text{-}2)}\,\sigma_{S(4\text{-}2)} + f_{S(4\text{-}3)}\,\sigma_{S(4\text{-}3)} + f_{S(5)}\,\sigma_{S(5)} + f_{S(5\text{-}2)}\,\sigma_{S(5\text{-}2)} + f_{S(5\text{-}3)}\,\sigma_{S(5\text{-}3)} + f_{S(6)}\,\sigma_{S(6)} + f_{S(6\text{-}2)}\,\sigma_{S(6\text{-}2)} + f_{S(6\text{-}3)}\,\sigma_{S(6\text{-}3)} + f_{T(1)}\,\sigma_{T(1)} + f_{T(1\text{-}2)}\,\sigma_{T(1\text{-}2)} + f_{T(1\text{-}3)}\,\sigma_{T(1\text{-}3)} + f_{T(2)}\,\sigma_{T(2)} + f_{T(2\text{-}2)}\,\sigma_{T(2\text{-}2)} + f_{T(2\text{-}3)}\,\sigma_{T(2\text{-}3)} + f_{T(3)}\,\sigma_{T(3)} + f_{T(3\text{-}2)}\,\sigma_{T(3\text{-}2)} + f_{T(3\text{-}3)}\,\sigma_{T(3\text{-}3)} + f_{T(4)}\,\sigma_{T(4)} + f_{T(4\text{-}2)}\,\sigma_{T(4\text{-}2)} + f_{T(4\text{-}3)}\,\sigma_{T(4\text{-}3)} + f_{T(5)}\,\sigma_{T(5)} + f_{T(5\text{-}2)}\,\sigma_{T(5\text{-}2)} + f_{T(5\text{-}3)}\,\sigma_{T(5\text{-}3)} + f_{Q(1)}\,\sigma_{Q(1)} + f_{Q(1\text{-}2)}\,\sigma_{Q(1\text{-}2)} + f_{Q(1\text{-}3)}\,\sigma_{Q(1\text{-}3)} + f_{Q(2)}\,\sigma_{Q(2)} + f_{Q(2\text{-}2)}\,\sigma_{Q(2\text{-}2)} + f_{Q(2\text{-}3)}\,\sigma_{Q(2\text{-}3)} + f_{Se(1)}\,\sigma_{Se(1)}] \,/\, [f_{S(1)} + f_{S(2)} + f_{S(3)} + f_{S(3\text{-}2)} + f_{S(3\text{-}3)} + f_{S(4)} + f_{S(4\text{-}2)} + f_{S(4\text{-}3)} + f_{S(5)} + f_{S(5\text{-}2)} + f_{S(5\text{-}3)} + f_{S(6)} + f_{S(6\text{-}2)} + f_{S(6\text{-}3)} + f_{T(1)} + f_{T(1\text{-}2)} + f_{T(1\text{-}3)} + f_{T(2)} + f_{T(2\text{-}2)} + f_{T(2\text{-}3)} + f_{T(3)} + f_{T(3\text{-}2)} + f_{T(3\text{-}3)} + f_{T(4)} + f_{T(4\text{-}2)} + f_{T(4\text{-}3)} + f_{T(5)} + f_{T(5\text{-}2)} + f_{T(5\text{-}3)} + f_{Q(1)} + f_{Q(1\text{-}2)} + f_{Q(1\text{-}3)} + f_{Q(2)} + f_{Q(2\text{-}2)} + f_{Q(2\text{-}3)} + f_{Se(1)}]\ .$$

Eq. S16

Note that all possible singlet states are included explicitly here. All are assumed to have the same set of shieldings (σ_S in **Table S22**) but their Boltzmann factors may differ. Note also that for generality, no symmetry equivalence is assumed. Next, some configurations are combined into individual electronic states. If the four lowest singlet configurations are grouped pairwise the resulting expression is

$$\sigma_{\mathrm{total}} = [\tfrac{1}{2}(f_{S(1)} + f_{S(2)})\,\tfrac{1}{2}(\sigma_{S(1)} + \sigma_{S(2)}) + \tfrac{1}{2}(f_{S(3)} + f_{S(4)})\,\tfrac{1}{2}(\sigma_{S(3)} + \sigma_{S(4)}) + \tfrac{1}{2}(f_{S(3\text{-}2)} + f_{S(4\text{-}2)})\,\tfrac{1}{2}(\sigma_{S(3\text{-}2)} + \sigma_{S(4\text{-}2)}) + \tfrac{1}{2}(f_{S(3\text{-}3)} + f_{S(4\text{-}3)})\,\tfrac{1}{2}(\sigma_{S(3\text{-}3)} + \sigma_{S(4\text{-}3)}) + f_{S(5)}\,\sigma_{S(5)} + f_{S(5\text{-}2)}\,\sigma_{S(5\text{-}2)} + f_{S(5\text{-}3)}\,\sigma_{S(5\text{-}3)} + f_{S(6)}\,\sigma_{S(6)} + f_{S(6\text{-}2)}\,\sigma_{S(6\text{-}2)} + f_{S(6\text{-}3)}\,\sigma_{S(6\text{-}3)} + f_{T(1)}\,\sigma_{T(1)} + f_{T(1\text{-}2)}\,\sigma_{T(1\text{-}2)} + f_{T(1\text{-}3)}\,\sigma_{T(1\text{-}3)} + f_{T(2)}\,\sigma_{T(2)} + f_{T(2\text{-}2)}\,\sigma_{T(2\text{-}2)} + f_{T(2\text{-}3)}\,\sigma_{T(2\text{-}3)} + f_{T(3)}\,\sigma_{T(3)} + f_{T(3\text{-}2)}\,\sigma_{T(3\text{-}2)} + f_{T(3\text{-}3)}\,\sigma_{T(3\text{-}3)} + f_{T(4)}\,\sigma_{T(4)} + f_{T(4\text{-}2)}\,\sigma_{T(4\text{-}2)} + f_{T(4\text{-}3)}\,\sigma_{T(4\text{-}3)} + f_{T(5)}\,\sigma_{T(5)} + f_{T(5\text{-}2)}\,\sigma_{T(5\text{-}2)} + f_{T(5\text{-}3)}\,\sigma_{T(5\text{-}3)} + f_{Q(1)}\,\sigma_{Q(1)} + f_{Q(1\text{-}2)}\,\sigma_{Q(1\text{-}2)} + f_{Q(1\text{-}3)}\,\sigma_{Q(1\text{-}3)} + f_{Q(2)}\,\sigma_{Q(2)} + f_{Q(2\text{-}2)}\,\sigma_{Q(2\text{-}2)} + f_{Q(2\text{-}3)}\,\sigma_{Q(2\text{-}3)} + f_{Se(1)}\,\sigma_{Se(1)}] \,/\, [\tfrac{1}{2}(f_{S(1)} + f_{S(2)}) + \tfrac{1}{2}(f_{S(3)} + f_{S(4)}) + \tfrac{1}{2}(f_{S(3\text{-}2)} + f_{S(4\text{-}2)}) + \tfrac{1}{2}(f_{S(3\text{-}3)} + f_{S(4\text{-}3)}) + f_{S(5)} + f_{S(5\text{-}2)} + f_{S(5\text{-}3)} + f_{S(6)} + f_{S(6\text{-}2)} + f_{S(6\text{-}3)} + f_{T(1)} + f_{T(1\text{-}2)} + f_{T(1\text{-}3)} + f_{T(2)} + f_{T(2\text{-}2)} + f_{T(2\text{-}3)} + f_{T(3)} + f_{T(3\text{-}2)} + f_{T(3\text{-}3)} + f_{T(4)} + f_{T(4\text{-}2)} + f_{T(4\text{-}3)} + f_{T(5)} + f_{T(5\text{-}2)} + f_{T(5\text{-}3)} + f_{Q(1)} + f_{Q(1\text{-}2)} + f_{Q(1\text{-}3)} + f_{Q(2)} + f_{Q(2\text{-}2)} + f_{Q(2\text{-}3)} + f_{Se(1)}]\ .$$

Eq. S17

Table S22. Shieldings (in ppm) of carbon sites in each spin configuration for model **Ra1** at 298.2 K and the scaled ΔE between the spin and the ground spin configuration (s = 1.233, CAM-B3LYP/II//GFN2-xTB).

Site	σ_{Se}	σ_{S} [a]	$\sigma_{T(1)}$	$\sigma_{T(1-2)}$	$\sigma_{T(1-3)}$	$\sigma_{T(2)}$	$\sigma_{T(2-2)}$	$\sigma_{T(2-3)}$
C1	–4416.6	44.9	–698.1	–703.7	–701.7	–697.4	–703.5	–702.8
C3	–486.7	41.1	–44.3	–38.5	–45.9	–45.1	–38.7	–44.2
C2	1722.9	5.2	288.8	296.2	294.1	293.5	290.2	290.6
ΔE / cm^{-1}	811.5	[b]	270.4	270.4	270.3	270.5	270.4	270.3
	$\sigma_{T(3)}$	$\sigma_{T(3-2)}$	$\sigma_{T(3-3)}$	$\sigma_{T(4)}$	$\sigma_{T(4-2)}$	$\sigma_{T(4-3)}$	$\sigma_{T(5)}$	$\sigma_{T(5-2)}$
C1	–700.0	–699.4	–703.0	–695.9	–696.0	–702.5	–688.5	–697.2
C3	–44.6	–44.4	–46.0	–44.3	–44.7	–43.0	–43.6	–44.5
C2	287.8	284.4	295.0	294.9	298.9	290.3	290.0	292.0
ΔE / cm^{-1}	270.5	270.5	270.3	271.7	271.7	271.5	811.0	811.0
	$\sigma_{T(5-3)}$	$\sigma_{Q(1)}$	$\sigma_{Q(1-2)}$	$\sigma_{Q(1-3)}$	$\sigma_{Q(2)}$	$\sigma_{Q(2-2)}$	$\sigma_{Q(2-3)}$	
C1	–696.8	–2183.4	–2190.4	–2189.8	–2183.3	–2189.7	–2190.8	
C3	–45.3	–219.6	–219.4	–220.2	–219.3	–220.5	–219.7	
C2	290.9	864.8	859.7	867.1	862.5	870.8	862.2	
ΔE / cm^{-1}	811.0	541.1	540.9	541.0	541.1	540.9	541.0	

[a] Includes all singlet configurations. [b] Calculated explicitly for all singlet configurations; those with [↑↓][↑↓][↑↓] configuration (*e.g.*, Singlet(1) in **Fig. S13**) have ΔE of 0 - 1.2 cm^{-1}, those with [↑↑][↓↓][↑↓] configuration (*e.g.*, Singlet(5)) have ΔE of 540.2 - 541.5 cm^{-1}.

When more singlet configurations with higher energy are paired up, *i.e.*, through linear combination of Singlet(5) and Singlet(6), and their three-fold images, the following expression is obtained:

$$\sigma_{total} = [\tfrac{1}{2}(f_{S(1)} + f_{S(2)})\ \tfrac{1}{2}(\sigma_{S(1)} + \sigma_{S(2)}) + \tfrac{1}{2}(f_{S(3)} + f_{S(4)})\ \tfrac{1}{2}(\sigma_{S(3)} + \sigma_{S(4)}) + \tfrac{1}{2}(f_{S(3-2)} + f_{S(4-2)})\ \tfrac{1}{2}(\sigma_{S(3-2)} + \sigma_{S(4-2)}) + \tfrac{1}{2}(f_{S(3-3)} + f_{S(4-3)})\ \tfrac{1}{2}(\sigma_{S(3-3)} + \sigma_{S(4-3)}) + \tfrac{1}{2}(f_{S(5)} + f_{S(6)})\ \tfrac{1}{2}(\sigma_{S(5)} + \sigma_{S(6)}) + \tfrac{1}{2}(f_{S(5-2)} + f_{S(6-2)})\ \tfrac{1}{2}(\sigma_{S(5-2)} + \sigma_{S(6-2)}) + \tfrac{1}{2}(f_{S(5-3)} + f_{S(6-3)})\ \tfrac{1}{2}(\sigma_{S(5-3)} + \sigma_{S(6-3)}) + f_{T(1)}\sigma_{T(1)} + f_{T(1-2)}\sigma_{T(1-2)} + f_{T(1-3)}\sigma_{T(1-3)} + f_{T(2)}\sigma_{T(2)} + f_{T(2-2)}\sigma_{T(2-2)} + f_{T(2-3)}\sigma_{T(2-3)} + f_{T(3)}\sigma_{T(3)} + f_{T(3-2)}\sigma_{T(3-2)} + f_{T(3-3)}\sigma_{T(3-3)} + f_{T(4)}\sigma_{T(4)} + f_{T(4-2)}\sigma_{T(4-2)} + f_{T(4-3)}\sigma_{T(4-3)} + f_{T(5)}\sigma_{T(5)} + f_{T(5-2)}\sigma_{T(5-2)} + f_{T(5-3)}\sigma_{T(5-3)} + f_{Q(1)}\sigma_{Q(1)} + f_{Q(1-2)}\sigma_{Q(1-2)} + f_{Q(1-3)}\sigma_{Q(1-3)} + f_{Q(2)}\sigma_{Q(2)} + f_{Q(2-2)}\sigma_{Q(2-2)} + f_{Q(2-3)}\sigma_{Q(2-3)} + f_{Se(1)}\sigma_{Se(1)}]\ /\ [\tfrac{1}{2}(f_{S(1)} + f_{S(2)}) + \tfrac{1}{2}(f_{S(3)} + f_{S(4)}) + \tfrac{1}{2}(f_{S(3-2)} + f_{S(4-2)}) + \tfrac{1}{2}(f_{S(3-3)} + f_{S(4-3)}) + \tfrac{1}{2}(f_{S(5)} + f_{S(6)}) + \tfrac{1}{2}(f_{S(5-2)} + f_{S(6-2)}) + \tfrac{1}{2}(f_{S(5-3)} + f_{S(6-3)}) + f_{T(1)} + f_{T(1-2)} + f_{T(1-3)} + f_{T(2)} + f_{T(2-2)} + f_{T(2-3)} + f_{T(3)} + f_{T(3-2)} + f_{T(3-3)} + f_{T(4)} + f_{T(4-2)} + f_{T(4-3)} + f_{T(5)} + f_{T(5-2)} + f_{T(5-3)} + f_{Q(1)} + f_{Q(1-2)} + f_{Q(1-3)} + f_{Q(2)} + f_{Q(2-2)} + f_{Q(2-3)} + f_{Se(1)}]\ .$$

Eq. S18

Further linear combination of Triplet(1) and Triplet(2), Triplet(3) and Triplet(4), as well as Quintet(1) and Quintet(2) and their three-fold images affords

$$\sigma_{total} = [½(f_{S(1)} + f_{S(2)})\ ½(\sigma_{S(1)} + \sigma_{S(2)}) + ½(f_{S(3)} + f_{S(4)})\ ½(\sigma_{S(3)} + \sigma_{S(4)}) + ½(f_{S(3\text{-}2)} + f_{S(4\text{-}2)})\ ½(\sigma_{S(3\text{-}2)} + \sigma_{S(4\text{-}2)}) + ½(f_{S(3\text{-}3)} + f_{S(4\text{-}3)})\ ½(\sigma_{S(3\text{-}3)} + \sigma_{S(4\text{-}3)}) + ½(f_{S(5)} + f_{S(6)})\ ½(\sigma_{S(5)} + \sigma_{S(6)}) + ½(f_{S(5\text{-}2)} + f_{S(6\text{-}2)})\ ½(\sigma_{S(5\text{-}2)} + \sigma_{S(6\text{-}2)}) + ½(f_{S(5\text{-}3)} + f_{S(6\text{-}3)})\ ½(\sigma_{S(5\text{-}3)} + \sigma_{S(6\text{-}3)}) + ½(f_{T(1)} + f_{T(2)})\ ½(\sigma_{T(1)} + \sigma_{T(2)}) + ½(f_{T(1\text{-}2)} + f_{T(2\text{-}2)})\ ½(\sigma_{T(1\text{-}2)} + \sigma_{T(2\text{-}2)}) + ½(f_{T(1\text{-}3)} + f_{T(2\text{-}3)})\ ½(\sigma_{T(1\text{-}3)} + \sigma_{T(2\text{-}3)}) + ½(f_{T(3)} + f_{T(4)})\ ½(\sigma_{T(3)} + \sigma_{T(4)}) + ½(f_{T(3\text{-}2)} + f_{T(4\text{-}2)})\ ½(\sigma_{T(3\text{-}2)} + \sigma_{T(4\text{-}2)}) + ½(f_{T(3\text{-}3)} + f_{T(4\text{-}3)})\ ½(\sigma_{T(3\text{-}3)} + \sigma_{T(4\text{-}3)}) + f_{T(5)}\ \sigma_{T(5)} + f_{T(5\text{-}2)}\ \sigma_{T(5\text{-}2)} + f_{T(5\text{-}3)}\ \sigma_{T(5\text{-}3)} + ½(f_{Q(1)} + f_{Q(2)})\ ½(\sigma_{Q(1)} + \sigma_{Q(2)}) + ½(f_{Q(1\text{-}2)} + f_{Q(2\text{-}2)})\ ½(\sigma_{Q(1\text{-}2)} + \sigma_{Q(2\text{-}2)}) + ½(f_{Q(1\text{-}3)} + f_{Q(2\text{-}3)})\ ½(\sigma_{Q(1\text{-}3)} + \sigma_{Q(2\text{-}3)}) + f_{Se(1)}\ \sigma_{Se(1)}] \ / \ [½(f_{S(1)} + f_{S(2)}) + ½(f_{S(3)} + f_{S(4)}) + ½(f_{S(3\text{-}2)} + f_{S(4\text{-}2)}) + ½(f_{S(3\text{-}3)} + f_{S(4\text{-}3)}) + ½(f_{S(5)} + f_{S(6)}) + ½(f_{S(5\text{-}2)} + f_{S(6\text{-}2)}) + ½(f_{S(5\text{-}3)} + f_{S(6\text{-}3)}) + ½(f_{T(1)} + f_{T(2)}) + ½(f_{T(1\text{-}2)} + f_{T(2\text{-}2)}) + ½(f_{T(1\text{-}3)} + f_{T(2\text{-}3)}) + ½(f_{T(3)} + f_{T(4)}) + ½(f_{T(3\text{-}2)} + f_{T(4\text{-}2)}) + ½(f_{T(3\text{-}3)} + f_{T(4\text{-}3)}) + f_{T(5)} + f_{T(5\text{-}2)} + f_{T(5\text{-}3)} + ½(f_{Q(1)} + f_{Q(2)}) + ½(f_{Q(1\text{-}2)} + f_{Q(2\text{-}2)}) + ½(f_{Q(1\text{-}3)} + f_{Q(2\text{-}3)}) + f_{Se(1)}] \ . \qquad \textbf{Eq. S19}$$

Finally, it could be assumed that the (near-)degenerate configurations with the same multiplicity are part of the same state, *i.e.*, Singlet(1) - Singlet(4-3) labelled Singlet(a), Singlet(5) - Singlet(6-3) labelled Singlet(b), Triplet(1) - Triplet(4-3) labelled Triplet(a), Triplet(5) - Triplet(5-3) labelled Triplet(b), and Quintet(1) - Quintet(2-3) labelled Quintet. The corresponding shielding constants are evaluated as

$$\sigma_{S(a)} = ⅛\ (\sigma_{S(1)} + \sigma_{S(2)} + \sigma_{S(3)} + \sigma_{S(4)} + \sigma_{S(3\text{-}2)} + \sigma_{S(4\text{-}2)} + \sigma_{S(3\text{-}3)} + \sigma_{S(4\text{-}3)})\ , \qquad \textbf{Eq. S20}$$

$$\sigma_{S(b)} = ⅙\ (\sigma_{S(5)} + \sigma_{S(6)} + \sigma_{S(5\text{-}2)} + \sigma_{S(6\text{-}2)} + \sigma_{S(5\text{-}3)} + \sigma_{S(6\text{-}3)})\ , \qquad \textbf{Eq. S21}$$

$$\sigma_{T(a)} = 1/12\ (\sigma_{T(1)} + \sigma_{T(2)} + \sigma_{T(1\text{-}2)} + \sigma_{T(2\text{-}2)} + \sigma_{T(1\text{-}3)} + \sigma_{T(2\text{-}3)} + \sigma_{T(3)} + \sigma_{T(4)} + \sigma_{T(3\text{-}2)} + \sigma_{T(4\text{-}2)} + \sigma_{T(3\text{-}3)} + \sigma_{T(4\text{-}3)})\ , \qquad \textbf{Eq. S22}$$

$$\sigma_{T(b)} = ⅓\ (\sigma_{T(5)} + \sigma_{T(5\text{-}2)} + \sigma_{T(5\text{-}3)})\ , \qquad \textbf{Eq. S23}$$

$$\sigma_{Q} = ⅙\ (\sigma_{Q(1)} + \sigma_{Q(1\text{-}2)} + \sigma_{Q(1\text{-}3)} + \sigma_{Q(2)} + \sigma_{Q(2\text{-}2)} + \sigma_{Q(2\text{-}3)})\ , \qquad \textbf{Eq. S24}$$

and the following expression for the total shielding is

$$\sigma_{total} = [\tfrac{1}{8} (f_{S(1)} + f_{S(2)} + f_{S(3)} + f_{S(4)} + f_{S(3\text{-}2)} + f_{S(4\text{-}2)} + f_{S(3\text{-}3)} + f_{S(4\text{-}3)})\ \sigma_{S(a)} + \tfrac{1}{6} (f_{S(5)} + f_{S(6)} + f_{S(5\text{-}2)} + f_{S(6\text{-}2)} + f_{S(5\text{-}3)} + f_{S(6\text{-}3)})\ \sigma_{S(b)} + 1/12\ (f_{T(1)} + f_{T(2)} + f_{T(1\text{-}2)} + f_{T(2\text{-}2)} + f_{T(1\text{-}3)} + f_{T(2\text{-}3)} + f_{T(3)} + f_{T(4)} + f_{T(3\text{-}2)} + f_{T(4\text{-}2)} + f_{T(3\text{-}3)} + f_{T(4\text{-}3)})\ \sigma_{T(a)} + \tfrac{1}{3} (f_{T(5)} + f_{T(5\text{-}2)} + f_{T(5\text{-}3)})\ \sigma_{T(b)} + \tfrac{1}{6} (f_{Q(1)} + f_{Q(1\text{-}2)} + f_{Q(1\text{-}3)} + f_{Q(2)} + f_{Q(2\text{-}2)} + f_{Q(2\text{-}3)})\ \sigma_{Q} + f_{Se(1)}\ \sigma_{Se(1)}] \,/\, [\tfrac{1}{8} (f_{S(1)} + f_{S(2)} + f_{S(3)} + f_{S(4)} + f_{S(3\text{-}2)} + f_{S(4\text{-}2)} + f_{S(3\text{-}3)} + f_{S(4\text{-}3)}) + \tfrac{1}{6} (f_{S(5)} + f_{S(6)} + f_{S(5\text{-}2)} + f_{S(6\text{-}2)} + f_{S(5\text{-}3)} + f_{S(6\text{-}3)}) + 1/12\ (f_{T(1)} + f_{T(2)} + f_{T(1\text{-}2)} + f_{T(2\text{-}2)} + f_{T(1\text{-}3)} + f_{T(2\text{-}3)} + f_{T(3)} + f_{T(4)} + f_{T(3\text{-}2)} + f_{T(4\text{-}2)} + f_{T(3\text{-}3)} + f_{T(4\text{-}3)}) + \tfrac{1}{3} (f_{T(5)} + f_{T(5\text{-}2)} + f_{T(5\text{-}3)}) + \tfrac{1}{6} (f_{Q(1)} + f_{Q(1\text{-}2)} + f_{Q(1\text{-}3)} + f_{Q(2)} + f_{Q(2\text{-}2)} + f_{Q(2\text{-}3)}) + f_{Se(1)}]\ . \qquad \textbf{Eq. S25}$$

So far, no use of symmetry has been made. As the tri-dimer model has C_3 symmetry, the fourteen selected configurations in **Fig. S13** should represent all 36 configurations. The energy difference between configurations with the same pattern for the spin distribution, just rotated by 120° or 240°, is smaller than 1 cm^{-1}. For example, Triplet(1), Triplet(1-2) and Triplet(1-3) have almost identical energies at 270.4 cm^{-1} above the ground state Singlet(1), and reasonably close averaged shielding values of the C1, C2 and C3 sites (**Table S22**). With imposing symmetry, **Eq. S19** can be simplified to

$$\sigma_{total} = [f_{S(1)}\ \tfrac{1}{2}(\sigma_{S(1)} + \sigma_{S(2)}) + 3 \times f_{S(3)}\ \tfrac{1}{2}(\sigma_{S(3)} + \sigma_{S(4)}) + 3 \times \tfrac{1}{2}(f_{S(5)} + f_{S(6)})\ \tfrac{1}{2}(\sigma_{S(5)} + \sigma_{S(6)}) + 3 \times \tfrac{1}{2}(f_{T(1)} + f_{T(2)})\ \tfrac{1}{2}(\sigma_{T(1)} + \sigma_{T(2)}) + 3 \times \tfrac{1}{2}(f_{T(3)} + f_{T(4)})\ \tfrac{1}{2}(\sigma_{T(3)} + \sigma_{T(4)}) + 3 \times f_{T(5)}\ \sigma_{T(5)} + 3 \times \tfrac{1}{2}(f_{Q(1)} + f_{Q(2)})\ \tfrac{1}{2}(\sigma_{Q(1)} + \sigma_{Q(2)}) + f_{Se(1)}\ \sigma_{Se(1)}\ ] \,/\, [f_{S(1)} + 3 \times f_{S(3)} + 3 \times \tfrac{1}{2}(f_{S(5)} + f_{S(6)}) + 3 \times \tfrac{1}{2}(f_{T(1)} + f_{T(2)}) + 3 \times \tfrac{1}{2}(f_{T(3)} + f_{T(4)}) + 3 \times f_{T(5)} + 3 \times \tfrac{1}{2}(f_{Q(1)} + f_{Q(2)}) + f_{Se(1)}]\ , \qquad \textbf{Eq. S26}$$

to calculate the total shielding of a site for in a tri-dimer model (see **Fig. S13** for pictorial representation). Note that the simplified expression has the factor of 3 in front of all terms except for the terms of the ground and high-spin states, which accounts for the presence of configurations that are related by the three-fold symmetry.

Following the validation of combining the possible configurations into fewer states discussed above for di-dimer models, a similar test is now applied to model **Ra1** for calculating ^{13}C shifts of activated HKUST-1. Averaged as-calculated shieldings of C1, C2 and C3 for the model in each spin configuration and the scaled energy difference between the spin and ground spin state (ΔE) are listed in **Table S22** (scaled as discussed above). Note that here all singlet configurations are assumed to have identical

shielding values (σ_S), whereas all possible configurations with higher multiplicity (including those that are formally symmetry equivalent) are shown separately. For example, $\sigma_{T(1)}$, $\sigma_{T(1-2)}$ and $\sigma_{T(1-3)}$ correspond to three symmetry-equivalent configurations, one of which is shown in **Fig. S13** as Triplet(1), and all of these are included in **Table S22**. In this case, the shieldings of the formally symmetry-equivalent configurations are indeed very similar. These configurations, and those with the same number of ferromagnetically coupled dimers, are energetically near-degenerate, consistent with the results for the di-dimer model.

The computed C1, C2 and C3 shifts for model **Ra1** show large variations when using these equations stated above. None of these matches the experimental shifts for activated HKUST-1 using as-calculated ΔE (see **Fig. S14A-C**). However, scaling ΔE using **Eq. 3** and fitting s to minimise the MAD between calculation and experiment at all temperatures together leads to a good agreement with experiment for some of the calculated sets (see **Fig. S14D-F**). It is obvious that combining all degenerate configurations with the same multiplicity is not valid, as the shifts computed according to **Eq. S25** do not match up with experiment at all (note the completely different trends of the dark purple line and the experimental data points in **Fig. S14D-F**). The shifts computed from **Eqs. S16 – S19** show very similar trends and are hard to distinguish from each other in this figure.

The expressions can be compared by plotting the MADs between computed and experimental shifts for C1, C2 and C3 at different temperatures (**Fig. S15A**). **Fig. S15B** shows averaged MADs across the whole temperature range for different carbon sites. In line with **Fig. S14**, **Eq. S25** is not an appropriate expression for computing shifts for this model because it produces high MADs. At different temperatures, the MADs for the remaining expressions, **Eqs. S16** – **S19**, are similar and all under 15 ppm. The averaged MADs for **Eq. S19** are the lowest at 9.6 ppm, having the best agreement of computed shifts with experiment. The largest errors are associated with the shift of C2, which can deviate by over 15 ppm (similarly underestimated as for the computed shift of the analogous site in urea loaded copper benzoate[S5]). Overall, **Eq. S19** appears to be an appropriate expression of computing shifts for model **Ra1**.

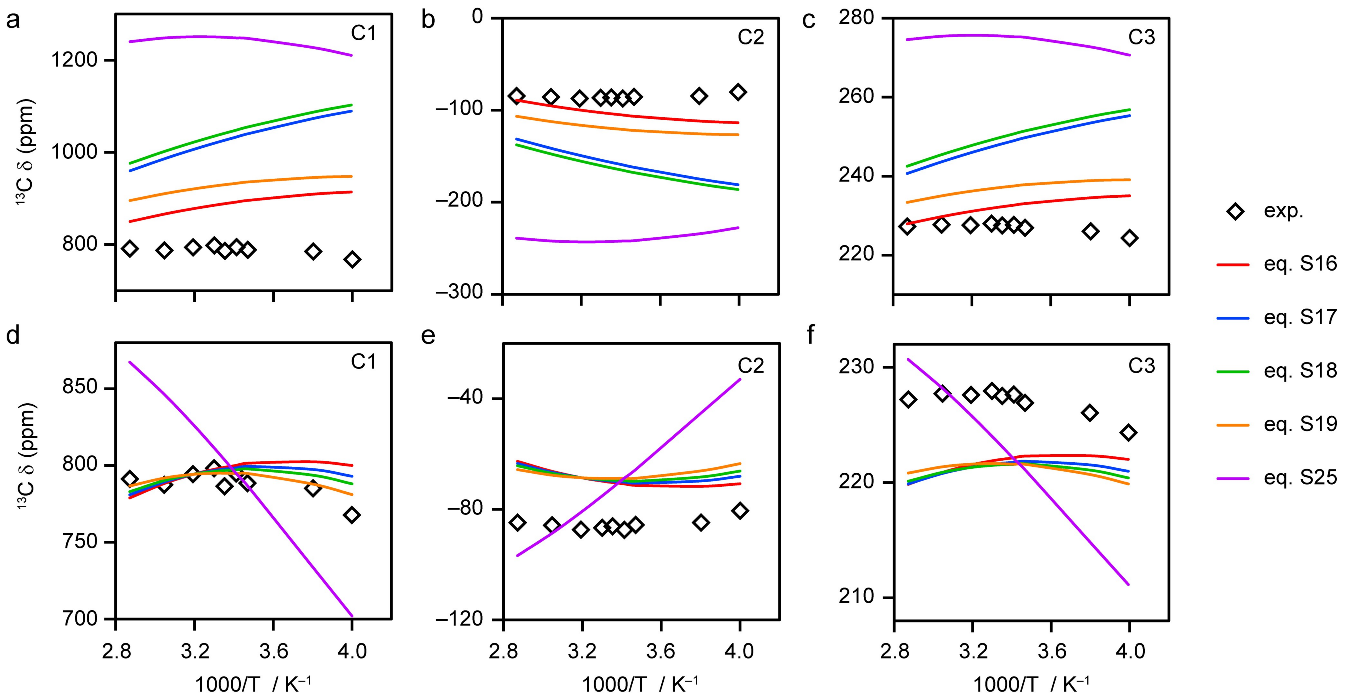

Fig. S14. Plots of temperature variation of the ^{13}C shifts for model **Ra1**, using **Eqs. S16, S17, S18, S19** or **S25** at the CAM-B3LYP/II//GFN2-xTB level. Shifts were calculated using (a-c) as-calculated ΔE_{ST} (~219 cm^{-1}) and (d-f) scaled (using **Eq. 3**) ΔE_{ST} with s = 1.171, 1.520, 1.538, 1.223 and 1.450 for **Eqs. S16, S17, S18, S19** and **S25**, respectively. See **Fig. 1D** for the site numbering scheme. Experimental points for activated HKUST-1 are also shown.

Representative spin configurations with corresponding computed shieldings of carbon sites and scaled energy differences are shown in **Table S23** (a selection from the full set in **Table S22**). Using either the full (**Eq. S19**) or simplified expression (**Eq. S26**) for calculating shifts produces results of the same quality for activated HKUST-1, using similar scaling factors of 1.233 and 1.230, respectively (**Table S24**). The mean MADs for model **Ra1** are all below 12 ppm. It seems that the agreement gets poorer when the model is increased from the di-dimer to the tri-dimer, with around 5 ppm higher averaged MAD. One could speculate that the reason for this poorer agreement is the increased number of spin states in the tri-dimer model, which could introduce more potential for error. However, the computed temperature-dependent behaviour of the shifts for the tri-dimer model show slightly better agreement with the experiment (**Fig. S16**). Therefore, it is hard to decide which model would be best for activated HKUST-1.

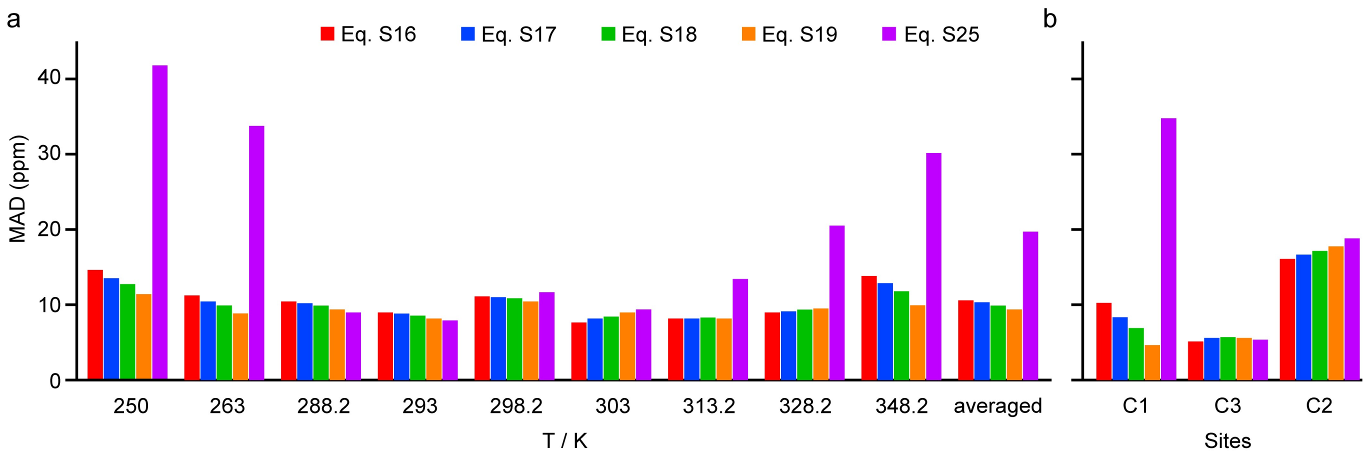

Fig. S15. MADs (a) at each temperature and (b) for sites 1, 2 and 3 in activated HKUST-1 employing **Eqs. S16, S17, S18, S19** or **S25** to calculate ^{13}C shifts for model **Ra1** with s = 1.171, 1.520, 1.538, 1.233 and 1.450, respectively (CAM-B3LYP/II//GFN2-xTB).

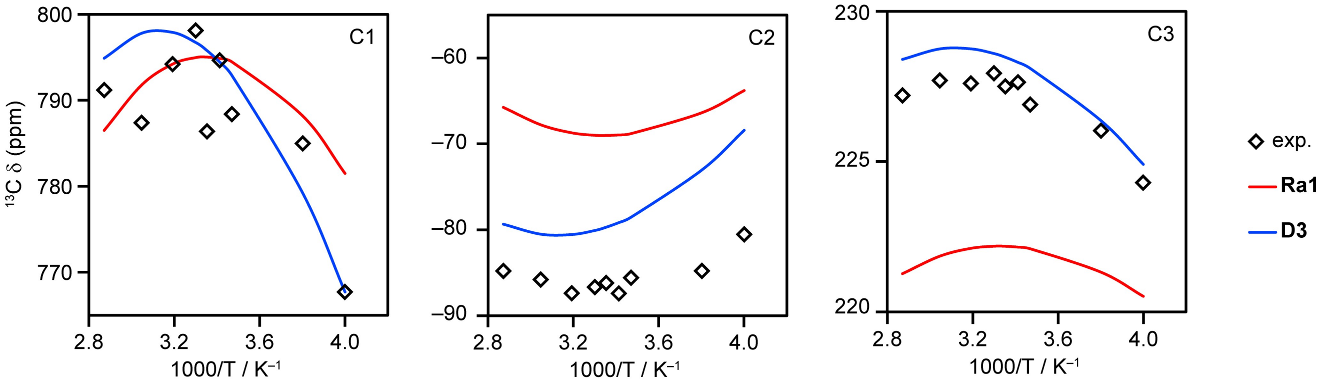

Fig. S16. Temperature dependence of experimental and calculated ^{13}C shifts of activated HKUST-1, using **Eq. S26** in the calculation for model **Ra1** with s = 1.230 for ΔE_{ST} of ~219 cm^{-1} (CAM-B3LYP/II//GFN2-xTB). The calculated ^{13}C shifts for model **D3** have been added from **Table S10**. (a), (b) and (c) are shifts for C1, C3 and C2 in HKUST-1, respectively

Table S23. Shieldings (in ppm) of carbon sites in selected spin configurations at 298.2 K and the energy difference between the spin configuration and the ground spin configuration (s = 1.230) for model **Ra1** (CAM-B3LYP/II//GFN2-xTB).

Site[a]	σ_{Se}	$\sigma_{S(1)\text{-}(6)}$	$\sigma_{T(1)}$	$\sigma_{T(2)}$	$\sigma_{T(3)}$	$\sigma_{T(4)}$	$\sigma_{T(5)}$	$\sigma_{Q(1)}$	$\sigma_{Q(2)}$
C1	–4416.6	44.9	–698.1	–697.4	–700.0	–695.9	–688.5	–2183.4	–2183.3
C3	–486.7	41.1	–44.3	–45.1	–44.6	–44.3	–43.6	–219.6	–219.3
C2	1722.9	5.2	288.8	293.5	287.8	294.9	290.0	864.8	862.5
ΔE / cm^{-1}	809.2	[b]	269.6	269.7	269.7	270.9	808.7	539.6	539.6

[a] Symmetry-equivalent sites. [b] Singlet(1) - Singlet(6): 0, –, 1.2, –, 538.8, 539.9, – (where – denotes omitted value).

**Table S24.** Calculated ^{13}C shifts (in ppm) at different temperatures for model **Ra1** with s = 1.233 and 1.230, respectively, for **Eqs. S19** and **S26**, for ΔE_{ST} ≈186 cm^{-1} (CAM-B3LYP/II//GFN2-xTB). Experimental shifts of activated HKUST-1 are also given.									
T / K	250	263	288.2	293	298.2	303	313.2	328.2	348.2
1000/T / K^{-1}	0.25	0.26	0.29	0.29	0.30	0.30	0.31	0.33	0.35
Exp.									
C1	767.7	785.0	788.4	794.7	786.4	798.1	794.2	787.4	791.2
C3	224.3	226.0	226.9	227.6	227.5	227.9	227.6	227.7	227.2
C2	–80.5	–84.8	–85.6	–87.4	–86.2	–86.7	–87.4	–85.8	–84.8
**Eq. S19**									
C1	781.1	787.8	794.8	794.7	794.9	794.9	794.2	791.8	786.6
C3	219.8	220.7	221.5	221.6	221.6	221.6	221.6	221.3	220.8
C2	–63.5	–66.1	–68.8	–68.7	–68.8	–68.8	–68.5	–67.6	–65.6
MAD	11.6	9.0	9.5	8.3	10.6	9.1	8.3	9.7	10.1
**Eq. S26**									
C1	781.5	788.2	794.5	794.8	795.0	795.0	794.2	791.8	786.5
C3	220.5	221.3	222.1	222.2	222.2	222.2	222.1	221.9	221.3
C2	–63.8	–66.4	–68.8	–68.9	–69.0	–69.0	–68.7	–67.8	–65.7
MAD	11.4	8.8	9.2	8.1	10.4	8.9	8.1	9.4	9.9

**Table S25.** ^{13}C shieldings (in ppm) in each selected spin configuration at 298.4 K and the scaled energy differences between the spin configuration and the ground spin configuration (s = 1.141) for model **Ra2** (CAM-B3LYP/II//GFN2-xTB).									
Site[a]	σ_{Se}	$\sigma_{S(1)\text{-}(6)}$	$\sigma_{T(1)}$	$\sigma_{T(2)}$	$\sigma_{T(3)}$	$\sigma_{T(4)}$	$\sigma_{T(5)}$	$\sigma_{Q(1)}$	$\sigma_{Q(2)}$
C1	–3884.9	44.5	–607.2	–609.5	–746.7	–604.5	–470.1	–1814.6	–2022.0
C3	–394.9	42.8	–30.1	–31.3	–72.5	–29.4	11.4	–145.5	–208.1
C2	1274.2	6.5	219.7	214.3	294.5	217.0	139.8	581.5	697.4
ΔE / cm^{-1}	638.1	[b]	212.6	212.5	212.5	213.3	638.0	424.8	424.9
[a] Symmetry-equivalent sites. [b] Singlet(1) - Singlet(6): 0.00, –, 0.9, –, 424.8, 425.8 (where – denotes omitted value)									

**Table S26.** ^{13}C shieldings (in ppm) in each selected spin configuration at 298.2 K and the energy difference between the spin and the ground spin configuration (s = 1.230) for model **Ra1** (CAM-B3LYP/II//GFN2-xTB).									
Site[a]	σ_{Se}	$\sigma_{S(1)\text{-}(6)}$	$\sigma_{T(1)}$	$\sigma_{T(2)}$	$\sigma_{T(3)}$	$\sigma_{T(4)}$	$\sigma_{T(5)}$	$\sigma_{Q(1)}$	$\sigma_{Q(2)}$
C1	–4416.6	44.9	–698.1	–697.4	–700.0	–695.9	–688.5	–2183.4	–2183.3
C3	–486.7	41.1	–44.3	–45.1	–44.6	–44.3	–43.6	–219.6	–219.3
C2	1722.9	5.2	288.8	293.5	287.8	294.9	290.0	864.8	862.5
ΔE / cm^{-1}	809.2	[b]	269.6	269.7	269.7	270.9	808.7	539.6	539.6
[a] Symmetry-equivalent sites. [b]. Singlet(1) - Singlet(6): 0.0, –, 1.2, –, 538.8, 539.9 (where – denotes omitted value)									

Table S27. Calculated ^{13}C shifts (in ppm) at different temperatures using model **Ra2** with s = 1.141 for ΔE_{ST} of ~186 cm^{-1} (CAM-B3LYP/II//GFN2-xTB), using **Eq. S26**. Experimental shifts of hydrated HKUST-1 are given for comparison.[S3]

Exp.[a]													
T / K	250†	258	263†	263	268	273	278	283	293†	298	303†	313	323
1000/T / K^{-1}	4.0	3.9	3.8	3.8	3.7	3.7	3.6	3.5	3.4	3.4	3.3	3.2	3.1
C1	870.8	868.2	863.4	865.4	865.0	/	858.6	/	851.8	848.2	845.8	843.1	838.1
C3	230.3	229.1	229.6	228.7	228.5	228.5	228.2	228.1	227.8	227.4	227.2	226.7	226.4
C2	–58.7	–54.8	–57.3	–54.5	–54.2	–52.9	–52.7	–50.3	–50.5	–50.5	–49.2	–50.8	–43.2
Calc.													
T / K	250	258	263	268	273	278	283	288	293	298	303	313	323
1000/T / K^{-1}	4.0	3.9	3.8	3.7	3.7	3.6	3.5	3.5	3.4	3.4	3.3	3.2	3.1
C1	870.8	868.8	867.3	865.4	863.4	861.1	858.7	856.1	853.4	850.5	847.5	841.2	834.5
C3	226.3	226.1	225.9	225.7	225.4	225.2	224.9	224.6	224.3	224.0	223.6	222.9	222.2
C2	–53.8	–53.1	–52.6	–52.0	–51.4	–50.7	–49.9	–49.0	–48.2	–47.2	–46.3	–44.2	–42.1
MAD[b]	{3.0}	1.8	2.2 {4.1}	1.8	2.3	2.5	1.8	/	{2.5}	3.0	{2.7}	4.1	3.0

[a] Data taken from a mix of two series of experiments in both shim temperature controlled and no shim temperature control (see main text), † = shim temperature not controlled. [b] Values in curly brackets are calculated for another experimental measurement with no shim temperature control.

Ring tri-dimer models

The ring tri-dimer models, for example **Ri1** shown in **Fig. S17** (see **Table 1** for details of other models) are proposed to model this closed circular structure. This has the same total number of thirty-six possible spin configurations as the radial tri-dimer models, which can be simplified into fourteen configurations as shown in **Fig. S17**; however, the topology is different. While the radial model has a cyclic arrangement of six spin centres in one plane (idealised symmetry D_{3h}) with all centres in the same horizontal mirror plane and none on a vertical plane, the cyclic model is puckered (idealised C_{3v} symmetry) with one pair of spin centres in each of the vertical planes. Again, the calculated coupling energies show the interdimer interactions are very weak, e.g., the energy difference between the Septet(1) and Triplet(5) spin configurations in the "ring" model is <1 cm^{-1}.

The calculation of ^{13}C shifts for model **Ri1** employs the simplified expression in **Eq. S26** to combine shieldings of eight spin states from fourteen spin configurations in total (**Table S28**). This model is used to compute ^{13}C shifts for activated HKUST-1 by minimising the MAD at all temperatures. The fitted results for the ring model do not agree as well with experiment as those for model **Ra1** (*cf.* **Figs. S16** and **S18**). This may be due to the structural differences between both models, which have a different number of dimers on a linker molecule (three in **Ra1** vs. two in **Ri1**). With the same number of dimers on the linker, model **D1** is preferred over **Ri1** for HKUST-1 (**Fig. S18**). Even through the ring system is part of the MOF, capturing this more accurately in the model offers no improvement.

Table S28. Shieldings (in ppm) in each selected spin configuration at 298.2 K and the scaled energy differences between the spin configuration and the ground spin configuration (s = 1.198) for model **Ri1** (CAM-B3LYP/II//GFN2-xTB).

Site	σ_{Se}	$\sigma_{S(1)\text{-}(6)}$	$\sigma_{T(1)}$	$\sigma_{T(2)}$	$\sigma_{T(3)}$	$\sigma_{T(4)}$	$\sigma_{T(5)}$	$\sigma_{Q(1)}$	$\sigma_{Q(2)}$
C1[a]	–4146.3	44.4	–654.9	–654.8	–618.5	–690.7	–652.2	–2026.4	–2079.9
C4[a]	–213.2	43.7	2.1	2.5	–92.0	95.6	1.5	–152.5	–12.5
C5[b]	–270.5	41.3	–12.1	–12.1	–106.7	83.9	–10.6	–188.5	–45.0
C3[b]	–497.9	47.8	–41.9	–41.7	159.0	–243.2	–42.8	–71.2	–373.8
C2[a]	1851.2	3.8	311.6	310.9	313.0	309.6	309.0	928.9	926.7
C6[b]	133.5	164.9	160.2	160.3	147.1	172.9	160.1	140.2	159.5
ΔE / cm^{-1}	769.5	[c]	256.1	256.2	256.1	258.6	768.8	512.7	515.0

[a] Average of six equivalent sites in the model. [b] Average of three equivalent sites in the model. [c] Singlet(1) - Singlet(6): 0.0, –, 1.2, –, 512.5, 512.3 (where – denotes omitted value).

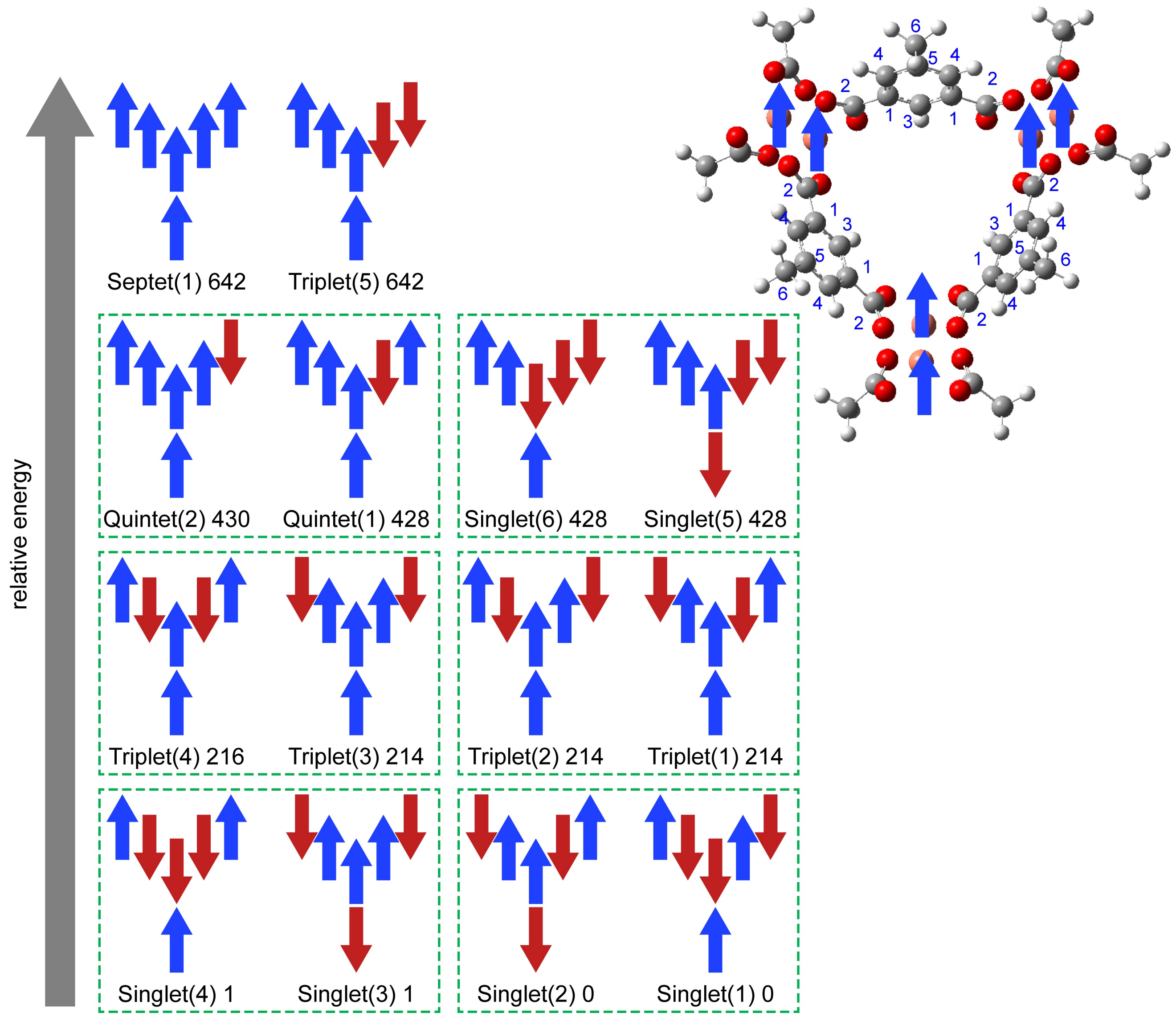

Fig. S17. Spin configurations of model **Ri1** (see **Fig. 2** and **Table 1**). All nomenclature and colouring schemes are as in **Fig. S1**.

Table S29. Shieldings (in ppm) in each selected spin configuration at 298 K and the scaled energy differences between the spin configuration Singlet(1) (s = 1.204) for model **Ri4** (CAM-B3LYP/II//GFN2-xTB).

Site	σ_{Se}	$\sigma_{S(1)\text{-}(6)}$	$\sigma_{T(1)}$	$\sigma_{T(2)}$	$\sigma_{T(3)}$	$\sigma_{T(4)}$	$\sigma_{T(5)}$	$\sigma_{Q(1)}$	$\sigma_{Q(2)}$
C1[a]	–3692.1	43.5	–607.6	–578.2	–548.5	–573.7	–577.8	–1799.2	–1843.6
C4[a]	–156.2	45.3	97.0	9.9	–76.6	10.3	10.5	–122.2	8.3
C5[b]	–231.9	41.6	80.4	–2.6	–84.7	–1.4	–2.1	–155.3	–31.1
C3[b]	–403.9	48.4	–200.0	–28.2	143.0	–28.4	–28.5	–50.4	–307.5
C2[a]	1388.5	5.0	264.1	234.6	204.8	232.4	234.6	673.3	718.2
C6[b]	137.6	164.8	171.4	159.7	148.2	160.0	159.9	142.0	159.4
ΔE / cm^{-1}	662.5	[c]	221.0	221.0	221.5	222.3	661.4	442.6	441.7

[a] Average of six equivalent sites in the model. [b] Average of three equivalent sites in the model. [c] Singlet(1) - Singlet(6): 0.0, –, 1.2, –, 441.1, 441.3 (where – denotes omitted value).

Table S30. Experimental[S2] and calculated ^{13}C shifts (in ppm) at different temperatures for hydrated STAM-17-Me model **Ri4**, using **Eq. S26** with s = 1.204 on ΔE_{ST} of ~184 cm^{-1} (CAM-B3LYP/II//GFN2-xTB).

T / K	278	298	318	338
1000/T / K^{-1}	3.60	3.36	3.14	2.96
Exp.[S2] STAM-17-Me				
C1	/	770.0	/	/
C3	222.5	221.3	219.8	218.0
C4, C5	179.7	179.3	178.7	177.9
C6	25.0	24.8	24.7	24.5
C2	–61.4	–60.3	–55.8	–49.6
Calc.				
C1[a]	803.6	795.6	785.5	774.0
C3[b]	220.0	219.0	217.7	216.3
C5[b]	193.1	192.5	191.8	191.0
C4[a]	178.8	178.3	177.8	177.1
C6[b]	27.8	27.7	27.6	27.5
C2[a]	–61.4	–58.5	–54.8	–50.5
MAD	3.9	4.2 (7.8) [c]	4.0	3.9

[a] Average of six equivalent sites in the model. [b] Average of three equivalent sites in the model. [c] The value in parentheses includes C1 at 298 K.

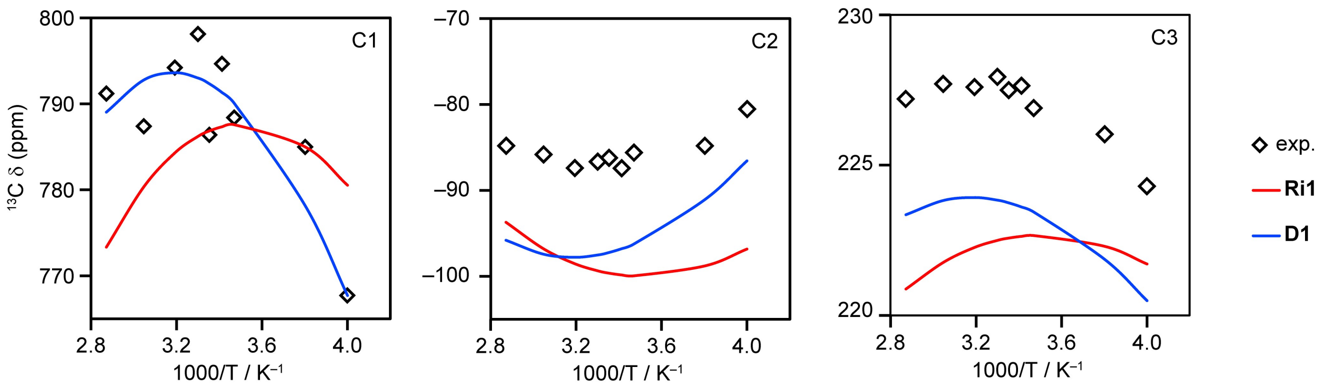

Fig. S18. Temperature dependence of experimental and calculated ^{13}C shifts for activated HKUST-1, using **Eq. S26** in the calculation for model **Ri1** with s = 1.198 on ΔE_{ST} of ~214 cm^{-1} (CAM-B3LYP/II//GFN2-xTB). Calculated shifts for model **D1** (see **Table S6**) are plotted for comparison.

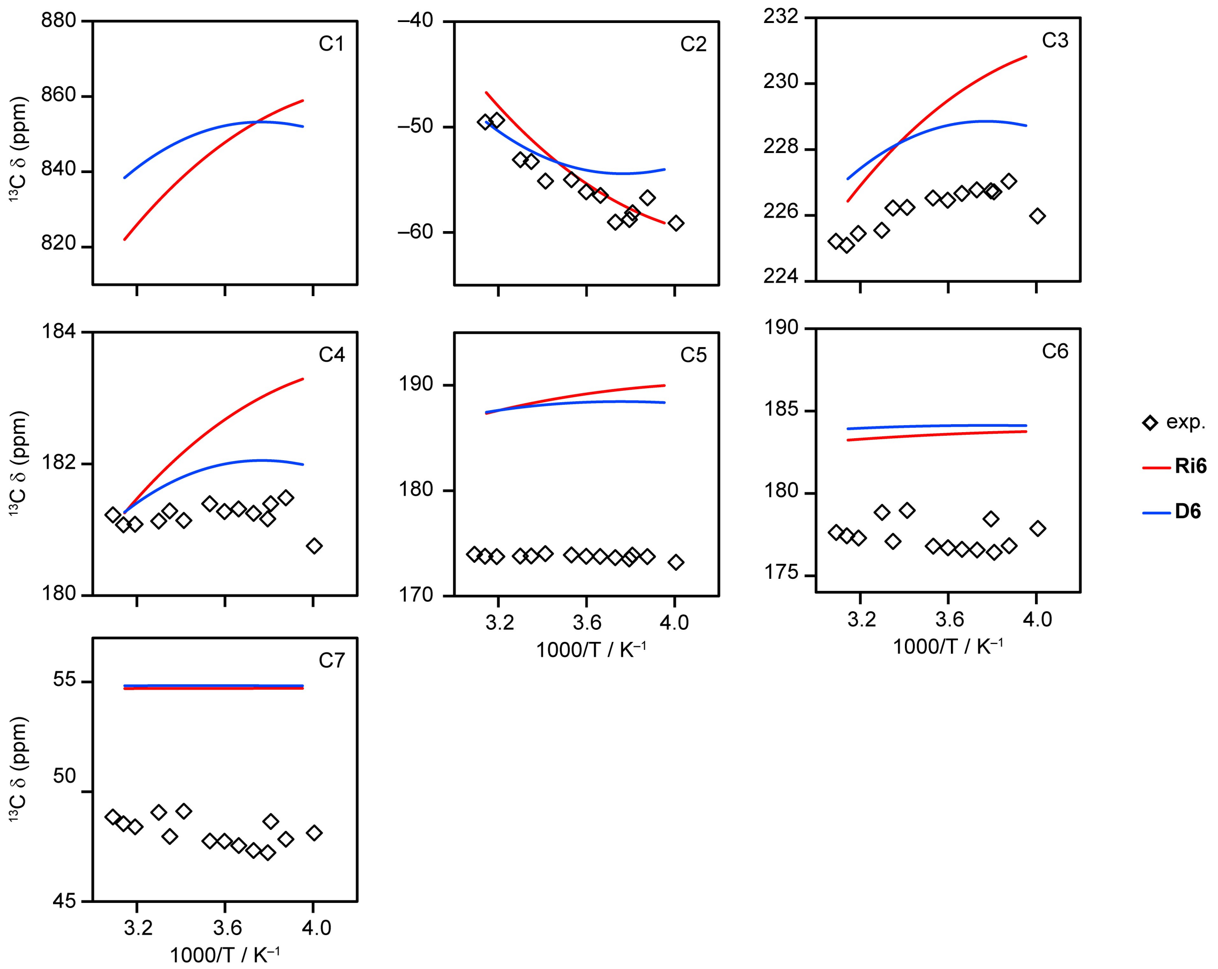

Fig. S19. Temperature dependence of experimental[S3] and calculated ^{13}C shifts for sites C1-C7 of hydrated STAM-1 (see labelling scheme in **Fig. 1D**.), using **Eq. S26** in the calculation for model **Ri6** with s = 1.135 on ΔE_{ST} of ~182 cm^{-1} (CAM-B3LYP/II//GFN2-xTB). C1 was not observed in the experimental dataset used). Calculated ^{13}C resonances for model **D6** are added for comparison.

Table S31. Shieldings (in ppm) in each selected spin configuration at 298 K and the scaled energy differences between the spin configuration and Singlet(1) (s = 1.183) for model **Ri5** (CAM-B3LYP/II//GFN2-xTB).

Site	σ_{Se}	$\sigma_{S(1)-(6)}$	$\sigma_{T(1)}$	$\sigma_{T(2)}$	$\sigma_{T(3)}$	$\sigma_{T(4)}$	$\sigma_{T(5)}$	$\sigma_{Q(1)}$	$\sigma_{Q(2)}$
C1[a]	–3749.3	42.3	–608.3	–590.0	–568.6	–586.7	–589.6	–1837.0	–1866.5
C4[a]	–152.9	61.4	119.9	25.4	–71.7	25.3	25.7	–119.2	24.4
C5[b]	–337.7	18.7	76.0	–40.3	–153.2	–39.2	–40.0	–243.4	–71.3
C3[b]	–391.7	54.0	–190.9	–20.2	147.4	–21.1	–20.3	–43.3	–297.1
C2[a]	1353.3	5.2	261.6	229.2	196.0	227.0	229.7	653.2	703.2
C6[b]	125.5	130.9	132.2	130.1	127.7	130.2	130.1	126.6	129.8
ΔE / cm^{-1}	647.5	[c]	216.0	216.0	215.8	217.4	645.3	432.8	431.0

[a] Average of six equivalent sites in the model. [b] Average of three equivalent sites in the model. [c] Singlet(1) - Singlet(6): 0.0, –, 1.0, –, 430.3, 430.1 (where – denotes omitted value).

**Table S32.** Experimental[S2] and calculated ^{13}C chemical shifts (in ppm) of hydrated STAM-17-OMe at different temperatures using **Eq. S26** for model **Ri5** with s = 1.183 on ΔE_{ST} of ~183 cm^{-1} (CAM-B3LYP/II//GFN2-xTB).				
T / K	278	298	318	338
1000/T / K^{-1}	3.60	3.36	3.14	2.96
Exp.[S2]				
C1	/	/	/	/
C5	219.2	218.7	217.9	216.8
C3	217.7	216.4	214.5	212.9
C4	166.0	165.7	165.0	164.1
C6	49.9	50.0	50.3	50.5
C2	–60.3	–57.4	–52.5	–48.3
Calc.				
C1[a]	828.5	819.1	807.7	795.1
C5[b]	232.1	231.2	230.2	229.0
C3[b]	214.4	213.3	212.0	210.4
C4[a]	165.4	164.8	164.2	163.4
C6[b]	57.6	57.5	57.5	57.5
C2[a]	–60.3	–56.9	–52.9	–48.4
MAD	4.9	4.9	4.7	4.5

[a] Average of six equivalent sites in the model. [b] Average of three equivalent sites in the model.

Table S33. Shieldings (in ppm) in each selected spin configuration at 298 K and the scaled energy differences between the spin configuration and Singlet(1) (s = 1.135) for model **Ri6** (CAM-B3LYP/II//GFN2-xTB).

Site[a]	σ_{Se}	$\sigma_{S(1)-(6)}$	$\sigma_{T(1)}$	$\sigma_{T(2)}$	$\sigma_{T(3)}$	$\sigma_{T(4)}$	$\sigma_{T(5)}$	$\sigma_{Q(1)}$	$\sigma_{Q(2)}$
C1[a]	–3704.6	43.7	–603.4	–580.2	–557.4	–578.4	–580.0	–1812.3	–1846.5
C4[a]	–159.0	43.1	103.2	8.7	–84.9	9.2	9.2	–129.2	11.9
C5[b]	–226.4	49.6	87.8	4.5	–79.2	4.9	4.3	–150.0	–24.7
C3[b]	–398.8	41.1	–201.9	–32.8	136.4	–32.8	–32.7	–52.7	–306.5
C2[a]	1269.9	6.0	238.5	215.8	192.8	213.8	215.7	619.7	654.3
C6[b]	–36.6	13.5	25.3	4.7	–15.4	5.2	5.1	–27.3	3.2
C7[b]	132.0	132.9	133.2	132.8	132.4	132.9	132.8	132.2	132.8
ΔE / cm^{-1}	619.0	[c]	206.1	205.5	205.6	206.3	618.1	411.8	411.1

[a] Average of six equivalent sites in the model. [b] Average of three equivalent sites in the model. [c] Singlet(1) - Singlet(6): 0.0, –, 0.8, –, 413.0, 412.8 (where – denotes omitted value).

Table S34. Unscaled ^{13}C shifts (in ppm) for C1, C2 and C3 in dehydrated models containing different numbers of dimers with almost equal intradimer coupling ~220 cm^{-1} (CAM-B3LYP/II//GFN2-xTB), using **Eqs. S3**, **S15**, **S26 or S26** for models **M4**, **D1**, **Ra1** and **Ri1**, respectively. Shifts for sites that are not found in HKUST-1 are not reported.

T / K	288.2	298.2	313.2	328.2	348.2
model **M4**					
C1	1167.4	1151.1	1126.5	1102.1	1070.1
C2	–292.4	–284.8	–273.4	–262.0	–247.2
C3	213.4	212.2	210.4	208.7	206.4
model **D1**					
C1	974.2	967.4	955.9	943.4	925.6
C3	247.5	246.6	245.1	243.5	241.2
C2	–176.0	–173.0	–168.0	–162.6	–154.9
model **Ra1**					
C1	931.8	926.4	917.1	906.9	892.1
C3	238.2	237.6	236.5	235.4	233.7
C2	–121.7	–119.6	–116.1	–112.1	–106.4
model **Ri1**					
C1	899.4	893.6	884.0	873.6	858.7
C3	189.2	188.9	188.3	187.7	186.8
C2	–149.2	–146.7	–142.5	–137.9	–131.3
Exp. activated HKUST-1					
C1	788.4	786.4	794.2	787.4	791.2
C3	226.9	227.5	227.6	227.7	227.2
C2	–85.6	–86.2	–87.4	–85.8	–84.8

S6. References

S1. H. EL MKami, M. I. H. Mohideen, C. Pal, A. McKinley, O. Scheimann, R. E. Morris, *Chem. Phys. Lett.*, 2012, **544**, 17-21.
S2. D. M. Dawson, C. E. F. Sansome, L. N. McHugh, M. J. McPherson, L. J. McCormick McPherson, R. E. Morris and S. E. Ashbrook, *Solid State Nucl. Magn. Reson.*, 2019, **101**, 44-50.
S3. D. M. Dawson, PhD Thesis, University of St Andrews, 2014.
S4. R. L. Martin, P. J. Hay and L. R. Pratt, *J. Phys. Chem. A*, 1998, **102**, 3565–3573.
S5. Z. Ke, L. E. Jamieson, D. M. Dawson, S. E. Ashbrook and M. Bühl, *Solid State Nucl. Magn. Reson.*, 2019, **101**, 31-37.
